# Supplementary material for: A critical period of prehearing spontaneous Ca2+ spiking is required for hair‐bundle maintenance in inner hair cells
Source: EMBO J. 2023 Jan 3;42(4):e112118. doi: 10.15252/embj.2022112118 (PMC9929643; doi:10.15252/embj.2022112118)
Supplement: Supplementary file 1 — Appendix [file EMBJ-42-e112118-s001.pdf]

A critical period of spontaneous spiking is required for hair-bundle maintenance in inner hair cells

Adam J. Carlton, Jing-Yi Jeng, Fiorella Grandi, Francesca De Faveri, Federico Ceriani, Lara De Tomasi, Anna Underhill, Stuart L. Johnson, Kevin Legan, Corné J. Kros, Guy P Richardson, Mirna Mustapha, Walter Marcotti

This document includes the following documents:

Appendix Figure S1
Appendix Figure S2
Appendix Figure S3

Appendix Figure S1

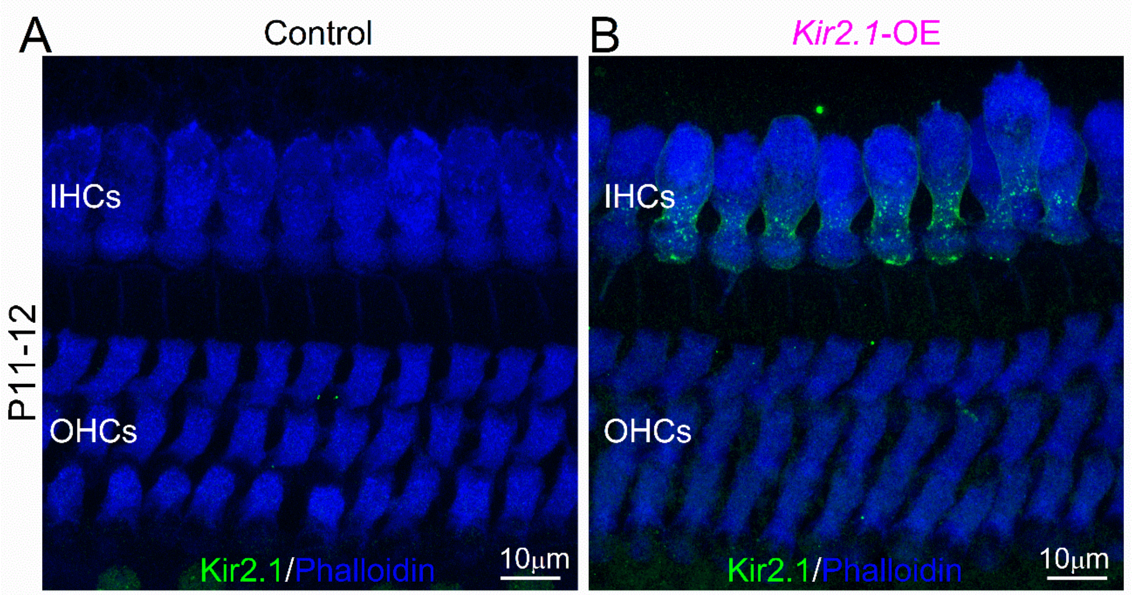

Appendix Figure S1: Outer hair cells do not express Kir2.1 channels.

A,B, Representative maximum intensity projections of confocal z-stacks showing the IHCs and OHCs of the apical cochlear region from control (**A**) and *Kir2.1*-OE (**B**) mice at postnatal day 11-P12. Hair cells were stained with antibodies against the K^+ channel Kir2.1 (green) and Phalloidin (blue: cell marker). Scale bars: 10 µm.

Appendix Figure S2

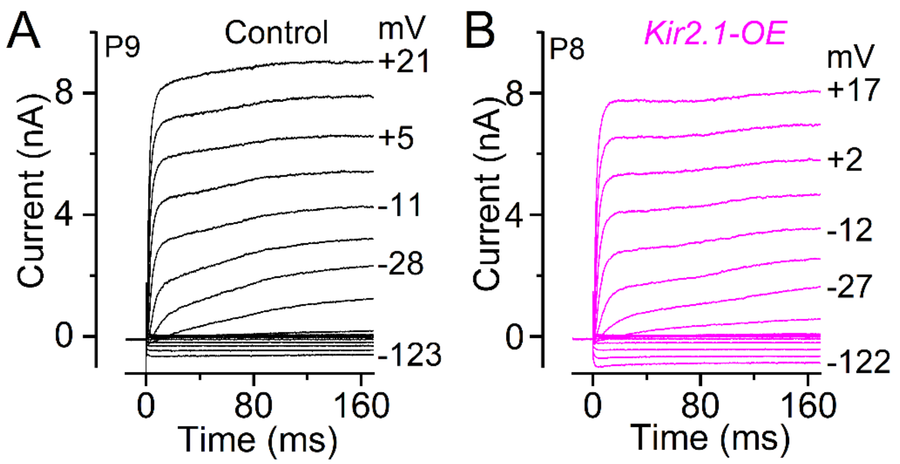

Appendix Figure S2. Basolateral membrane currents in the IHCs from mice in which DOX was removed at P5

A, B, Currents from IHCs of control (**A**, P9) and *Kir2.1*-OE (**B**, P8) pre-hearing mice; these recordings are from the same IHCs shown in **Fig. 7C,D**. Currents were elicited by using depolarizing and hyperpolarizing voltage steps, with a nominal increment of 10 mV, from a holding potential of −84 mV. Test potentials are shown next to some of the traces. Note the very similar current profile between the IHCs from both genotypes.

Appendix Figure S3

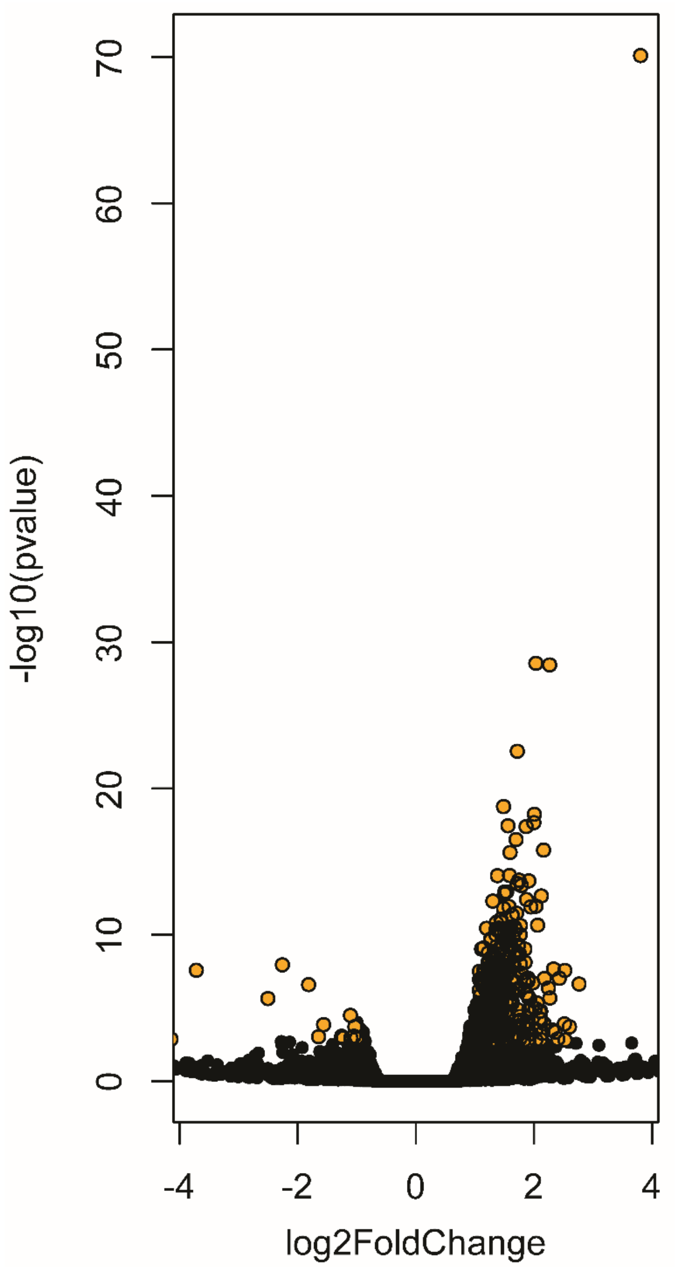

Appendix Figure S3. Volcano Plot of Differentially Expressed Genes in RNA-sequencing. Volcano plot of differentially expressed genes. Each point represents a gene. Points colored in yellow represent those that passed the differential expression analysis thresholds. The point at the highest value is *Kir2.1*.
