# Supplementary material for: A critical period of prehearing spontaneous Ca2+ spiking is required for hair‐bundle maintenance in inner hair cells
Source: EMBO J. 2023 Jan 3;42(4):e112118. doi: 10.15252/embj.2022112118 (PMC9929643; doi:10.15252/embj.2022112118)
Supplement: Supplementary file 2 — Expanded View Figures PDF [file EMBJ-42-e112118-s012.pdf]

Expanded View Figures

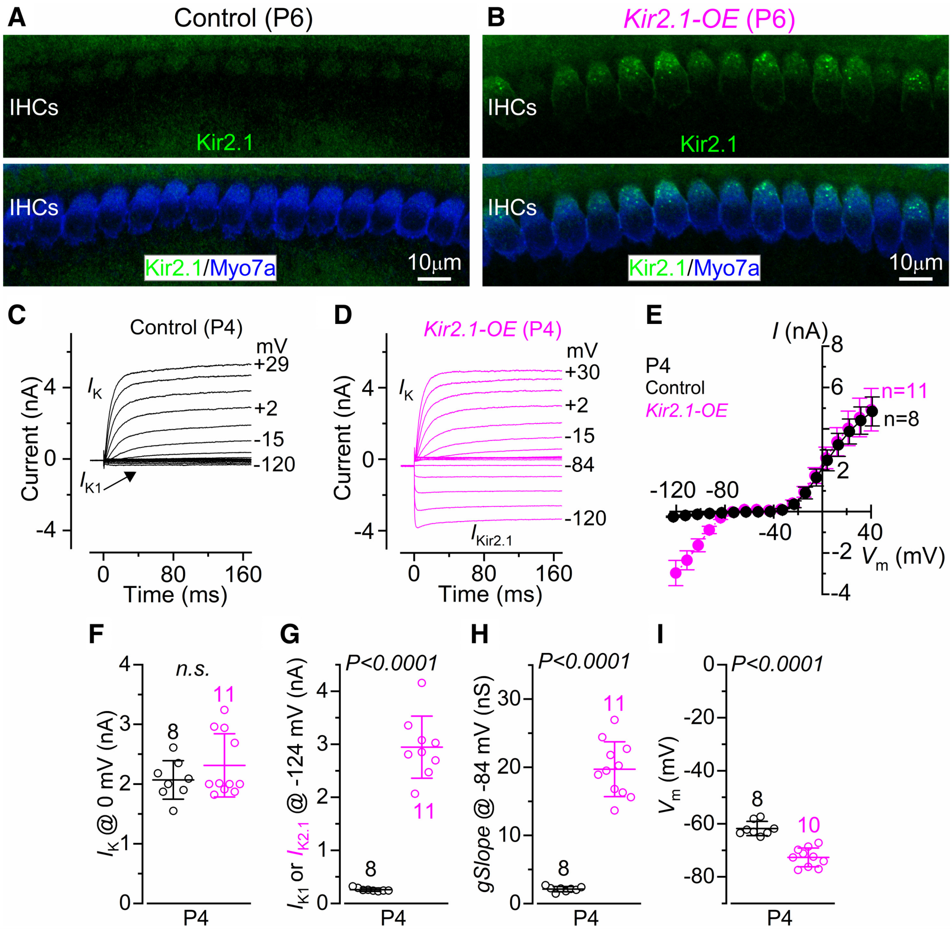

Figure EV1. Basolateral membrane properties of P4 IHCs overexpressing Kir2.1 channels.

A, B Maximum intensity projections of confocal *z*-stacks showing the inner hair cells (IHCs) of the apical cochlear region from control (A) and littermate *Kir2.1* overexpressing (B, *Kir2.1*-OE) mice at postnatal day 6 (P6). IHCs were stained with antibodies against Kir2.1 (green) and the hair cell marker Myo7a (blue). At least 3 mice for each genotype were used. Scale bars: 10 μm.

C, D Currents from IHCs of control (C) and *Kir2.1*-OE (D) prehearing P4 mice. Currents were elicited by using depolarizing and hyperpolarizing voltage steps (10 mV nominal increment), from a holding potential of −84 mV. Test potentials are shown next to some of the traces. Note that the large inward rectifier Kir2.1 current is only present in the IHCs of the *Kir2.1*-OE mouse (D). The outward current is primarily carried by a delayed rectifier current I_K. I_{K1} identifies the small inwardly rectifying K^+ current normally expressed in immature IHCs.

E Steady-state current–voltage curves obtained from IHCs of control and *Kir2.1*-OE P4 mice.

F, G Size of the total steady-state outward (F, I_K: Control 2.07 ± 0.32 nA, $n = 8$; *Kir2.1*-OE 2.31 ± 0.53 nA, $n = 11$) and inward (G, Control, I_{K1}: 0.26 ± 0.03 nA, $n = 8$; *Kir2.1*-OE, $I_{Kir2.1}$: 2.97 ± 0.61 nA, $n = 11$) K^+ currents from P4 IHCs measured at 0 mV and −124 mV, respectively. n.s: $P = 0.2836$.

H Slope conductance of the current measured at −84 mV (Control 2.12 ± 0.38 nS, $n = 8$; *Kir2.1*-OE 19.71 ± 4.03 nA, $n = 11$).

I Resting membrane potential (V_m) measured in IHCs from control (−61.8 ± 2.7 mV, $n = 8$) and *Kir2.1*-OE (−72.7 ± 3.6 mV, $n = 10$).

Data information: In panels F–I, data are shown as means ± SD, and the single cell value recordings (open symbols) are plotted with the average data. The number of IHCs investigated is shown above the average data points. All statistical tests were performed using the Student's *t*-test.
Source data are available online for this figure.

© 2023 The Authors

Figure EV2. The morphology and function of the synaptic machinery in IHCs are not affected by Kir2.1 overexpression.

A, B Maximum intensity projections of confocal *z*-stacks of IHCs taken from the apical cochlear region (9–12 kHz) of control (A) and *Kir2.1*-OE (B) mice at P11 using antibodies against CtBP2 (ribbon synaptic marker: red) and GluR2 (postsynaptic receptor marker: green). Myosin 7a (Myo7a) was used as the IHC marker (blue). At least 3 mice for each genotype were used. Scale bar 10 μm.

C, D Number of CtBP2 and GluR2 puncta in IHCs from control and *Kir2.1*-OE (C) and co-localized CtBP2 and GluR2 puncta in IHCs from both genotypes (D). Data are plotted as mean values (lines) and individual CtBP2 and GluR2 counts or colocalized counts (smaller open symbols). Numbers above or below the data in panels represent the IHCs (and mice) used for each time point. All comparisons were not significantly different, and *P*-values are shown below the data and were obtained using the Student's *t*-test. Average values are mean ± SD.

E, F Calcium current (I_{Ca}) and corresponding changes in membrane capacitance (ΔC_m) recorded from IHCs of control and *Kir2.1-OE* mice. Recordings were obtained in response to 50 ms voltage steps (10 mV increments) from −81 mV and using 1.3 mM extracellular Ca^{2+} and at body temperature. For clarity, only maximal responses at −11 mV are shown. Average peak I_{Ca} (bottom) and ΔC_m (top) curves from IHCs of control (black) and *Kir2.1-OE* (magenta) mice (F). Average values are mean ± SD.

Source data are available online for this figure.

 © 2023 The Authors

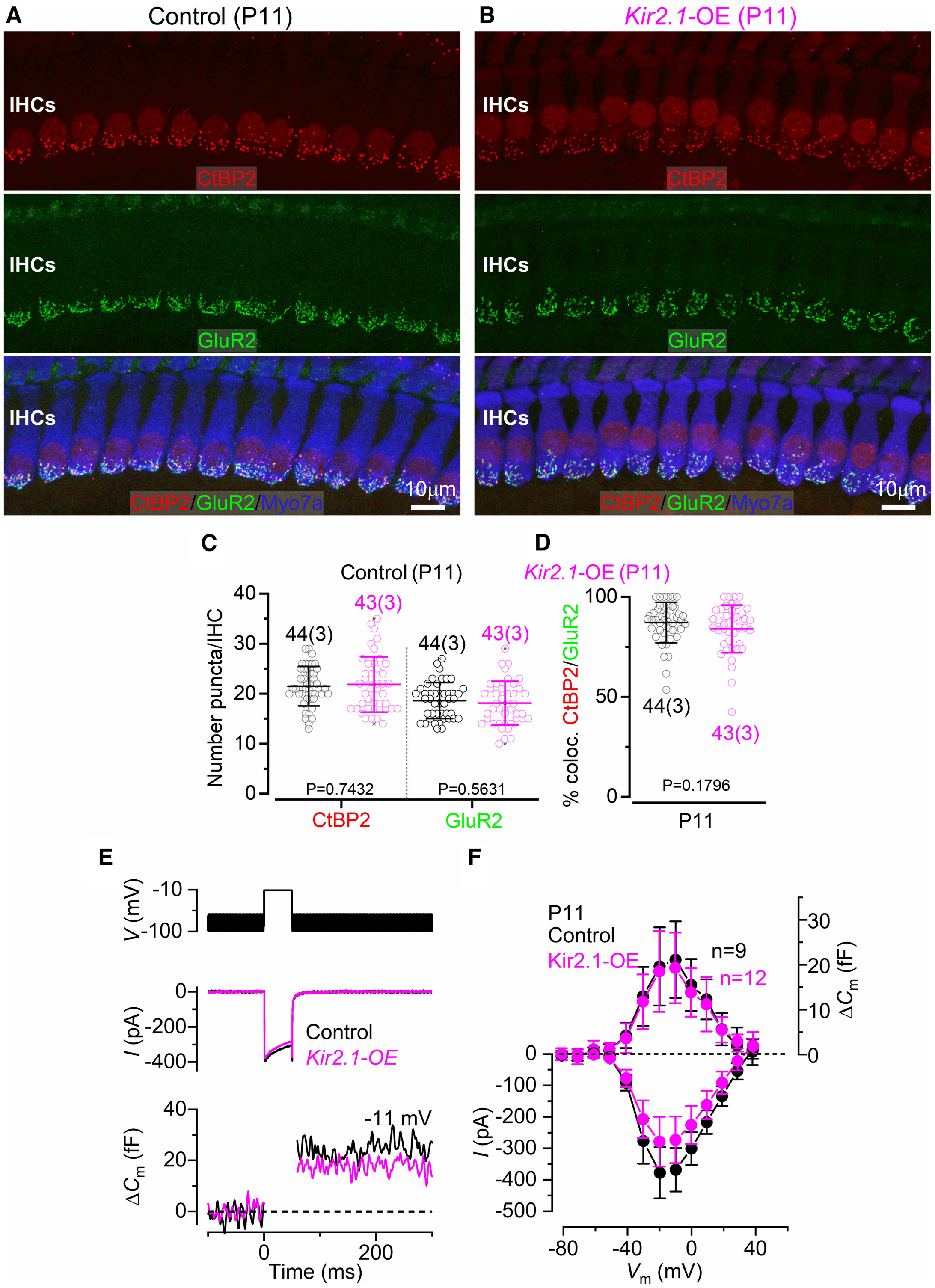
A
Control (P11)
B
Kir2.1-OE (P11)
IHCs
CtBP2
GluR2
CtBP2/GluR2/Myo7a
10μm
C
D
Number puncta/IHC
44(3)
43(3)
P=0.7432
P=0.5631
CtBP2
GluR2
% coloc. CtBP2/GluR2
P=0.1796
P11
E
V (mV)
I (pA)
Control
Kir2.1-OE
ΔCm (fF)
-11 mV
Time (ms)
F
P11
n=9
n=12
Vm (mV)

Figure EV2.

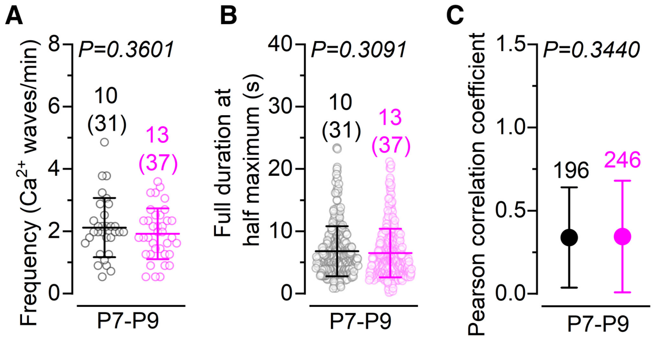

Figure EV3. Ca^{2+} signals in IHCs are correlated with the Ca^{2+} waves in nonsensory cells.

A, B Frequency (A) and full duration at half maximum (B) of the Ca^{2+} waves recorded from P7-P9 control and *Kir2.1*-OE mice. The full duration at half maximum measures how long the Ca^{2+} waves last in time, which was measured as the duration of the Ca^{2+} trace at half the peak. Data are shown as means ± SD and single data points are shown as open circles. The number above the data represents the number of mice tested and, in between the brackets, the number of recordings. Note that for the frequency (A) each data point represents one recording (usually 2–3 recordings per mouse). For the full duration at half maximum (B), we plotted one data point for each of the Ca^{2+} waves recorded from both control (365 Ca^{2+} waves) and *Kir2.1*-OE (395 Ca^{2+} waves) mice. *P*-values shown above the panels were calculated using the Student's *t*-test.

C Average Pearson correlation coefficient between the Ca^{2+} activity in individual IHCs and the Ca^{2+} waves in the nonsensory cells from control and *Kir2.1-OE* mice (10 and 13, respectively). Numbers of IHCs analyzed are shown above the data. Data are shown as means ± SD. The correlation coefficients for both control and *Kir2.1-OE* mice were significantly different from zero ($P < 0.001$, Wilcoxon rank-sum test). There was no significant difference between control and *Kir2.1-OE* mice ($P = 0.3440$, Mann–Whitney U test). For calculating descriptive statistics and tests of significance, Pearson correlation coefficients were transformed using Fisher transformation (inverse hyperbolic tangent).

Source data are available online for this figure.

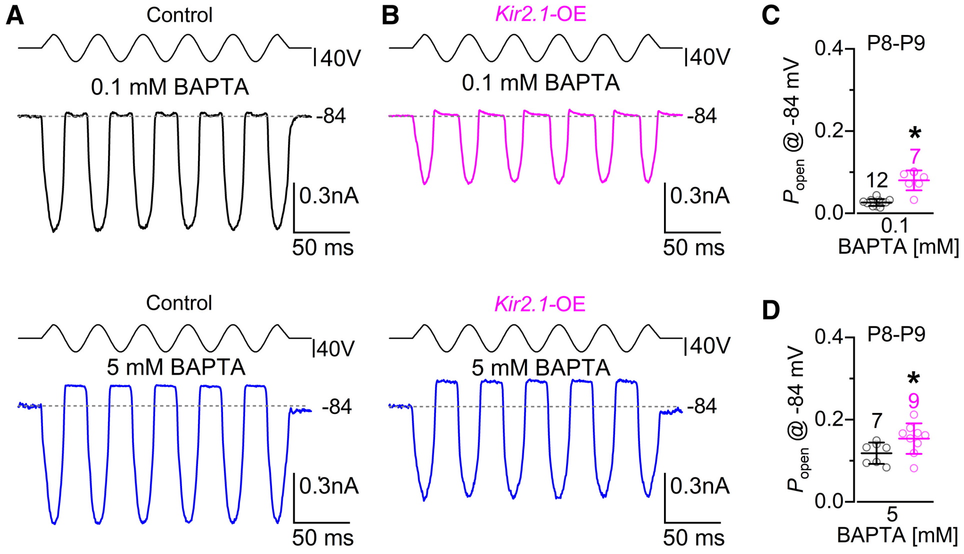

Figure EV4. Calcium sensitivity of the MET current is reduced in Kir2.1 overexpressing IHCs at the start of the second postnatal week.

A, B Saturating MET currents recorded from apical IHCs of P8 control (A) and *Kir2.1*-OE (B) mice in the presence of 0.1 mM (upper panels) and 5 mM (lower panels) of the fast Ca^{2+} chelator BAPTA. Responses were obtained in response to 50 Hz sinusoidal force stimuli to the hair bundles at membrane potentials of −84 mV. Driver voltage (DV) stimuli to the fluid jet are shown above the traces, with positive deflections of the DV being excitatory.

C, D Resting open probability (P_{open}) of the MET current in IHCs from the two genotypes (control: black; *Kir2.1*-OE: magenta) measured at −84 mV in 0.1 mM BAPTA (C) and 5 mM BAPTA (D). The P_{open} was found significantly different between the two genotypes (C: $^*P < 0.0001$; D: $^*P = 0.0496$, *t*-test). The increased resting P_{open} using a fixed BAPTA concentration indicates a reduced Ca^{2+} sensitivity of the MET channel in the IHCs of *Kir2.1*-OE mice. In panels C and D, data are shown as means ± SD, and the single cell value recordings (open symbols) are plotted behind the average data. The number of IHCs investigated is shown above the average data points from 5 control and 5 littermates *Kir2.1*-OE mice.

Source data are available online for this figure.

 © 2023 The Authors

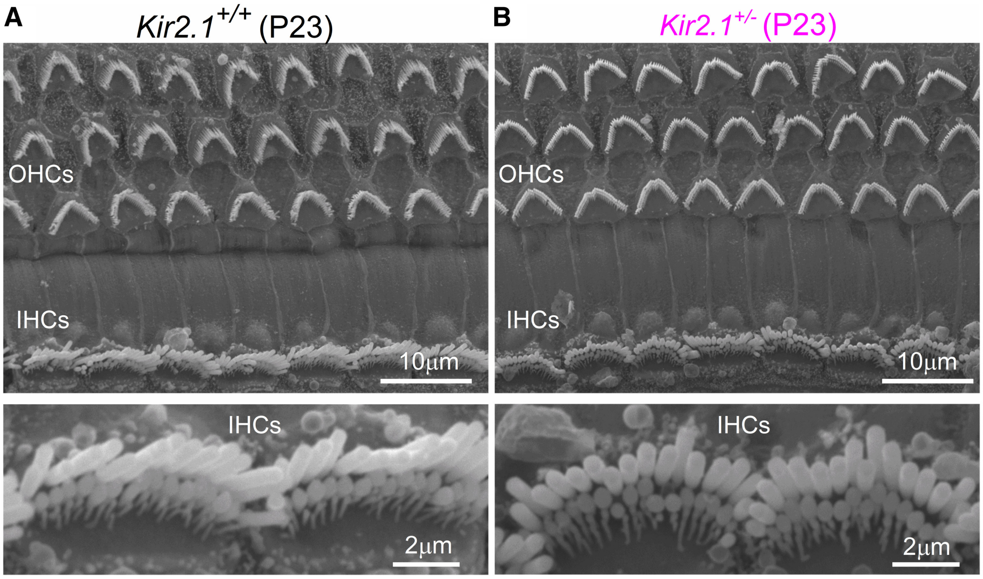

Figure EV5. IHC bundle morphology in Kir2.1 mice.

A, B Scanning electron micrographs showing the typical hair-bundle structure of apical-coil IHCs and OHCs in *Kir2.1* P23 mice (without pairing them with *Otof*$^{tTA+/-}$ mice) fed with DOX in the drinking water throughout their life. This result shows that the application of DOX to heterozygous *Kir2.1* mice (without crossing with *Otof*^{tTA} mice) does not affect the hair-bundle morphology in adult mice. At least 3 mice for each genotype were used.

Source data are available online for this figure.

© 2023 The Authors
