# Supplementary material for: A critical period of prehearing spontaneous Ca2+ spiking is required for hair‐bundle maintenance in inner hair cells
Source: EMBO J. 2023 Jan 3;42(4):e112118. doi: 10.15252/embj.2022112118 (PMC9929643; doi:10.15252/embj.2022112118)
Supplement: Supplementary file 5 — Source Data for Expanded View [file EMBJ-42-e112118-s004.zip › Figure Source Data_EMBOJ-2022-112118/Expanded View Figure_EV1/Figure EV1E-I.docx]

**Figure EV1E**

| **Control** | | | |  | **Kir2.1-OE** | | | |
| --- | --- | --- | --- | --- | --- | --- | --- | --- |
| **Vm** | **IK** | **SD** | **N** |  | **Vm** | **IK** | **SD** | **N** |
| -122.41901 | -250.4851 | 30.20982 | 8 |  | -120.41466 | -2969.82091 | 613.29741 | 11 |
| -112.49921 | -189.34004 | 24.52609 | 8 |  | -111.16419 | -2351.87732 | 460.51746 | 11 |
| -102.56824 | -137.39292 | 16.34203 | 8 |  | -102.03964 | -1629.3535 | 309.47771 | 11 |
| -92.62727 | -92.85766 | 13.65853 | 8 |  | -92.93451 | -887.24046 | 177.30104 | 11 |
| -82.66957 | -60.87275 | 7.69187 | 8 |  | -83.63724 | -302.72448 | 80.99583 | 11 |
| -72.69848 | -39.18412 | 7.19061 | 8 |  | -73.98192 | -16.72067 | 41.41742 | 11 |
| -62.72336 | -20.47321 | 7.56573 | 8 |  | -64.11283 | 91.51455 | 41.12083 | 11 |
| -52.73443 | -11.98532 | 5.87908 | 8 |  | -54.11741 | 95.69472 | 32.21484 | 11 |
| -42.75006 | 0.50158 | 14.32078 | 8 |  | -44.07297 | 59.32139 | 18.49212 | 11 |
| -32.84195 | 74.49839 | 69.31475 | 8 |  | -34.13364 | 110.3665 | 38.78395 | 11 |
| -23.19624 | 351.38347 | 164.28056 | 8 |  | -24.48696 | 405.04077 | 154.53007 | 11 |
| -13.90174 | 898.00275 | 285.9471 | 8 |  | -15.14181 | 952.53956 | 258.28809 | 11 |
| -4.86669 | 1643.93239 | 409.19528 | 8 |  | -6.07857 | 1739.62221 | 382.59498 | 11 |
| 4.04876 | 2477.66918 | 506.24429 | 8 |  | 2.90635 | 2594.53711 | 525.87955 | 11 |
| 13.0643 | 3226.39869 | 558.56867 | 8 |  | 11.97555 | 3377.33851 | 679.56379 | 11 |
| 22.19909 | 3873.51184 | 602.41701 | 8 |  | 21.20624 | 4023.20665 | 832.6958 | 11 |
| 31.60648 | 4400.28582 | 658.13076 | 8 |  | 30.57451 | 4549.65603 | 934.44958 | 11 |
| 41.01559 | 4840.47394 | 700.01203 | 8 |  | 40.13251 | 4924.8008 | 1026.3761 | 11 |

**Figure EV1F-I**

| **Control** | | | |  | **Kir2.1-OE** | | | |
| --- | --- | --- | --- | --- | --- | --- | --- | --- |
| **Figure EV1F** | **Figure EV1G** | **Figure EV1H** | **Figure 1I** |  | **Figure 1F** | **Figure 1G** | **Figure 1H** | **Figure 1I** |
| **IK** | **IK1** | **gSlope** | **Vm** |  | **IK,D** | **IKir2.1** | **gSlope** | **Vm** |
| 2.35 | 240 | 1.8 | -57.3 |  | 2 | 2378 | 13.65 | -76.2 |
| 1.55 | 255 | 2.2 | -64.9 |  | 1.91 | 2852 | 16.8 | -77.4 |
| 1.87 | 226 | 2.15 | -63 |  | 2.01 | 3077 | 20.3 | -77.1 |
| 1.9 | 252 | 1.45 | -58.1 |  | 1.82 | 2472 | 19.5 | -72.4 |
| 2 | 242 | 2 | -63.4 |  | 2 | 2065 | 16.3 | -71.2 |
| 2.09 | 292 | 2.25 | -62.2 |  | 2.94 | 2808 | 18.9 | -74.1 |
| 2.18 | 241 | 2.4 | -61.8 |  | 2.96 | 4155 | 26.95 | -68.6 |
| 2.61 | 321 | 2.7 | -63.5 |  | 2.69 | 3353 | 21.8 | -69.8 |
|  |  |  |  |  | 2 | 2696 | 15.6 |  |
|  |  |  |  |  | 1.87 | 3025 | 22.7 | -67.2 |
|  |  |  |  |  | 3.24 | 3786 | 24.4 | -72.7 |
