# Supplementary material for: A critical period of prehearing spontaneous Ca2+ spiking is required for hair‐bundle maintenance in inner hair cells
Source: EMBO J. 2023 Jan 3;42(4):e112118. doi: 10.15252/embj.2022112118 (PMC9929643; doi:10.15252/embj.2022112118)
Supplement: Supplementary file 5 — Source Data for Expanded View [file EMBJ-42-e112118-s004.zip › Figure Source Data_EMBOJ-2022-112118/Expanded View Figure_EV2/Figure EV2C,D,F.docx]

| **Figure EV2C** | | | | | | |
| --- | --- | --- | --- | --- | --- | --- |
|  | **Control** | | | **Kir2.1-OE** | | |
|  | **Mean** | **SD** | **N** | **Mean** | **SD** | **N** |
| CtBP2 | 21.5 | 3.93848 | 44 | 21.83721 | 5.51591 | 43 |
| GluR2 | 18.61364 | 3.57781 | 44 | 18.11628 | 4.38183 | 43 |

| **Figure EV2D** | | | | | | |
| --- | --- | --- | --- | --- | --- | --- |
|  | **Control** | | | **Kir2.1-OE** | | |
|  | **Mean** | **SD** | **N** | **Mean** | **SD** | **N** |
| Colocalised CtBP2-GluR2 | 87.16663 | 10.0763 | 44 | 83.97162 | 11.89218 | 43 |

| **Figure EV2F** | | | | | | | |
| --- | --- | --- | --- | --- | --- | --- | --- |
| **Control** | | | | **Kir2.1-OE** | | | |
| **Vm** | **MeanΔCm** | **SD** | **N** | **Vm** | **Mean ΔCm** | **SD** | **N** |
| -80.98796 | -0.24508 | 1.0518 | 9 | -80.99921 | 0.04411 | 1.3617 | 12 |
| -71.0538 | -0.56641 | 0.8266 | 9 | -71.03084 | -0.50738 | 1.6912 | 12 |
| -61.12271 | 0.6039 | 1.05095 | 9 | -61.05358 | 1.15869 | 1.93914 | 12 |
| -51.10834 | 0.92657 | 1.43885 | 9 | -51.02558 | 1.27632 | 1.269 | 12 |
| -40.72726 | 4.23375 | 2.77417 | 9 | -40.72995 | 3.62481 | 3.30409 | 12 |
| -30.06745 | 12.94906 | 6.57266 | 9 | -30.25057 | 11.76103 | 6.06819 | 12 |
| -19.78951 | 19.60775 | 8.72669 | 9 | -20.00959 | 18.50787 | 9.00022 | 12 |
| -9.82696 | 21.1229 | 8.52043 | 9 | -10.01632 | 19.29215 | 7.87821 | 12 |
| -0.09958 | 15.49422 | 5.77606 | 9 | -0.21898 | 13.8129 | 5.37673 | 12 |
| 9.50444 | 12.27933 | 4.43634 | 9 | 9.46585 | 11.20698 | 6.04802 | 12 |
| 19.0346 | 5.65262 | 3.60275 | 9 | 19.11059 | 5.52098 | 2.83215 | 12 |
| 28.59008 | 2.03202 | 3.98584 | 9 | 28.76649 | 2.94442 | 1.5422 | 12 |
| 38.20282 | 0.58951 | 2.95375 | 9 | 38.4979 | 2.02999 | 2.9926 | 12 |
|  | | | | | | | |
| **Vm** | **Mean I_Ca_** | **SD** | **N** | **Vm** | **Mean I_Ca_** | **SD** | **N** |
| -80.98796 | -2.24932 | 7.34553 | 9 | -80.99921 | -0.23028 | 0.63025 | 12 |
| -71.0538 | 0.18032 | 0.37999 | 9 | -71.03084 | -0.28808 | 0.4281 | 12 |
| -61.12271 | 2.98462 | 5.32781 | 9 | -61.05358 | -1.85428 | 1.55503 | 12 |
| -51.10834 | -9.80607 | 5.54221 | 9 | -51.02558 | -13.29273 | 5.39237 | 12 |
| -40.72726 | -91.62945 | 24.71916 | 9 | -40.72995 | -77.29497 | 27.56832 | 12 |
| -30.06745 | -276.79667 | 72.28683 | 9 | -30.25057 | -207.36605 | 59.75019 | 12 |
| -19.78951 | -377.54222 | 81.67516 | 9 | -20.00959 | -278.87936 | 79.35911 | 12 |
| -9.82696 | -368.27778 | 69.06564 | 9 | -10.01632 | -273.21102 | 74.53662 | 12 |
| -0.09958 | -300.81667 | 52.3899 | 9 | -0.21898 | -225.57372 | 60.76285 | 12 |
| 9.50444 | -216.81111 | 37.07177 | 9 | 9.46585 | -161.71133 | 45.04288 | 12 |
| 19.0346 | -133.98889 | 31.14781 | 9 | 19.11059 | -91.60588 | 35.54287 | 12 |
| 28.59008 | -54.12222 | 27.1005 | 9 | 28.76649 | -22.30743 | 30.053 | 12 |
| 38.20282 | 15.66838 | 30.96108 | 9 | 38.4979 | 25.4764 | 23.85668 | 12 |
