# Supplementary material for: A critical period of prehearing spontaneous Ca2+ spiking is required for hair‐bundle maintenance in inner hair cells
Source: EMBO J. 2023 Jan 3;42(4):e112118. doi: 10.15252/embj.2022112118 (PMC9929643; doi:10.15252/embj.2022112118)
Supplement: Supplementary file 5 — Source Data for Expanded View [file EMBJ-42-e112118-s004.zip › Figure Source Data_EMBOJ-2022-112118/Expanded View Figure_EV3/Figure EV3A-C.docx]

| **Figure EV3A** | | | | | |
| --- | --- | --- | --- | --- | --- |
| **Control** | | | **Kir2.1-OE** | | |
| **Mean** | **SD** | **N** | **Mean** | **SD** | **N** |
| 2.286 | 0.94726 | 20 | 2.21294 | 0.84131 | 17 |

| **Figure EV3B** | | | | | |
| --- | --- | --- | --- | --- | --- |
| **Control** | | | **Kir2.1-OE** | | |
| **Mean** | **SD** | **N** | **Mean** | **SD** | **N** |
| 7.318 | 4.341 | 20 | 7.232 | 4.362 | 17 |

| **Figure EV3C** | | | | | |
| --- | --- | --- | --- | --- | --- |
| **Control** | | | **Kir2.1-OE** | | |
| **Mean** | **SD** | **N** | **Mean** | **SD** | **N** |
| 0.3388 | 0.3021 | 196 | 0.3446 | 0.3364 | 246 |
