# Supplementary material for: A critical period of prehearing spontaneous Ca2+ spiking is required for hair‐bundle maintenance in inner hair cells
Source: EMBO J. 2023 Jan 3;42(4):e112118. doi: 10.15252/embj.2022112118 (PMC9929643; doi:10.15252/embj.2022112118)
Supplement: Supplementary file 5 — Source Data for Expanded View [file EMBJ-42-e112118-s004.zip › Figure Source Data_EMBOJ-2022-112118/Expanded View Figure_EV4/Figure EV4C,D.docx]

| **Figure EV4C** | | | | | |
| --- | --- | --- | --- | --- | --- |
| **Control** | | | **Kir2.1-OE** | | |
| **Mean** | **SD** | **N** | **Mean** | **SD** | **N** |
| 0.02624 | 0.00833 | 12 | 0.08006 | 0.02425 | 7 |

| **Figure EV4D** | | | | | |
| --- | --- | --- | --- | --- | --- |
| **Control** | | | **Kir2.1-OE** | | |
| **Mean** | **SD** | **N** | **Mean** | **SD** | **N** |
| 0.11826 | 0.02591 | 7 | 0.15379 | 0.03713 | 9 |
