# Supplementary figures and images for: A critical period of prehearing spontaneous Ca2+ spiking is required for hair‐bundle maintenance in inner hair cells
Source: EMBO J. 2023 Jan 3;42(4):e112118. doi: 10.15252/embj.2022112118 (PMC9929643; doi:10.15252/embj.2022112118)

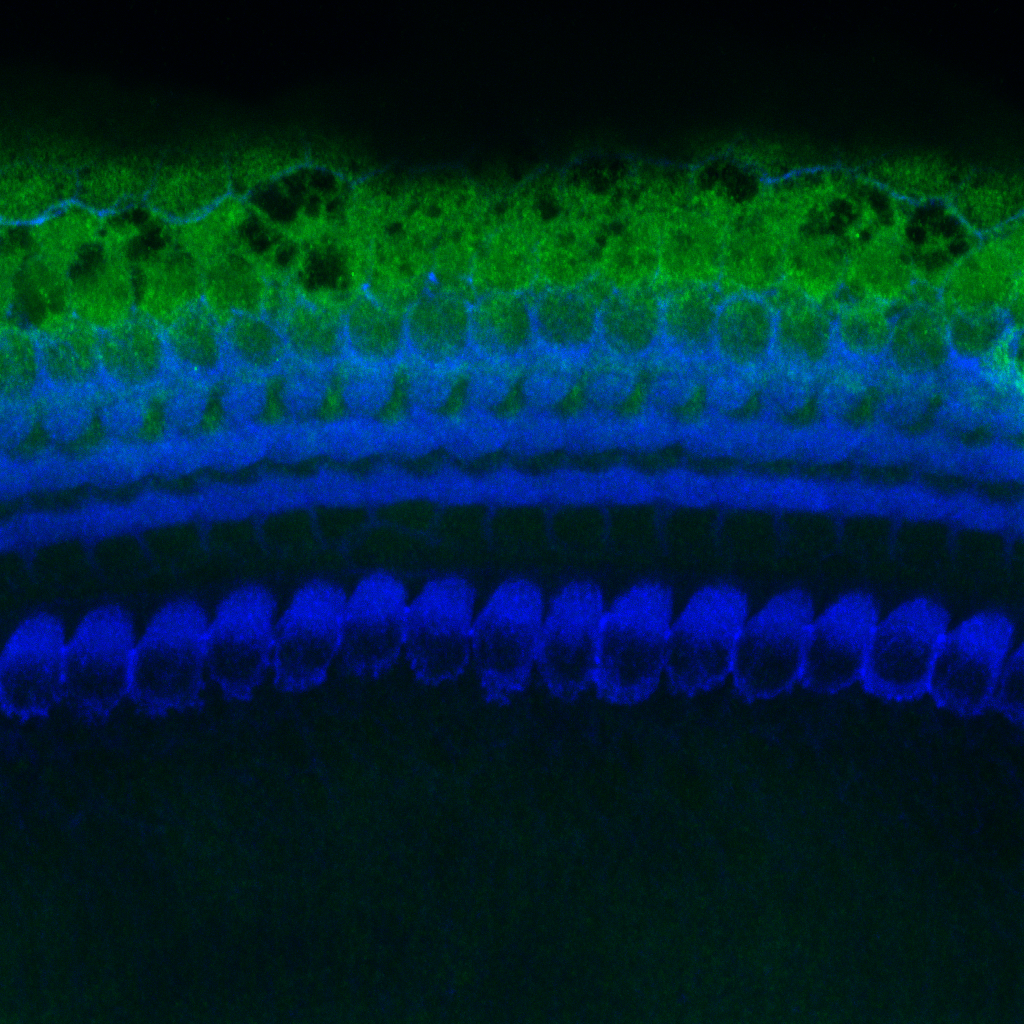

Supplement: Supplementary file 5 — Source Data for Expanded View [file EMBJ-42-e112118-s004.zip › Figure Source Data_EMBOJ-2022-112118/Expanded View Figure_EV1/Figure EV1A.tif]

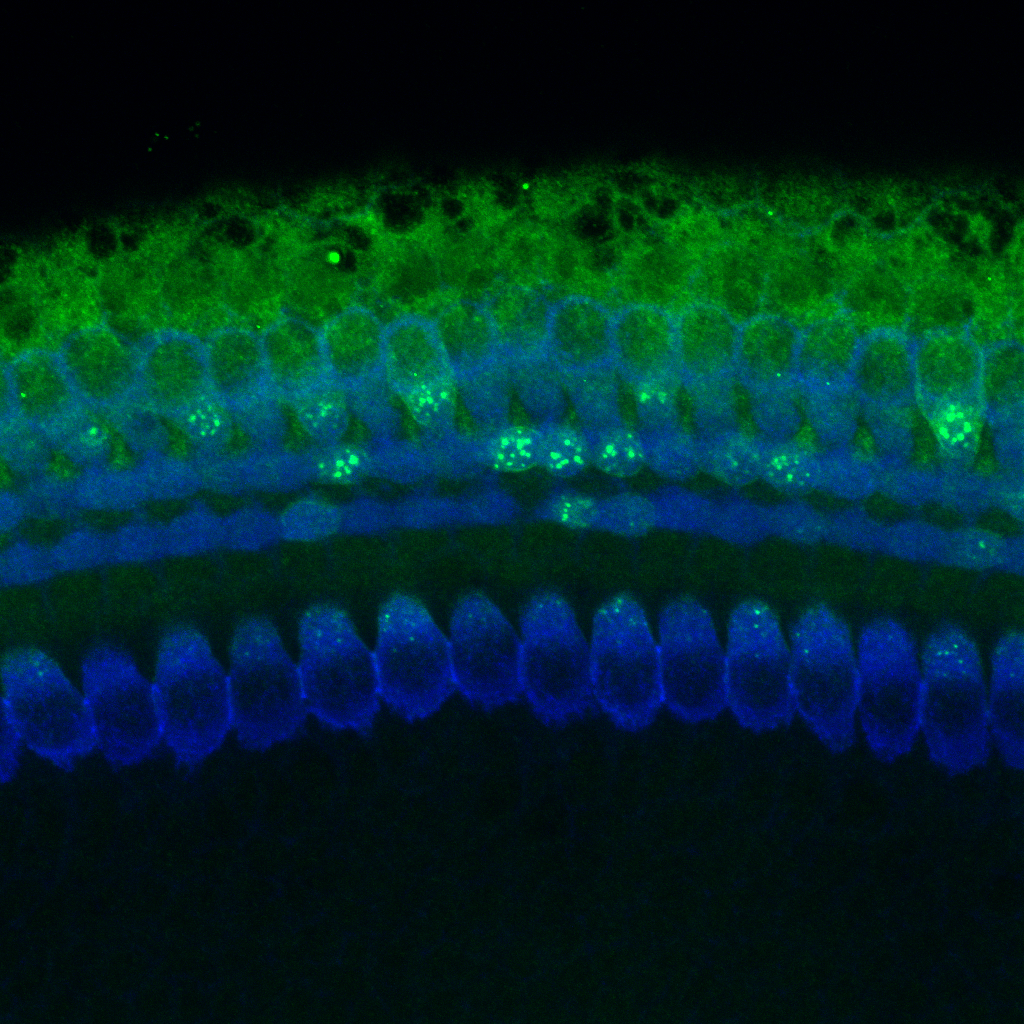

Supplement: Supplementary file 5 — Source Data for Expanded View [file EMBJ-42-e112118-s004.zip › Figure Source Data_EMBOJ-2022-112118/Expanded View Figure_EV1/Figure EV1B.tif]

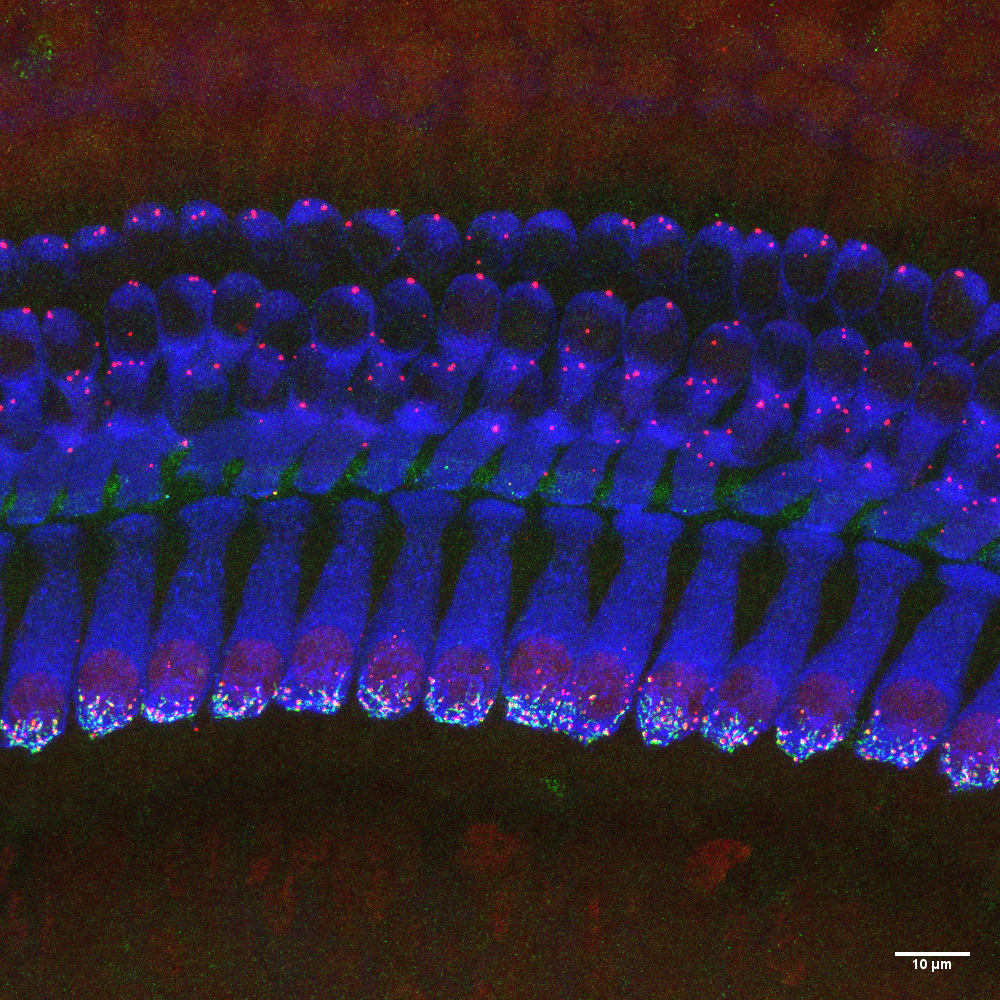

Supplement: Supplementary file 5 — Source Data for Expanded View [file EMBJ-42-e112118-s004.zip › Figure Source Data_EMBOJ-2022-112118/Expanded View Figure_EV2/Figure EV2A.tif]

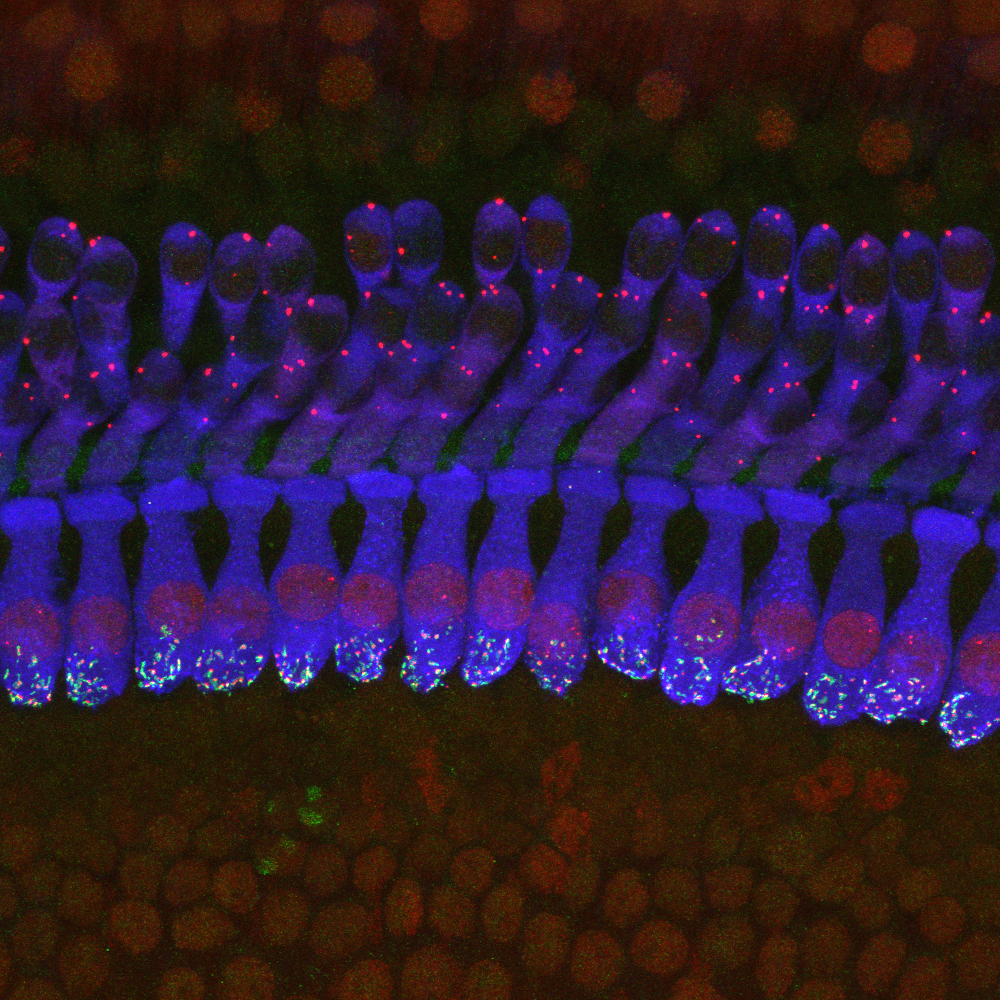

Supplement: Supplementary file 5 — Source Data for Expanded View [file EMBJ-42-e112118-s004.zip › Figure Source Data_EMBOJ-2022-112118/Expanded View Figure_EV2/Figure EV2B.tif]

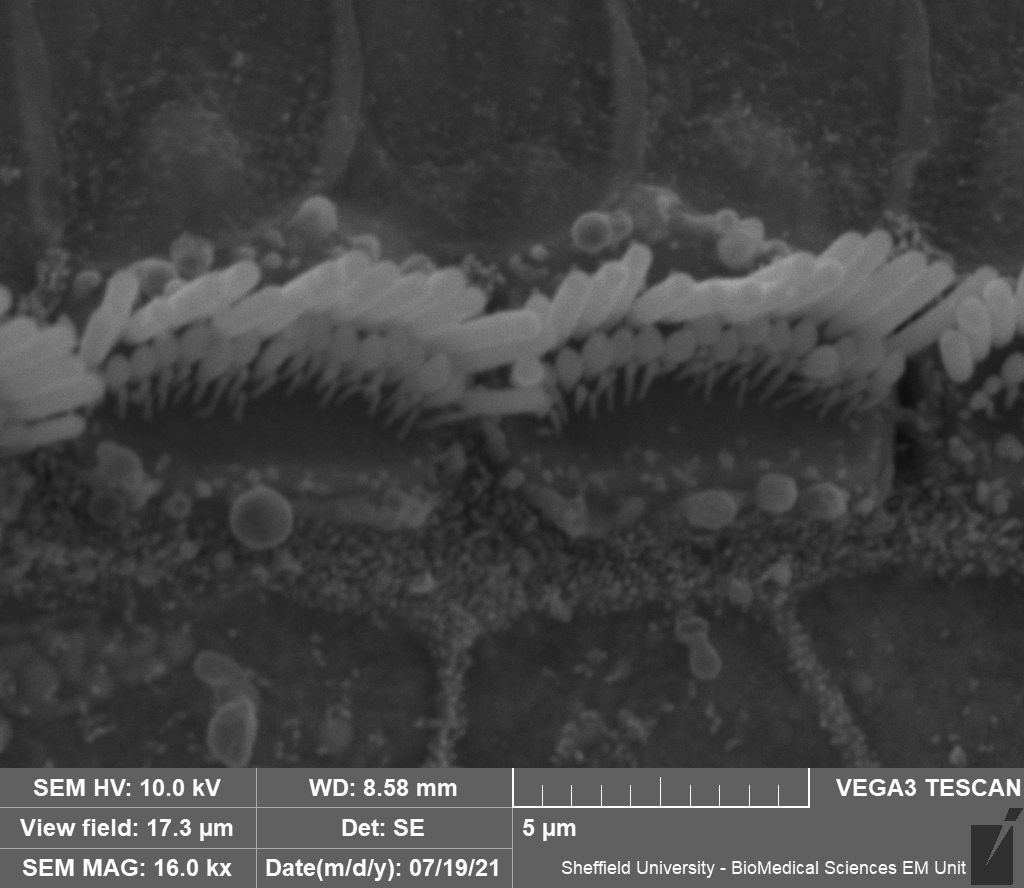

Supplement: Supplementary file 5 — Source Data for Expanded View [file EMBJ-42-e112118-s004.zip › Figure Source Data_EMBOJ-2022-112118/Expanded View Figure_EV5/Figure EV5A_Lower panel.tif]

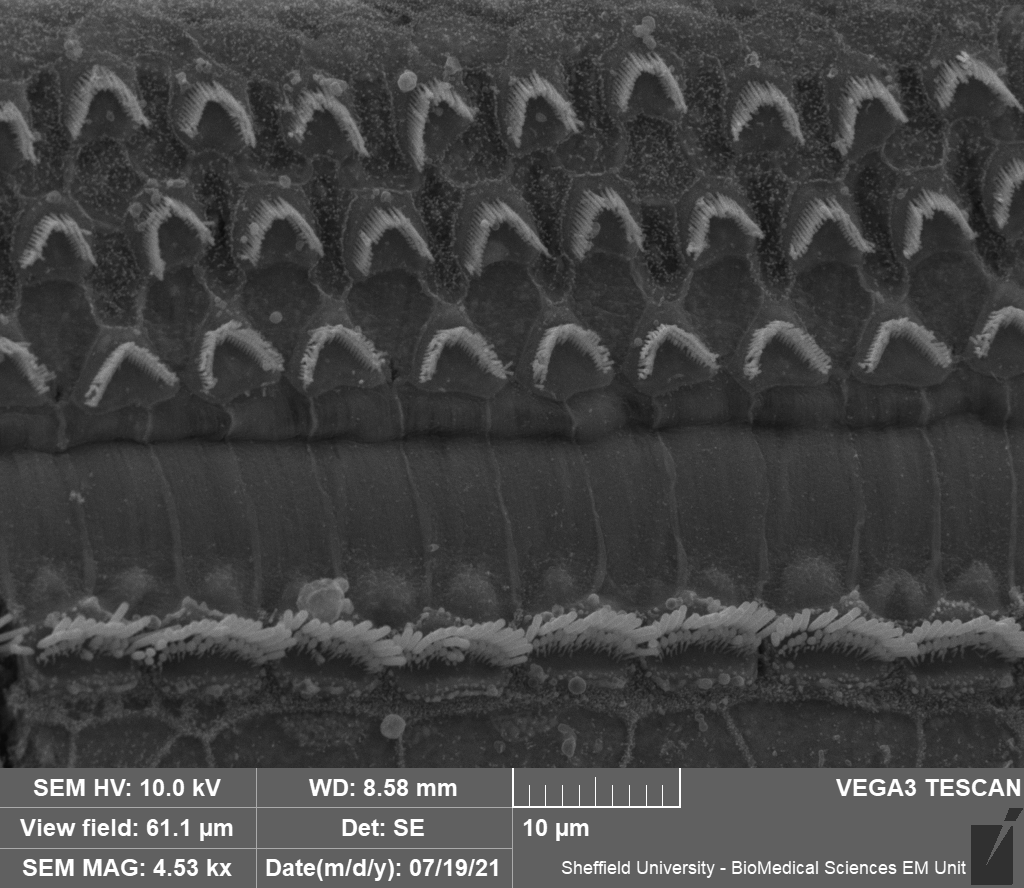

Supplement: Supplementary file 5 — Source Data for Expanded View [file EMBJ-42-e112118-s004.zip › Figure Source Data_EMBOJ-2022-112118/Expanded View Figure_EV5/Figure EV5A_Upper panel.tif]

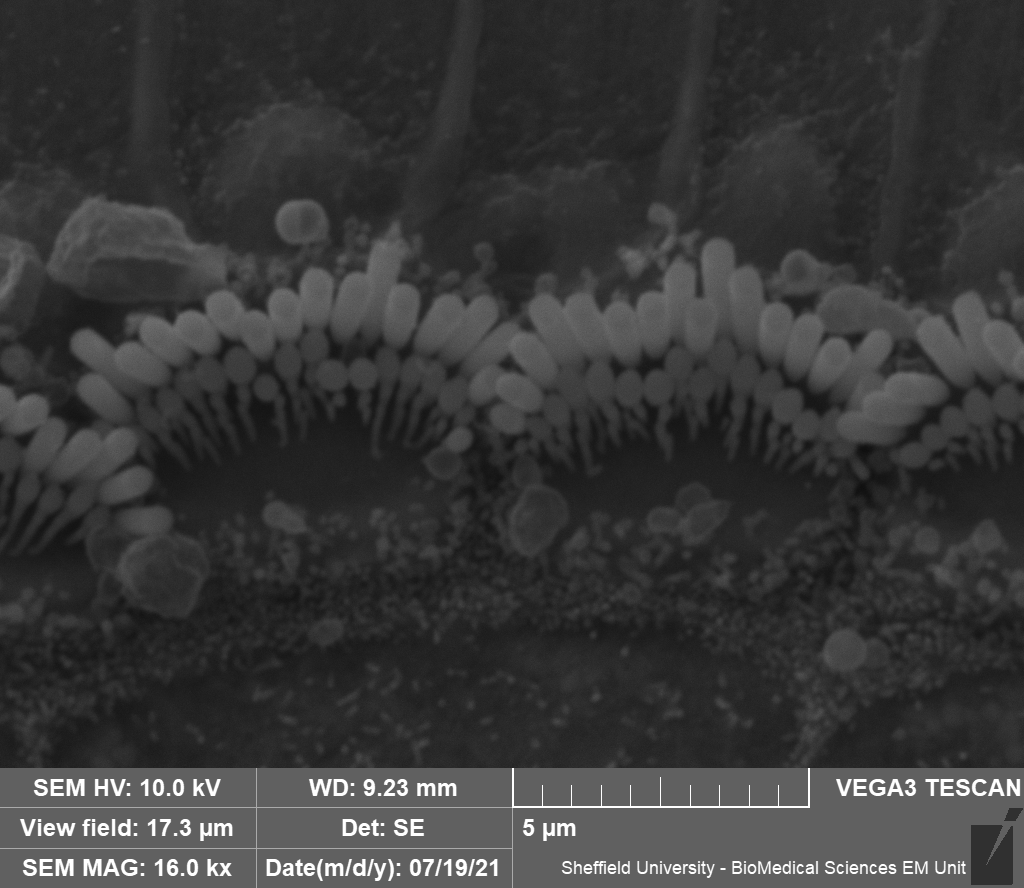

Supplement: Supplementary file 5 — Source Data for Expanded View [file EMBJ-42-e112118-s004.zip › Figure Source Data_EMBOJ-2022-112118/Expanded View Figure_EV5/Figure EV5B_Lower panel.tif]

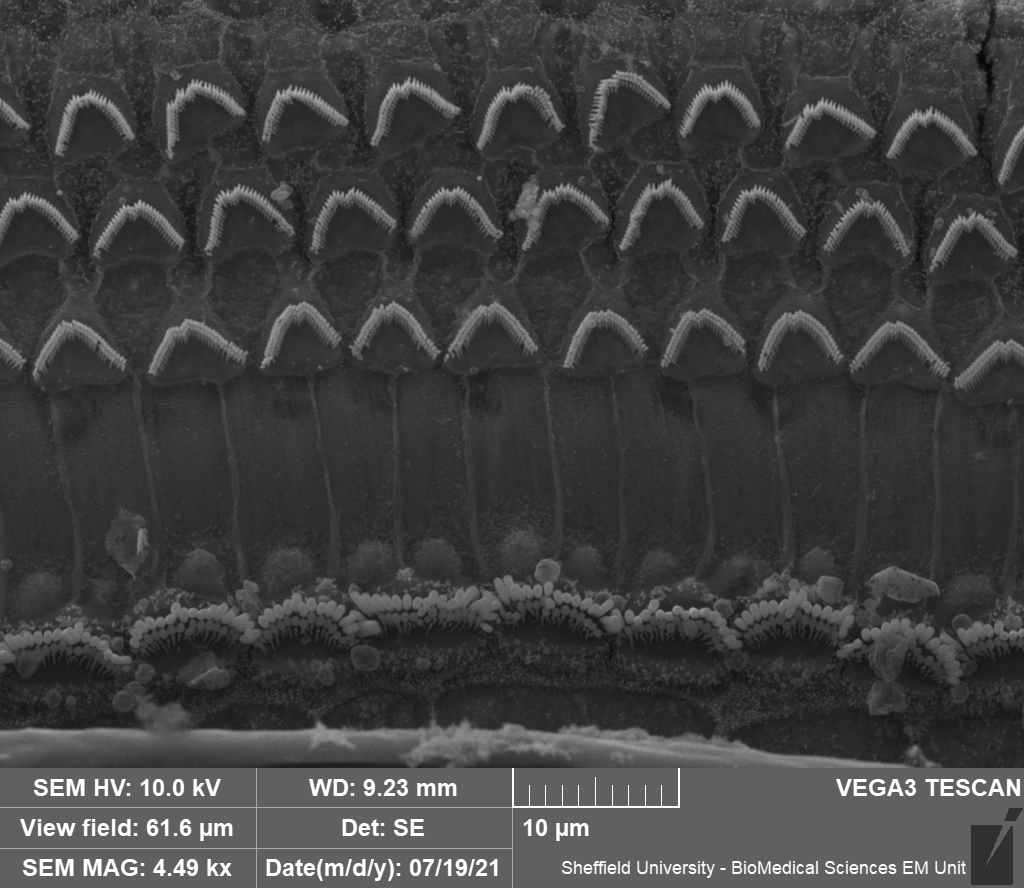

Supplement: Supplementary file 5 — Source Data for Expanded View [file EMBJ-42-e112118-s004.zip › Figure Source Data_EMBOJ-2022-112118/Expanded View Figure_EV5/Figure EV5B_Upper panel.tif]

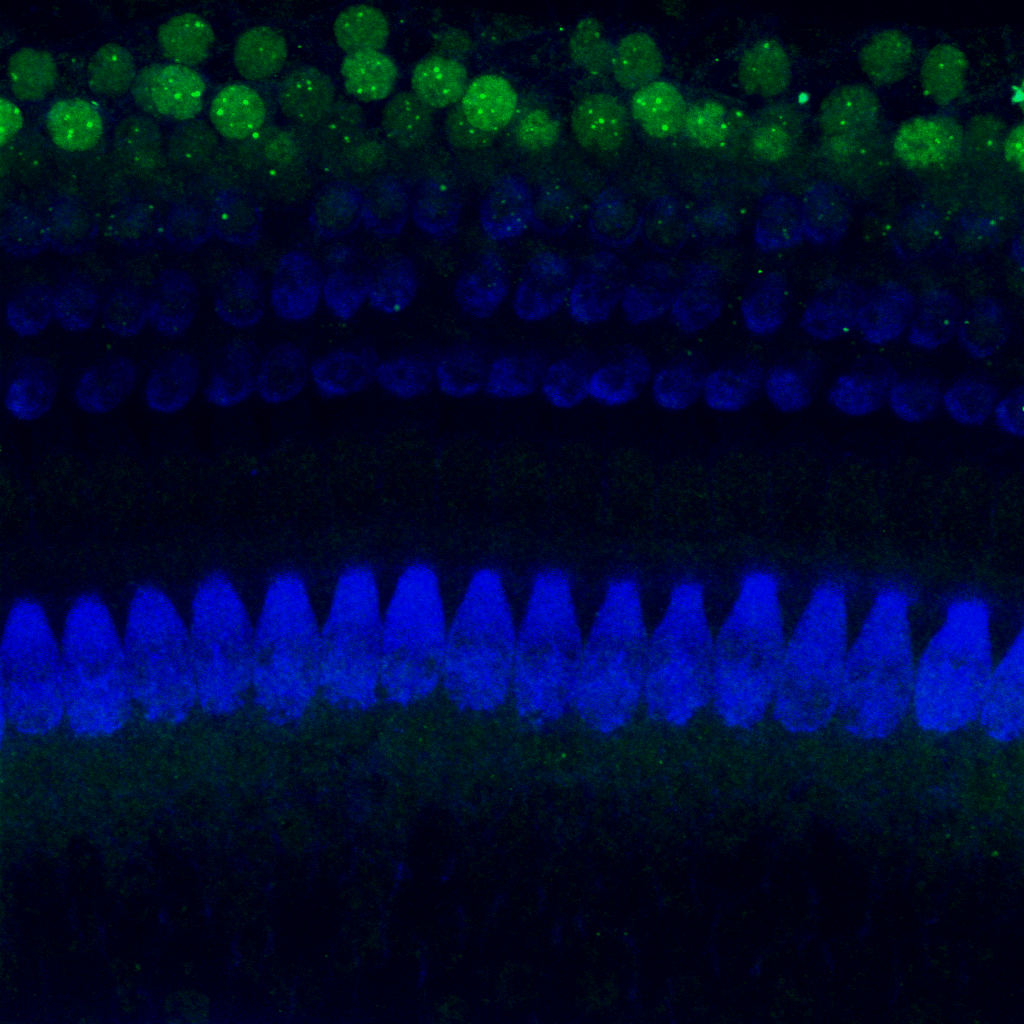

Supplement: Supplementary file 7 — Source Data for Figure 1 [file EMBJ-42-e112118-s002.zip › Figure 1/Figure 1A.tif (RGB).tif]

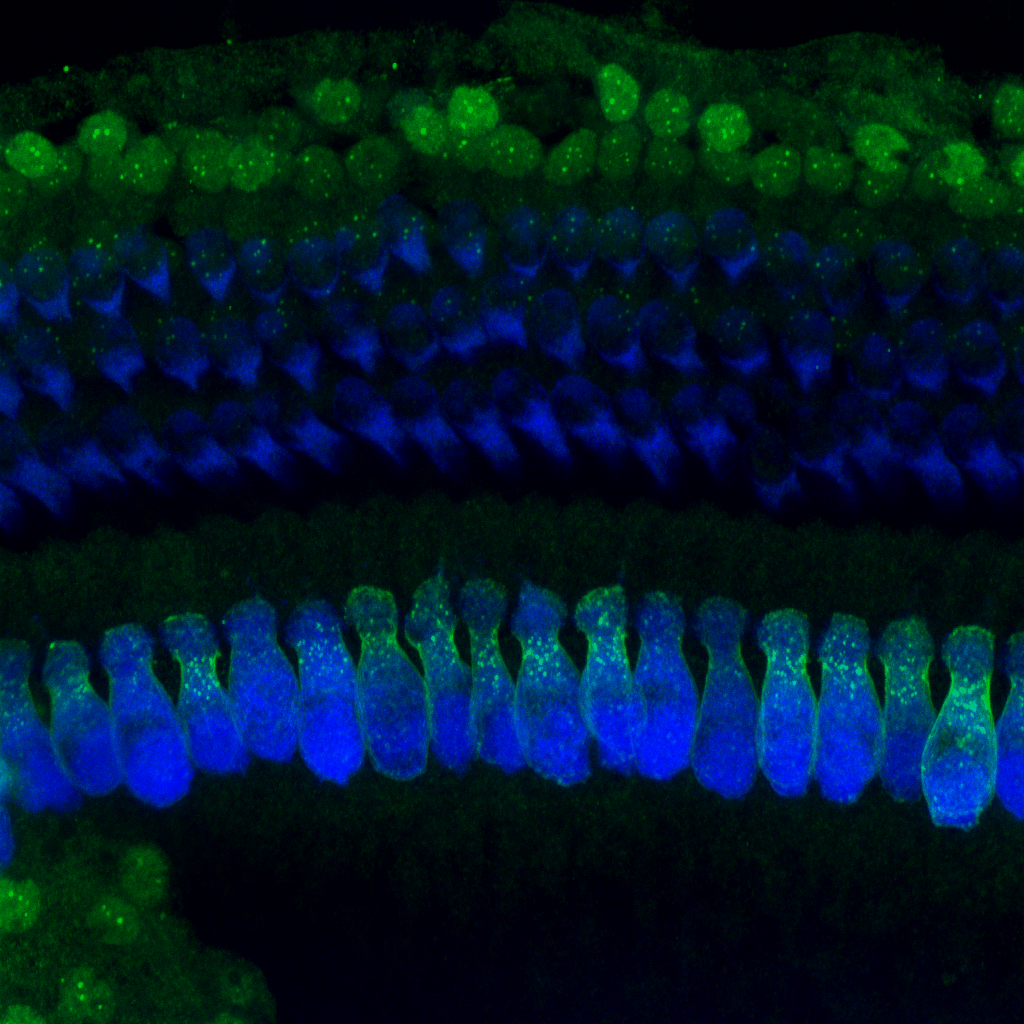

Supplement: Supplementary file 7 — Source Data for Figure 1 [file EMBJ-42-e112118-s002.zip › Figure 1/Figure 1B.tif (RGB).Tif]

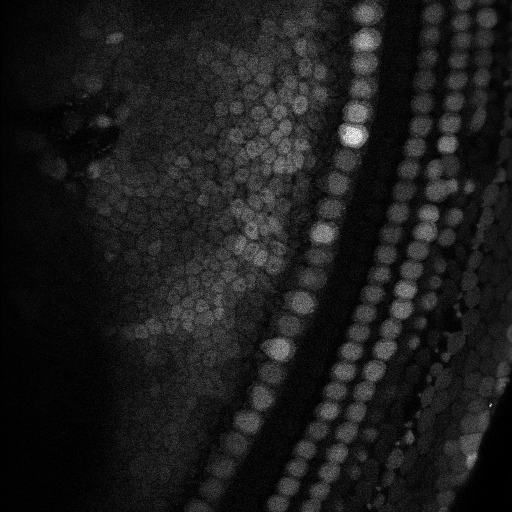

Supplement: Supplementary file 8 — Source Data for Figure 2 [file EMBJ-42-e112118-s014.zip › Figure 2/Figure 2F_Bottom panel.tif]

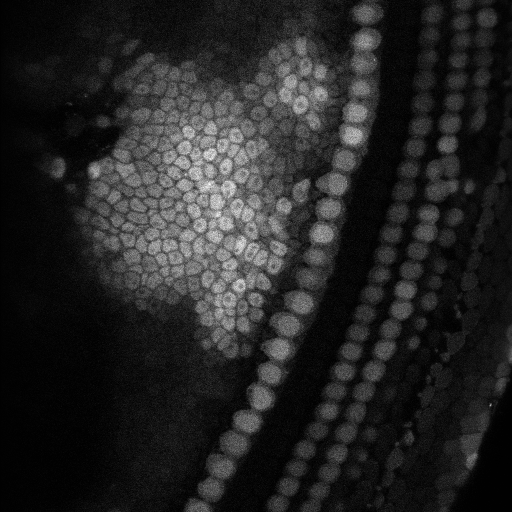

Supplement: Supplementary file 8 — Source Data for Figure 2 [file EMBJ-42-e112118-s014.zip › Figure 2/Figure 2F_Middle panel.tif]

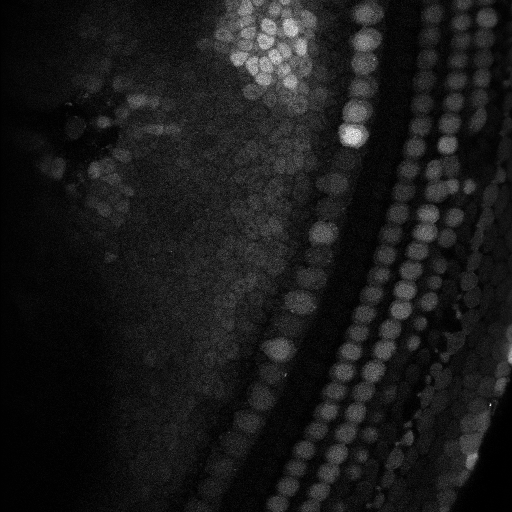

Supplement: Supplementary file 8 — Source Data for Figure 2 [file EMBJ-42-e112118-s014.zip › Figure 2/Figure 2F_Top panel.tif]

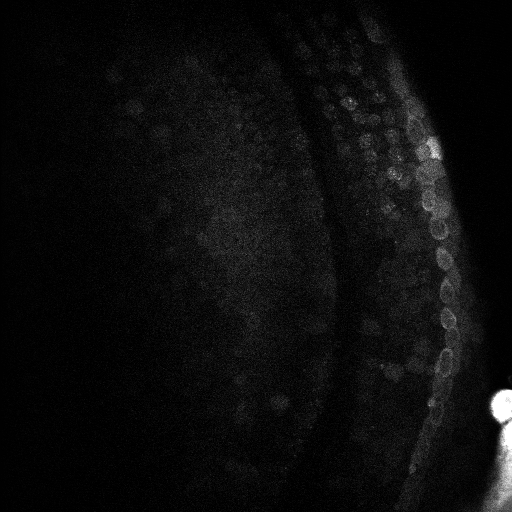

Supplement: Supplementary file 8 — Source Data for Figure 2 [file EMBJ-42-e112118-s014.zip › Figure 2/Figure 2G_Bottom panel.tif]

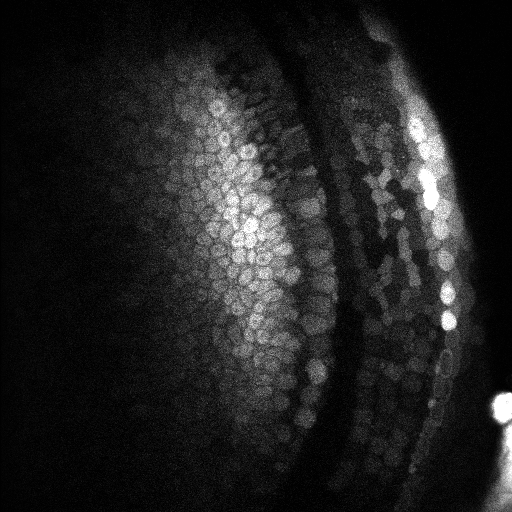

Supplement: Supplementary file 8 — Source Data for Figure 2 [file EMBJ-42-e112118-s014.zip › Figure 2/Figure 2G_Middle panel.tif]

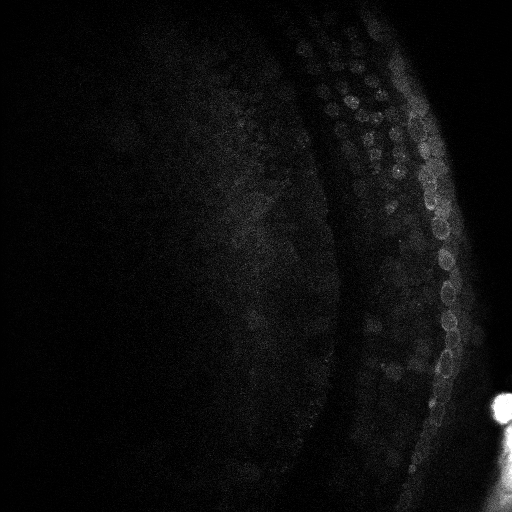

Supplement: Supplementary file 8 — Source Data for Figure 2 [file EMBJ-42-e112118-s014.zip › Figure 2/Figure 2G_Top panel.tif]

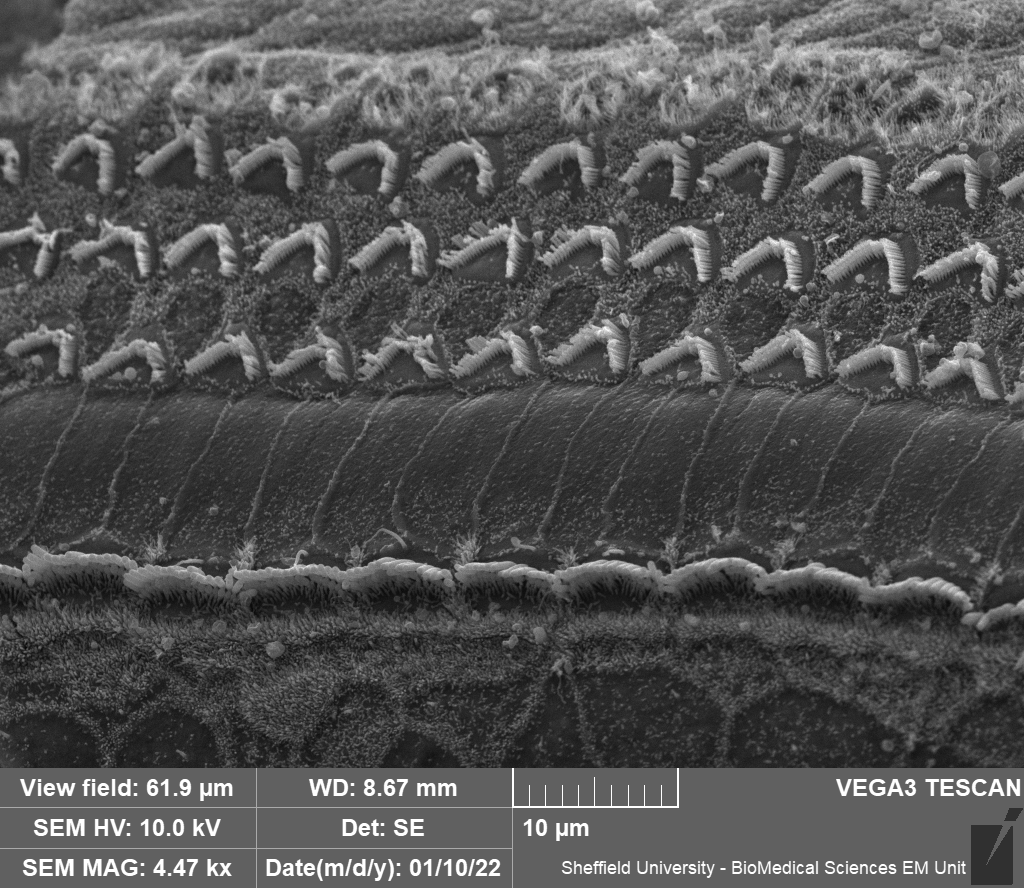

Supplement: Supplementary file 11 — Source Data for Figure 5 [file EMBJ-42-e112118-s005.zip › Figure 5/Figure 5A_P11_Left & Right Panels.tif]

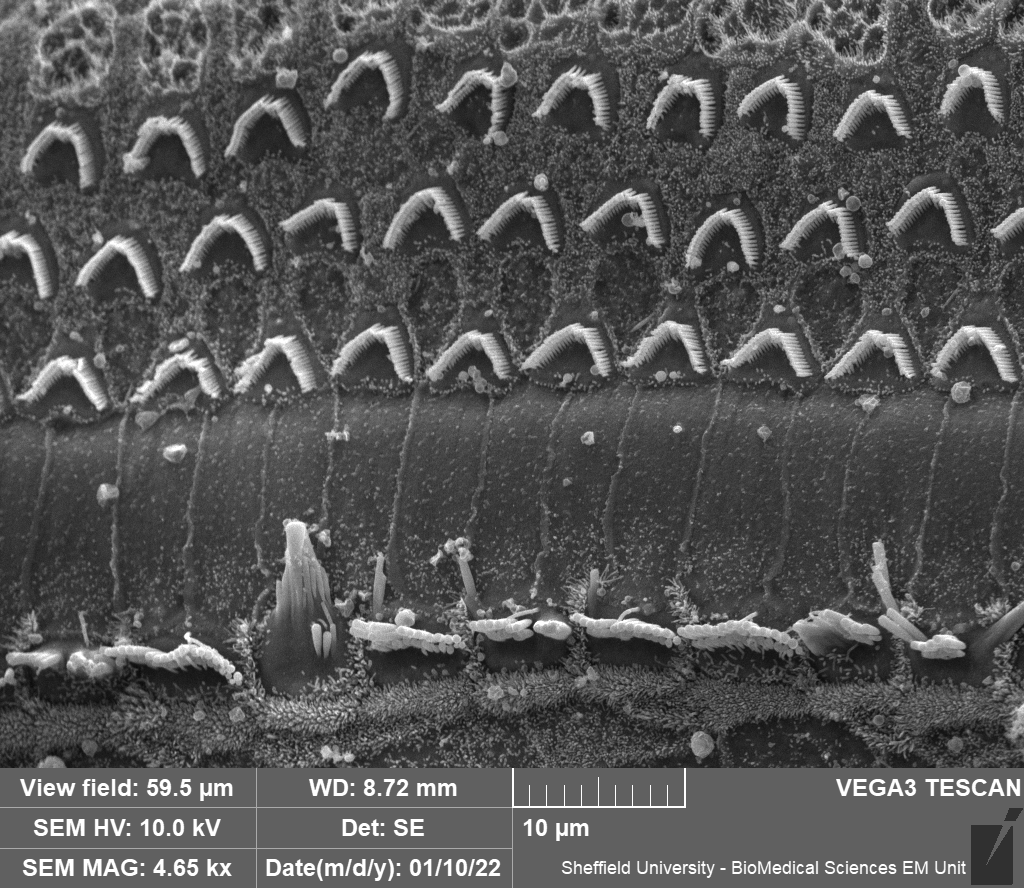

Supplement: Supplementary file 11 — Source Data for Figure 5 [file EMBJ-42-e112118-s005.zip › Figure 5/Figure 5B_P11_Left Panel.tif]

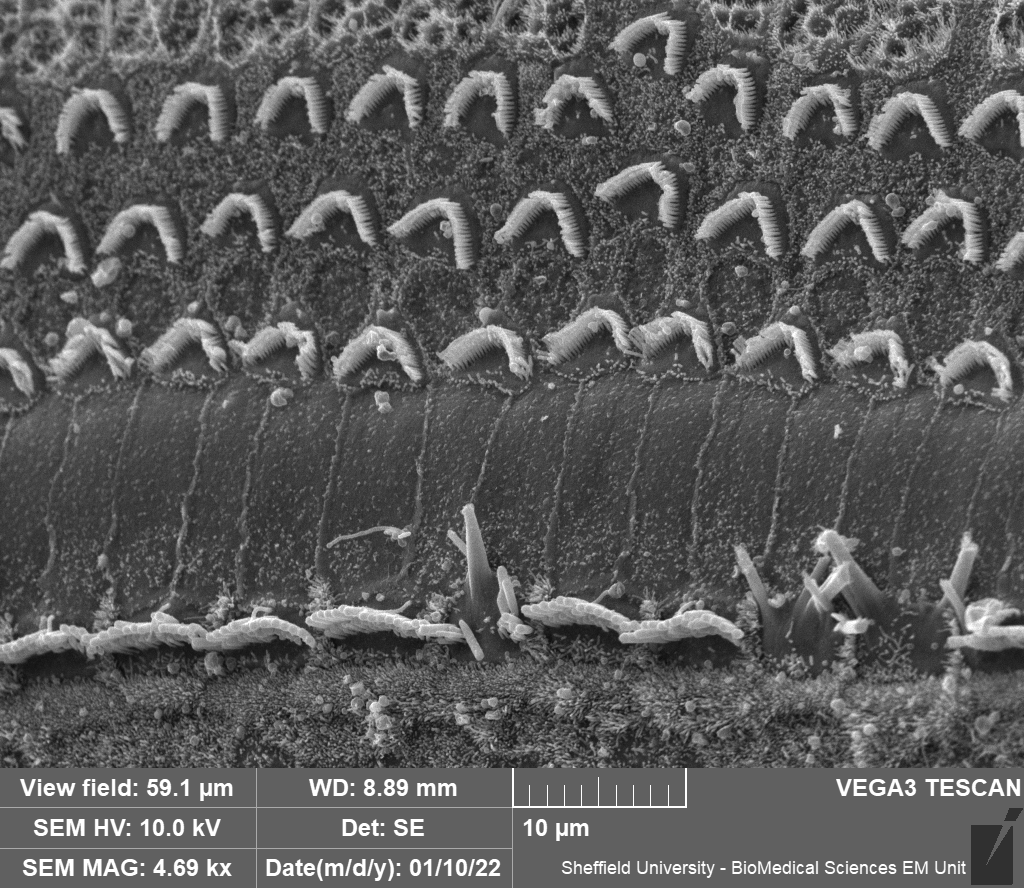

Supplement: Supplementary file 11 — Source Data for Figure 5 [file EMBJ-42-e112118-s005.zip › Figure 5/Figure 5B_P11_Right Panel.tif]

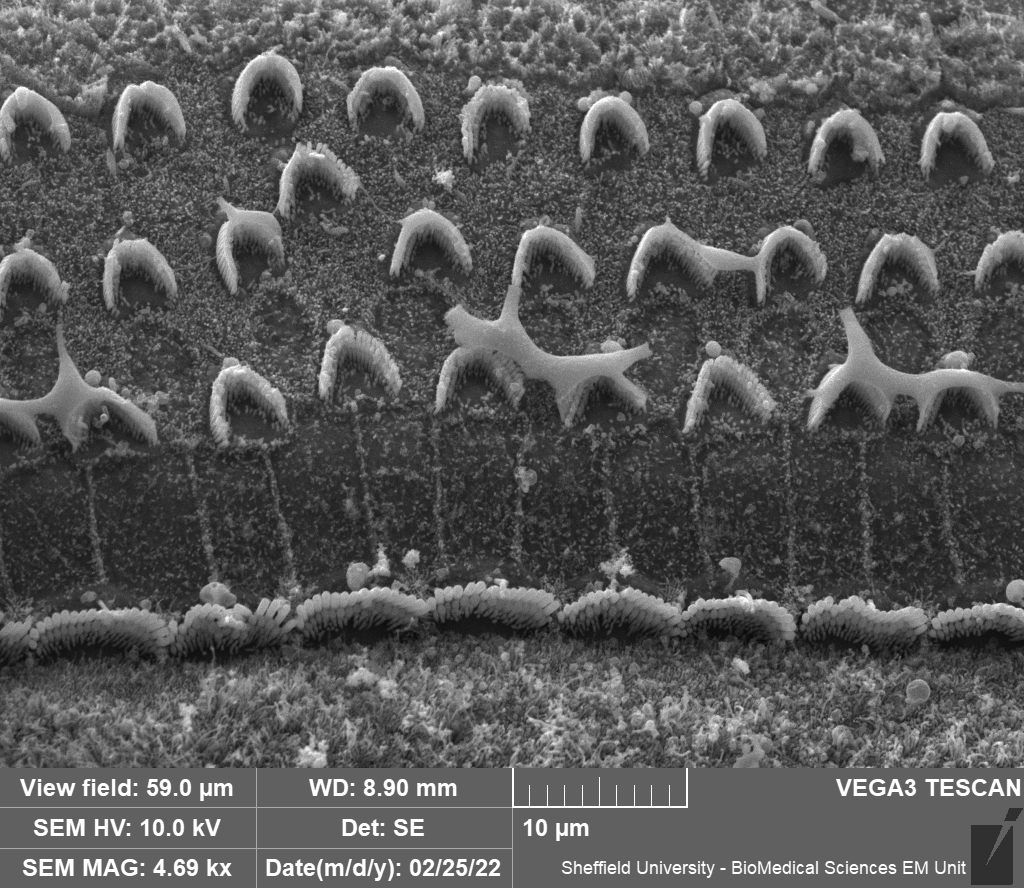

Supplement: Supplementary file 11 — Source Data for Figure 5 [file EMBJ-42-e112118-s005.zip › Figure 5/Figure 5B_P8_Left Panel.tif]

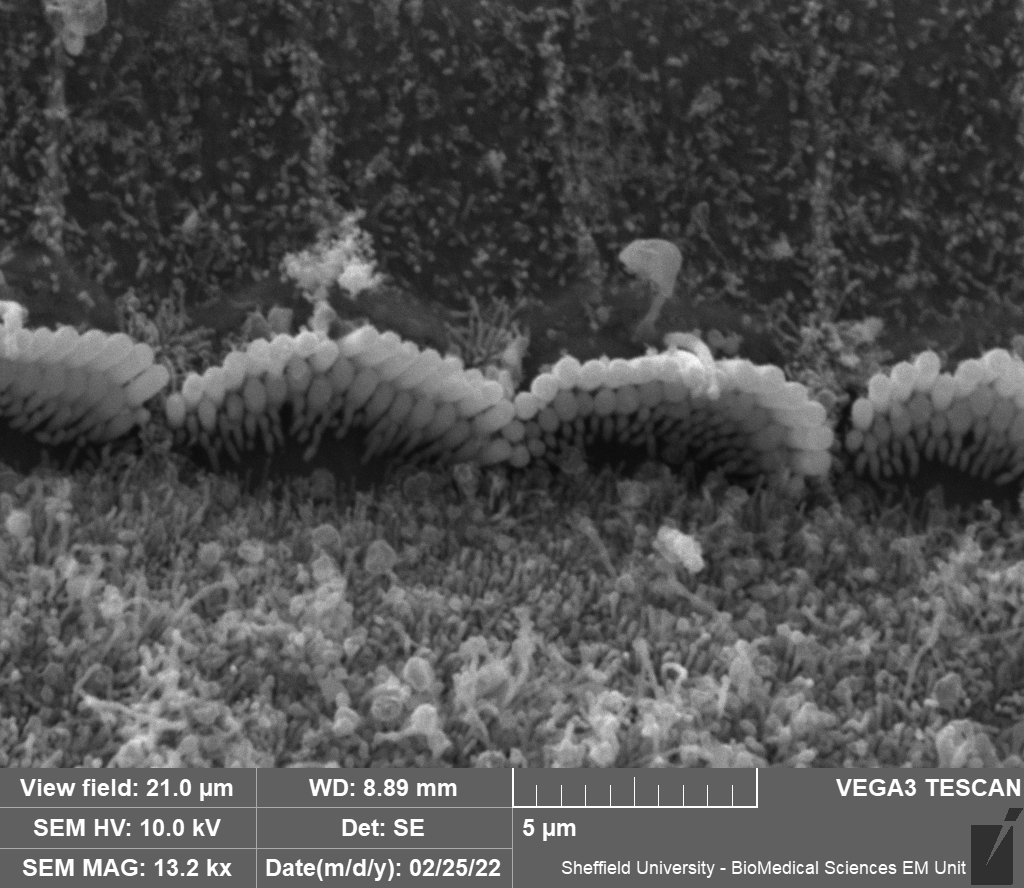

Supplement: Supplementary file 11 — Source Data for Figure 5 [file EMBJ-42-e112118-s005.zip › Figure 5/Figure 5B_P8_Right Panel.tif]

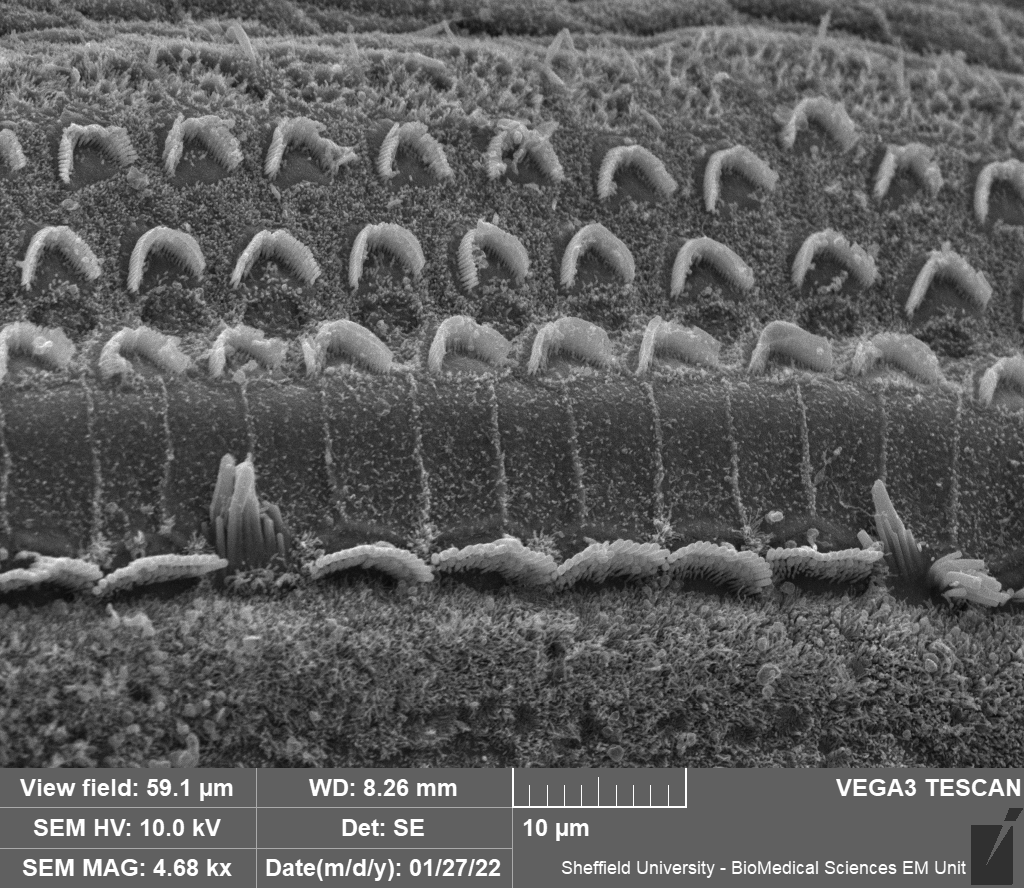

Supplement: Supplementary file 11 — Source Data for Figure 5 [file EMBJ-42-e112118-s005.zip › Figure 5/Figure 5B_P9_Left & Right Panels.tif]

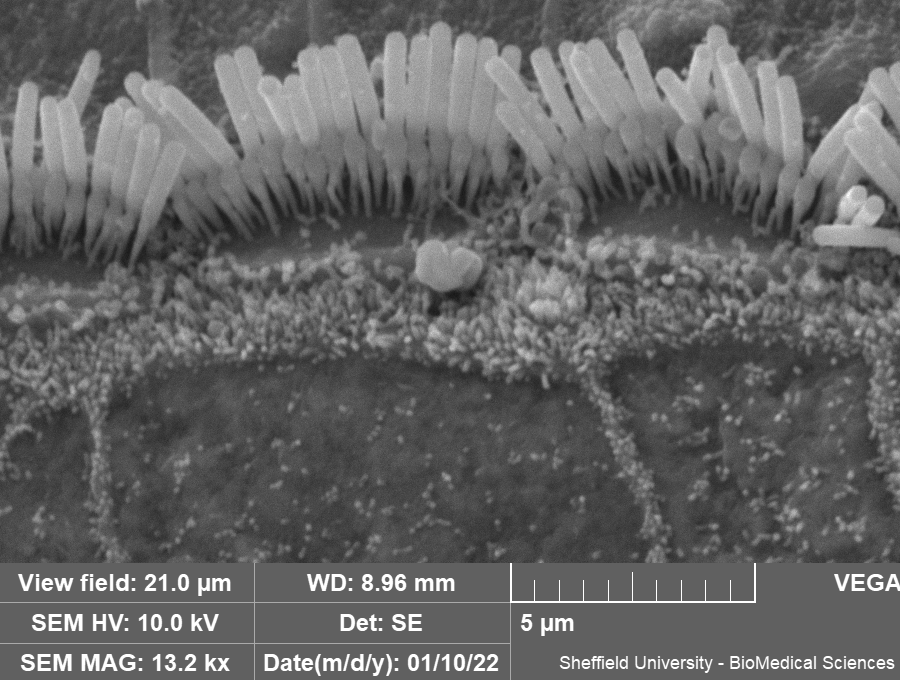

Supplement: Supplementary file 11 — Source Data for Figure 5 [file EMBJ-42-e112118-s005.zip › Figure 5/Figure 5C_Bottom Panel.tif]

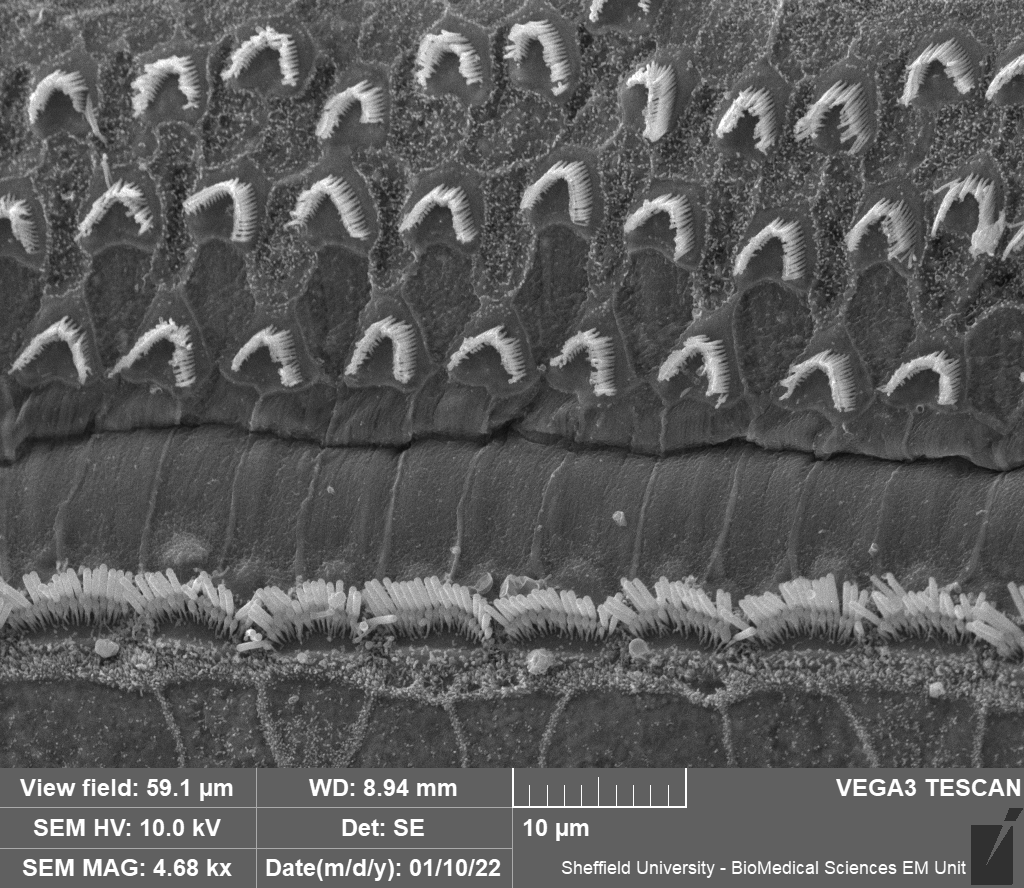

Supplement: Supplementary file 11 — Source Data for Figure 5 [file EMBJ-42-e112118-s005.zip › Figure 5/Figure 5C_Top Panel.tif]

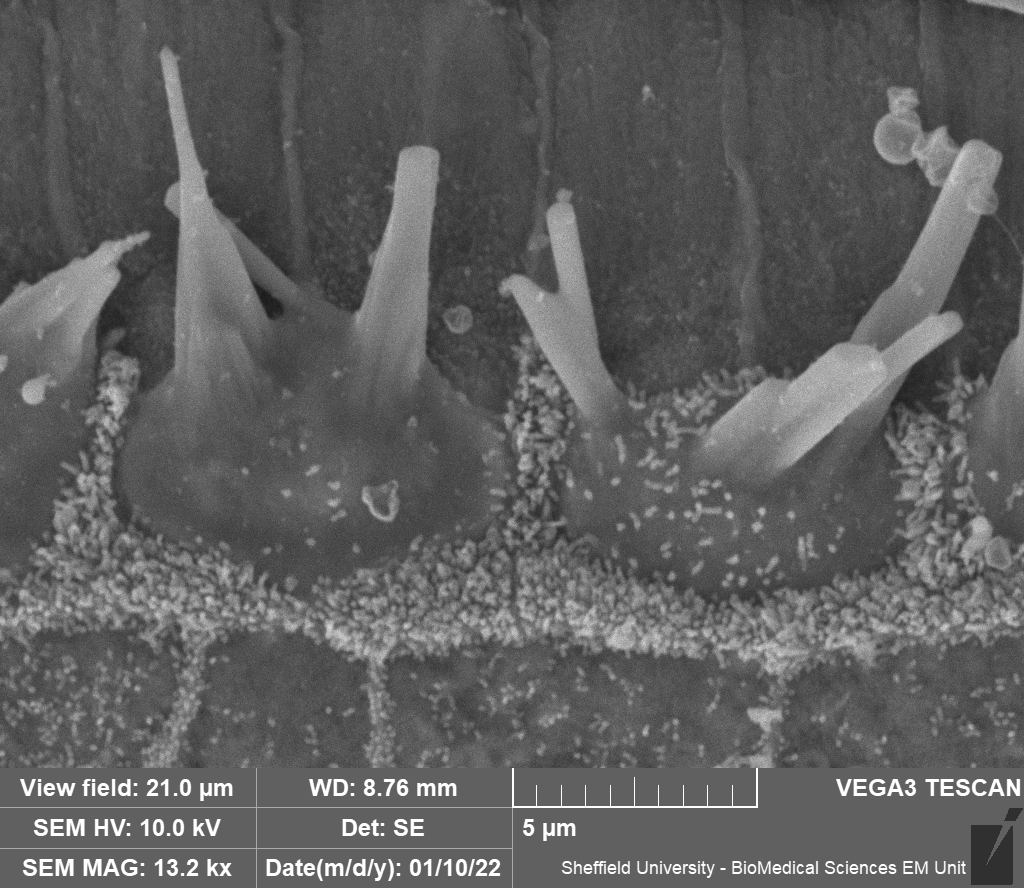

Supplement: Supplementary file 11 — Source Data for Figure 5 [file EMBJ-42-e112118-s005.zip › Figure 5/Figure 5D_Bottom Panel.tif]

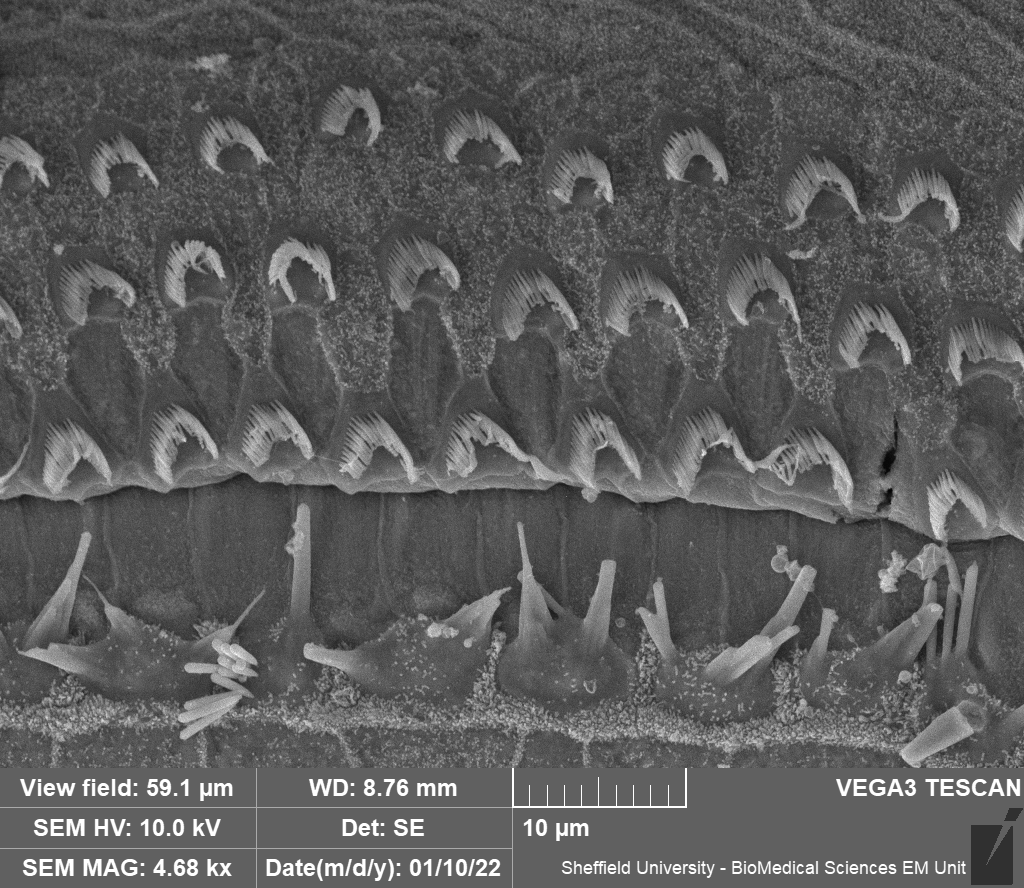

Supplement: Supplementary file 11 — Source Data for Figure 5 [file EMBJ-42-e112118-s005.zip › Figure 5/Figure 5D_Top Panel.tif]

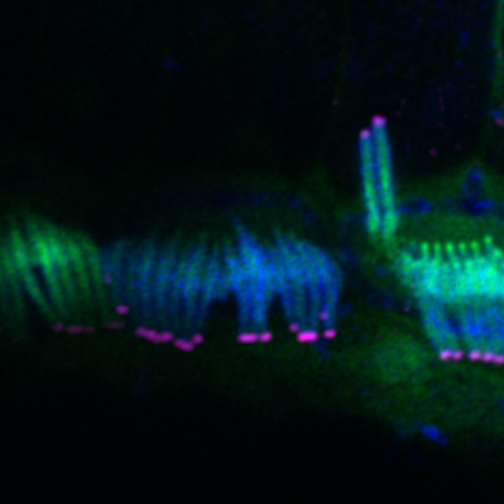

Supplement: Supplementary file 12 — Source Data for Figure 6 [file EMBJ-42-e112118-s008.zip › Figure 6/Figure 6A_P11.tif]

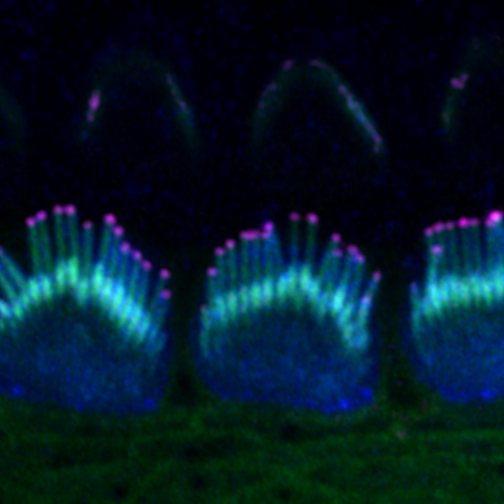

Supplement: Supplementary file 12 — Source Data for Figure 6 [file EMBJ-42-e112118-s008.zip › Figure 6/Figure 6A_P6.tif]

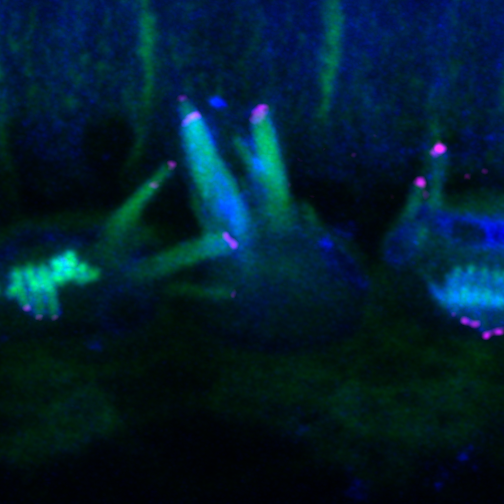

Supplement: Supplementary file 12 — Source Data for Figure 6 [file EMBJ-42-e112118-s008.zip › Figure 6/Figure 6B_P11.tif]

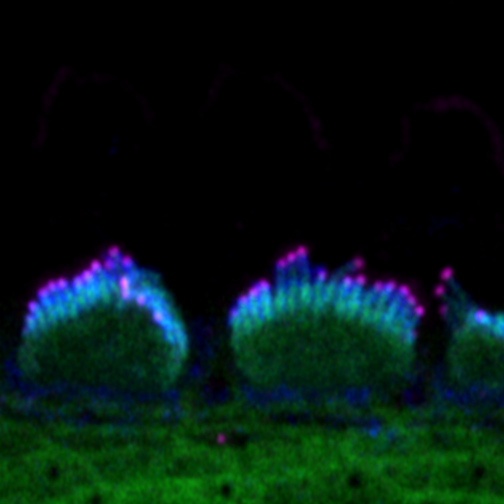

Supplement: Supplementary file 12 — Source Data for Figure 6 [file EMBJ-42-e112118-s008.zip › Figure 6/Figure 6B_P6.tif]

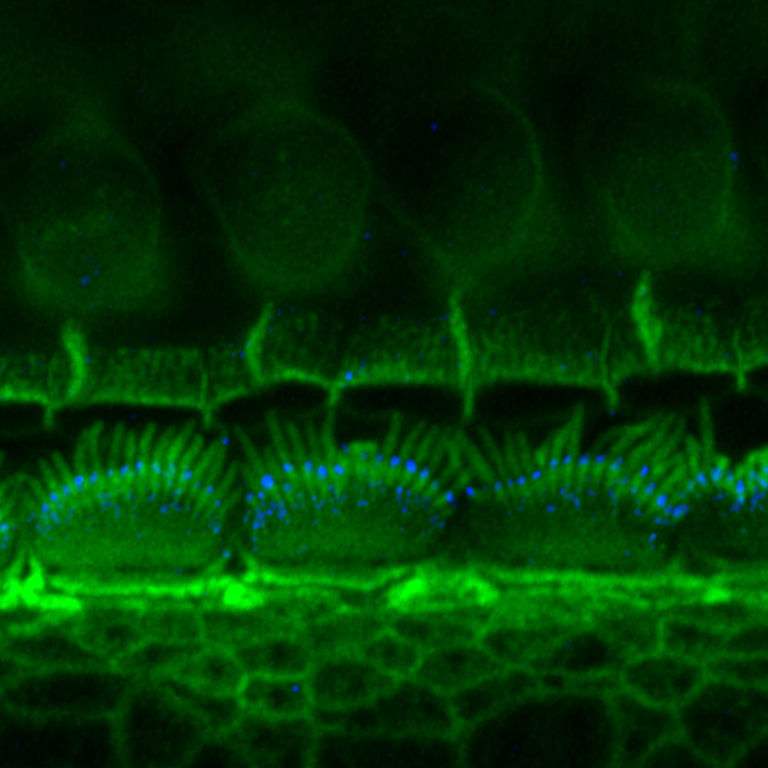

Supplement: Supplementary file 12 — Source Data for Figure 6 [file EMBJ-42-e112118-s008.zip › Figure 6/Figure 6C_P11_MYOSIN XV.tif]

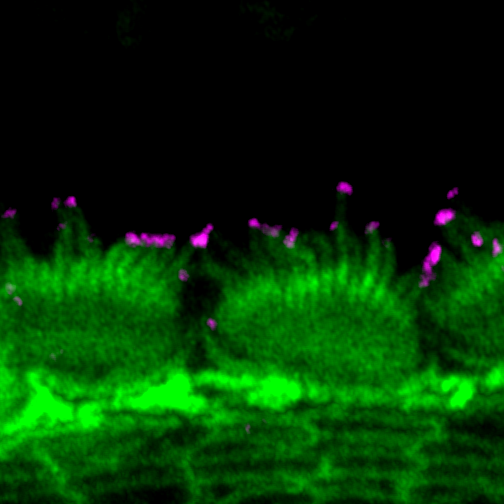

Supplement: Supplementary file 12 — Source Data for Figure 6 [file EMBJ-42-e112118-s008.zip › Figure 6/Figure 6C_P11_WHIRLIN.tif]

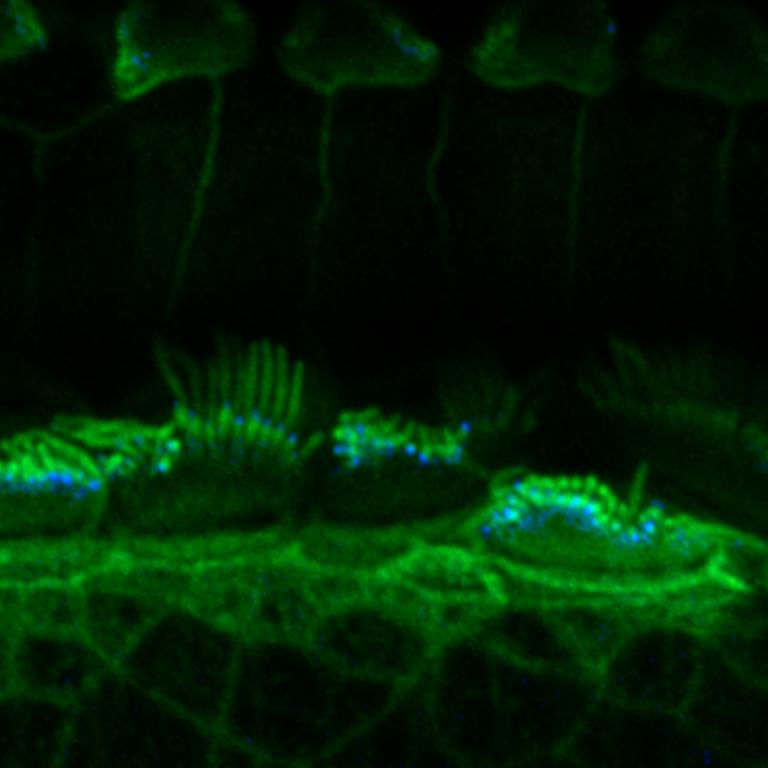

Supplement: Supplementary file 12 — Source Data for Figure 6 [file EMBJ-42-e112118-s008.zip › Figure 6/Figure 6D_P11_MYOSIN XV.tif]

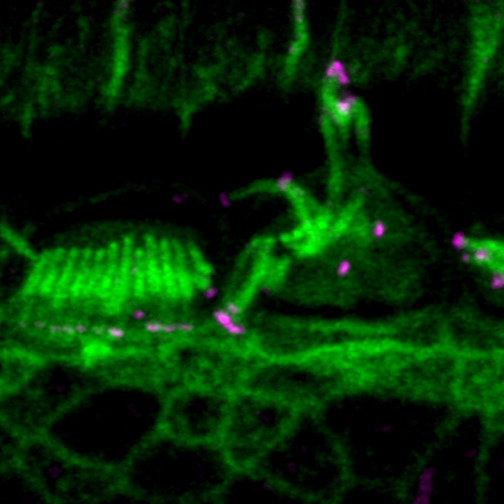

Supplement: Supplementary file 12 — Source Data for Figure 6 [file EMBJ-42-e112118-s008.zip › Figure 6/Figure 6D_P11_WHIRLIN.tif]

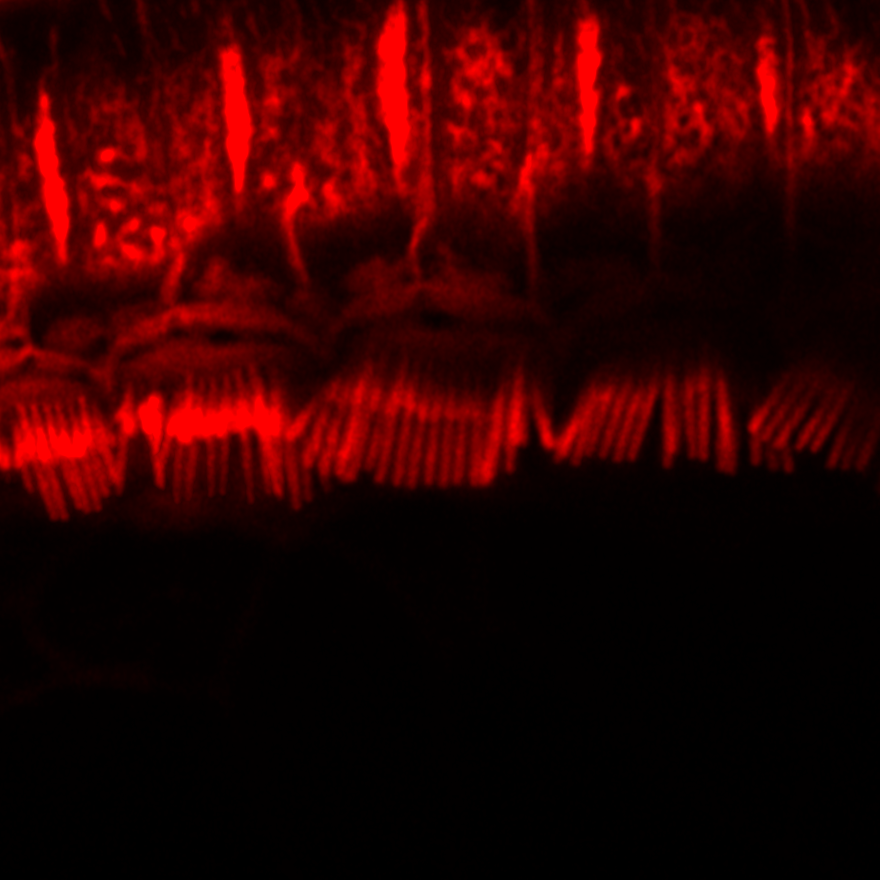

Supplement: Supplementary file 13 — Source Data for Figure 7 [file EMBJ-42-e112118-s006.zip › Figure 7/Figure 7H.tif]

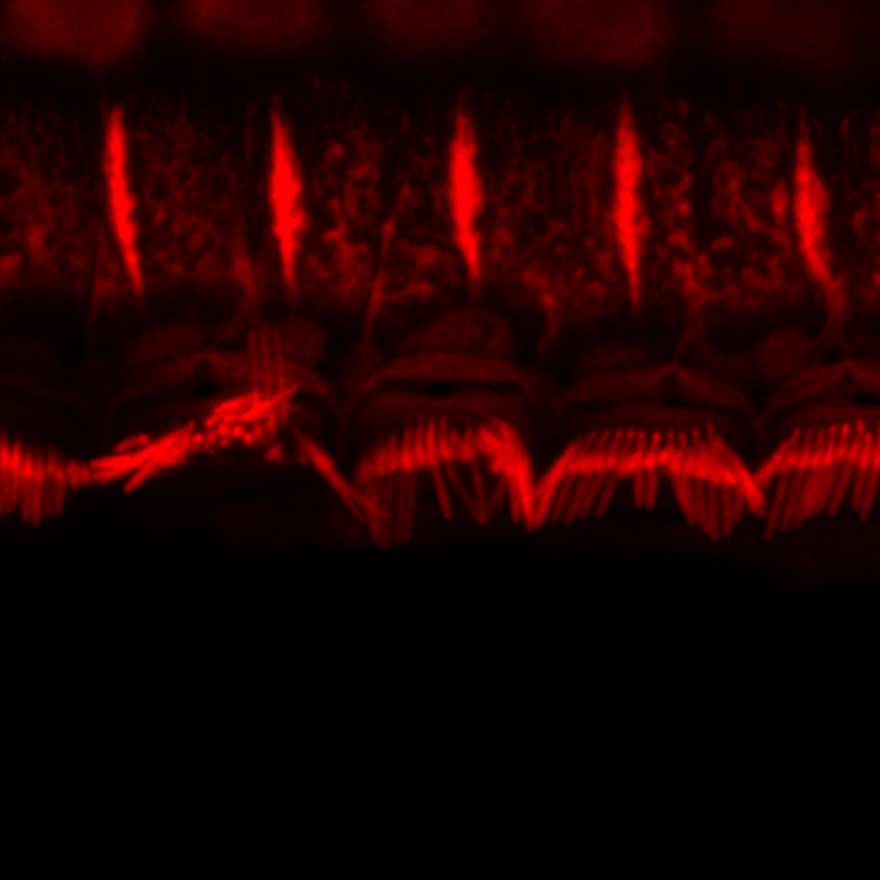

Supplement: Supplementary file 13 — Source Data for Figure 7 [file EMBJ-42-e112118-s006.zip › Figure 7/Figure 7I.tif]

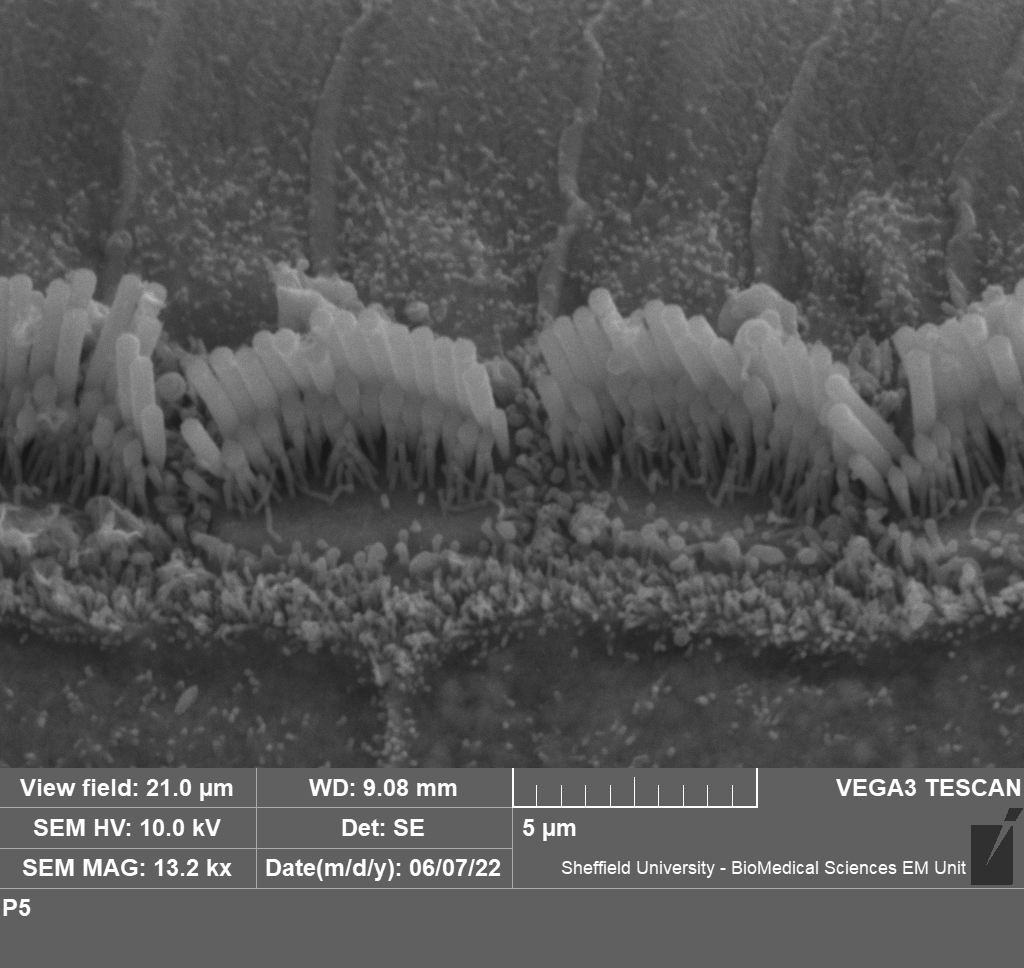

Supplement: Supplementary file 13 — Source Data for Figure 7 [file EMBJ-42-e112118-s006.zip › Figure 7/Figure 7J.tif]

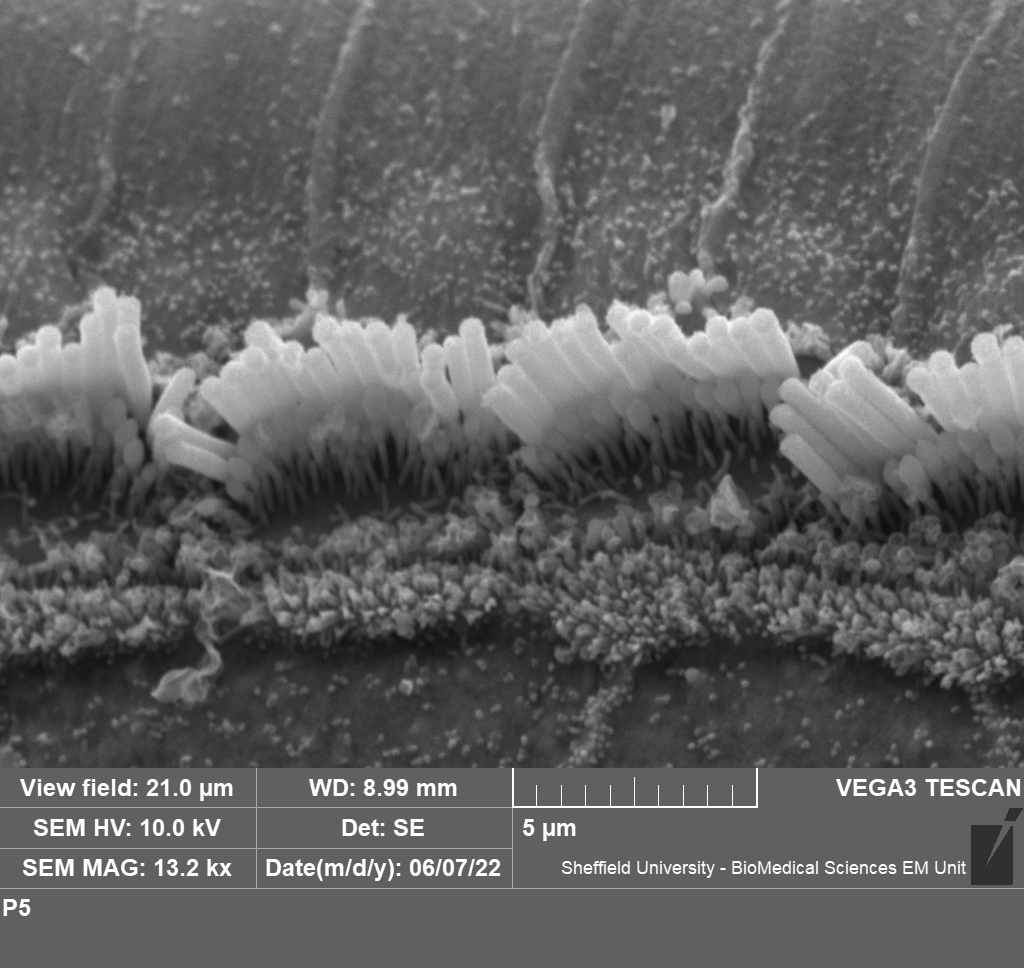

Supplement: Supplementary file 13 — Source Data for Figure 7 [file EMBJ-42-e112118-s006.zip › Figure 7/Figure 7K.tif]

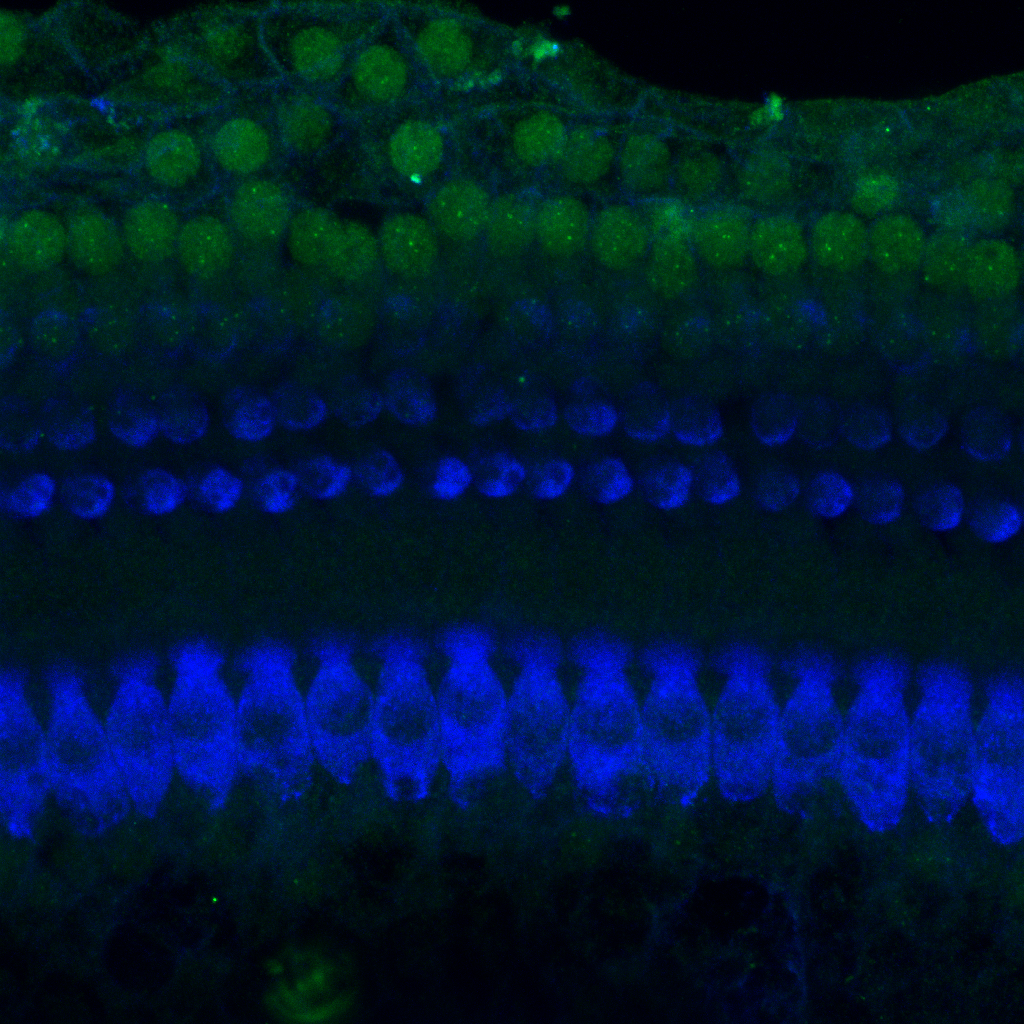

Supplement: Supplementary file 13 — Source Data for Figure 7 [file EMBJ-42-e112118-s006.zip › Figure 7/Figure S7A_P14.tif]

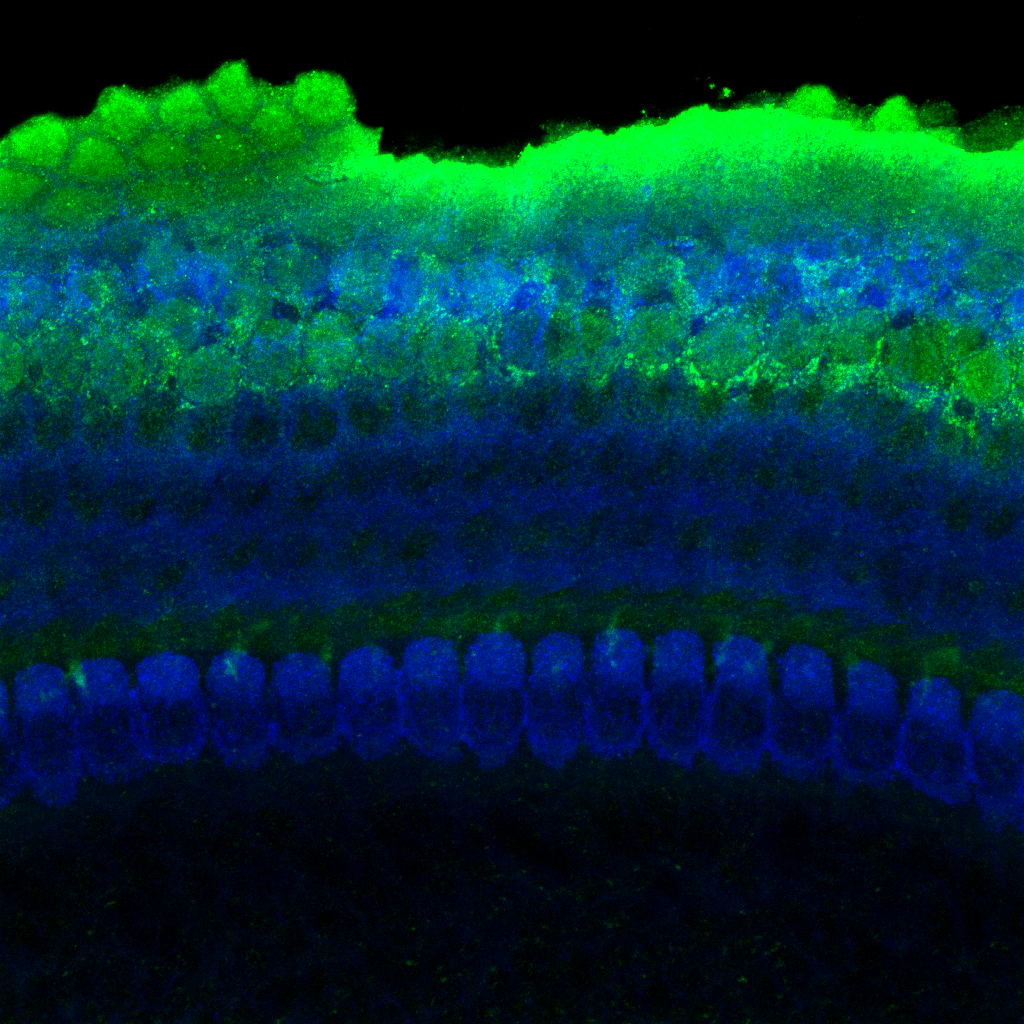

Supplement: Supplementary file 13 — Source Data for Figure 7 [file EMBJ-42-e112118-s006.zip › Figure 7/Figure S7A_P5.tif]

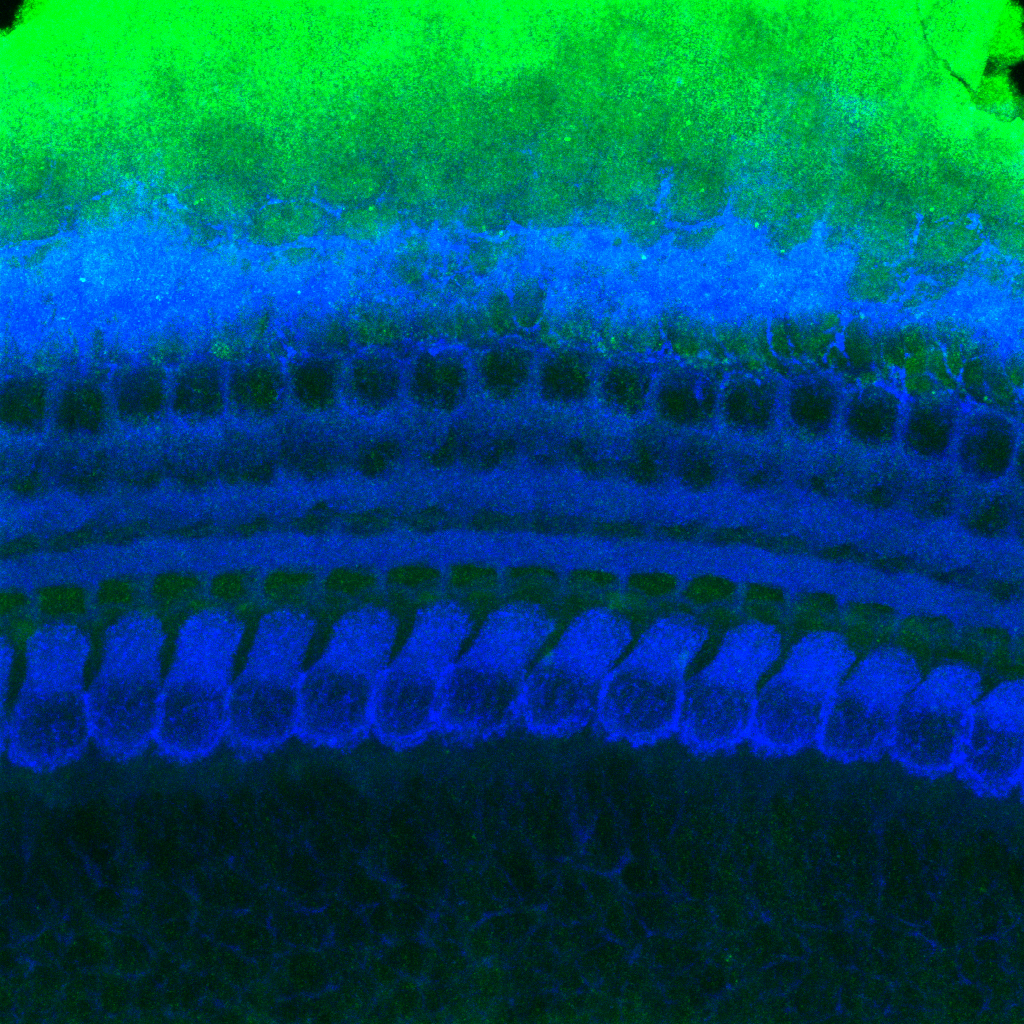

Supplement: Supplementary file 13 — Source Data for Figure 7 [file EMBJ-42-e112118-s006.zip › Figure 7/Figure S7A_P7.tif]

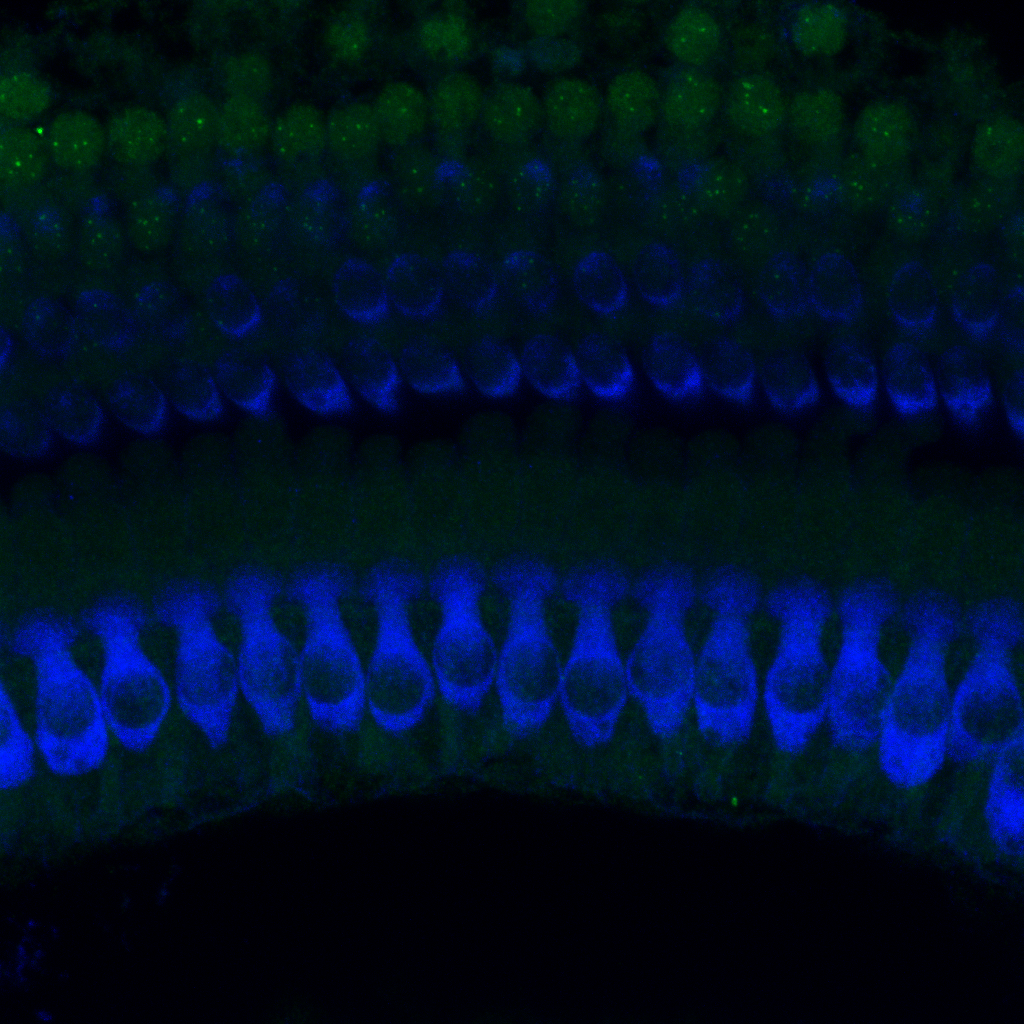

Supplement: Supplementary file 13 — Source Data for Figure 7 [file EMBJ-42-e112118-s006.zip › Figure 7/Figure S7B_P14.tif]

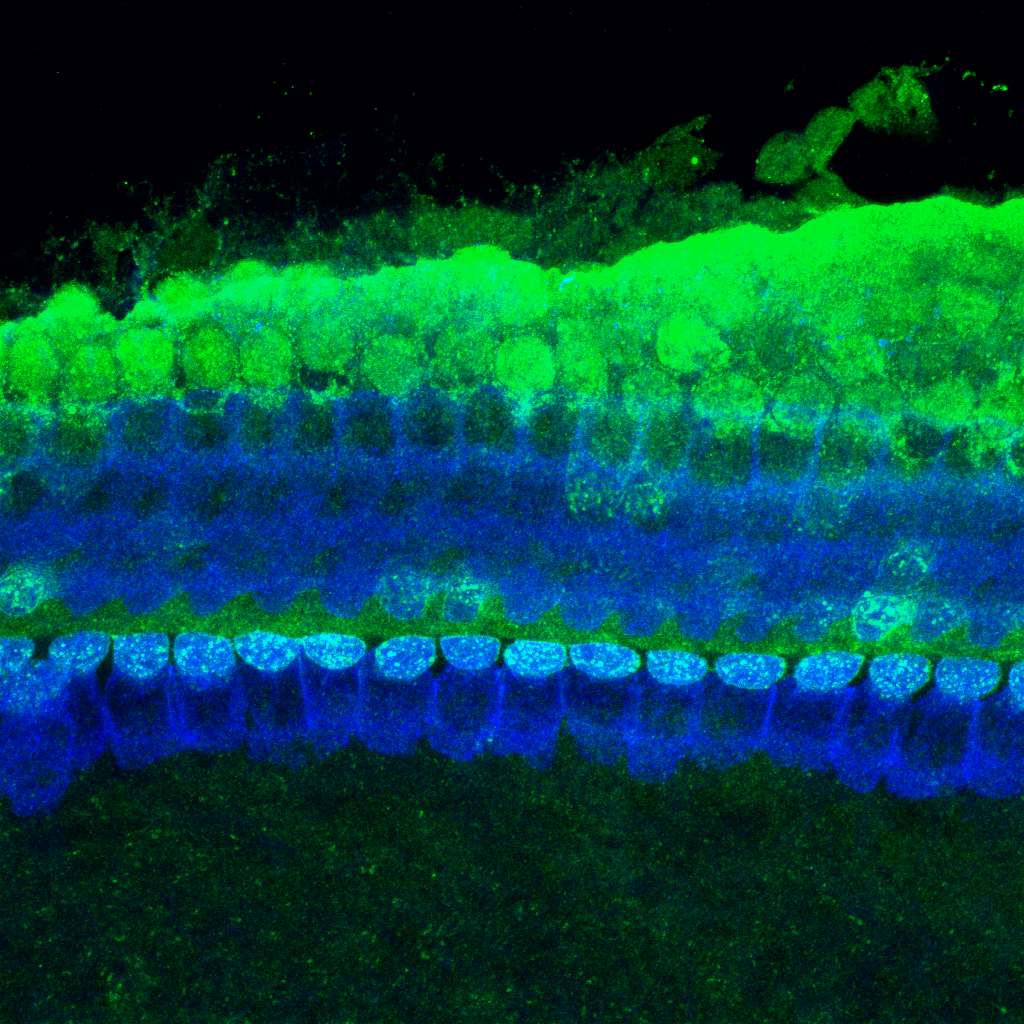

Supplement: Supplementary file 13 — Source Data for Figure 7 [file EMBJ-42-e112118-s006.zip › Figure 7/Figure S7B_P5.tif]

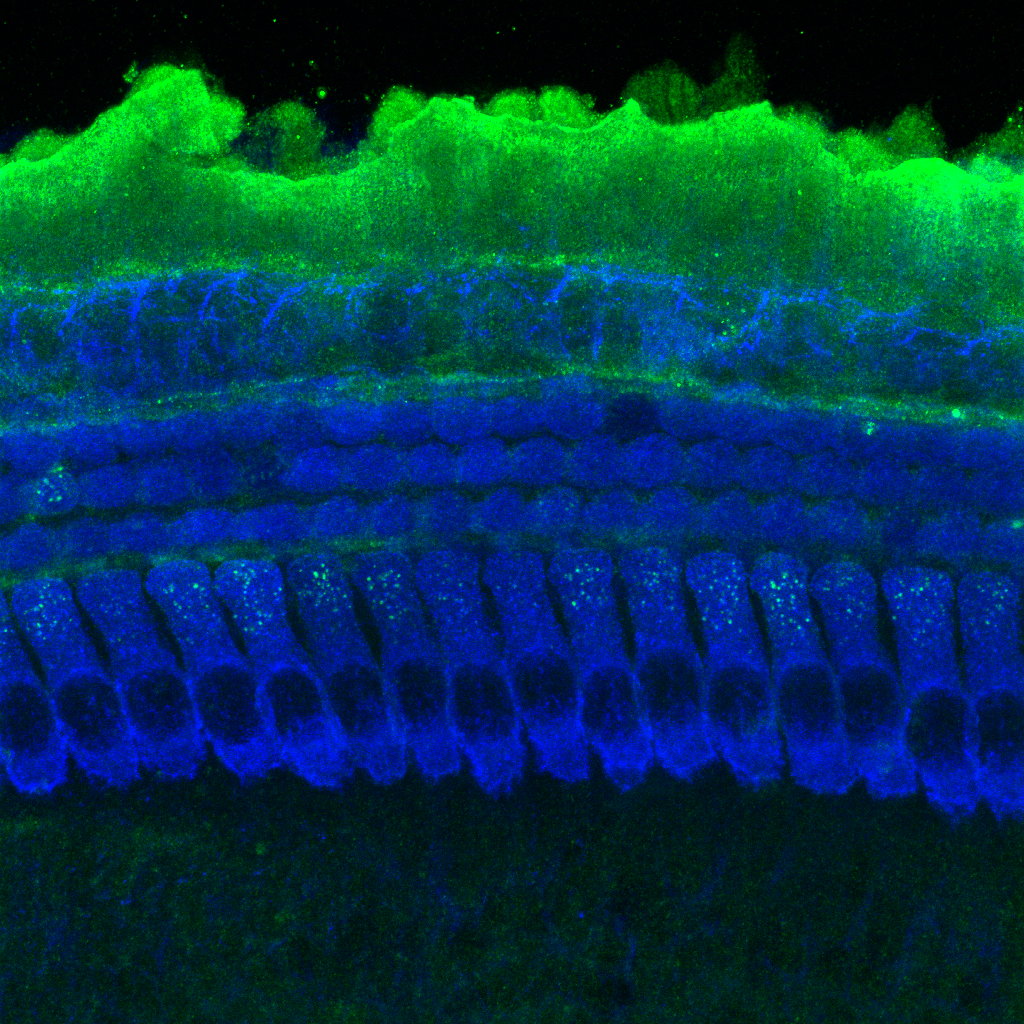

Supplement: Supplementary file 13 — Source Data for Figure 7 [file EMBJ-42-e112118-s006.zip › Figure 7/Figure S7B_P7.tif]
