# Supplementary material for: A critical period of prehearing spontaneous Ca2+ spiking is required for hair‐bundle maintenance in inner hair cells
Source: EMBO J. 2023 Jan 3;42(4):e112118. doi: 10.15252/embj.2022112118 (PMC9929643; doi:10.15252/embj.2022112118)
Supplement: Supplementary file 6 — PDF+ [file EMBJ-42-e112118-s009.pdf]

Article

SOURCE DATA | TRANSPARENT PROCESS | OPEN ACCESS

THE EMBO JOURNAL

A critical period of prehearing spontaneous Ca^{2+} spiking is required for hair-bundle maintenance in inner hair cells

Adam J Carlton[1], Jing-Yi Jeng[1], Fiorella C Grandi[2], Francesca De Faveri[1], Federico Ceriani[1], Lara De Tomasi[1], Anna Underhill[1], Stuart L Johnson[1,3], Kevin P Legan[4], Corné J Kros[4], Guy P Richardson[4], Mirna Mustapha[1,3] & Walter Marcotti[1,3,*]

Abstract

Sensory-independent Ca^{2+} spiking regulates the development of mammalian sensory systems. In the immature cochlea, inner hair cells (IHCs) fire spontaneous Ca^{2+} action potentials (APs) that are generated either intrinsically or by intercellular Ca^{2+} waves in the nonsensory cells. The extent to which either or both of these Ca^{2+} signalling mechansims are required for IHC maturation is unknown. We find that intrinsic Ca^{2+} APs in IHCs, but not those elicited by Ca^{2+} waves, regulate the maturation and maintenance of the stereociliary hair bundles. Using a mouse model in which the potassium channel Kir2.1 is reversibly overexpressed in IHCs (*Kir2.1*-OE), we find that IHC membrane hyperpolarization prevents IHCs from generating intrinsic Ca^{2+} APs but not APs induced by Ca^{2+} waves. Absence of intrinsic Ca^{2+} APs leads to the loss of mechanoelectrical transduction in IHCs prior to hearing onset due to progressive loss or fusion of stereocilia. RNA-sequencing data show that pathways involved in morphogenesis, actin filament-based processes, and Rho-GTPase signaling are upregulated in *Kir2.1*-OE mice. By manipulating *in vivo* expression of Kir2.1 channels, we identify a "critical time period" during which intrinsic Ca^{2+} APs in IHCs regulate hair-bundle function.

Keywords calcium waves; development; hair cell; mechanoelectrical transduction; spontaneous action potentials
Subject Category Neuroscience
DOI 10.15252/embj.2022112118 | Received 16 July 2022 | Revised 22 November 2022 | Accepted 28 November 2022 | Published online 3 January 2023
The EMBO Journal (2023) 42: e112118

Introduction

Inner hair cells (IHCs) are the primary sensory receptors of the adult mammalian cochlea and relay acoustic information onto type I spiral ganglion afferent neurons via the graded release of glutamate from their specialized ribbon synapses (Fuchs, 2005; Moser *et al*, 2020). Before hearing onset, however, which in most altricial rodents occurs at around postnatal day 12 (P12) (Mikaelian & Ruben, 1964; Ehret, 1983; Romand, 1983), IHCs exhibit patterned action potential activity that is elicited spontaneously in the absence of sound-induced stimulation by the activation of $Ca_V1.3$ Ca^{2+} channels (Marcotti *et al*, 2003a; Tritsch *et al*, 2010; Johnson *et al*, 2011). This activity has been shown to drive the bursting-like firing pattern along the neural pathway of the immature auditory system (Lippe, 1994; Jones *et al*, 2007; Sonntag *et al*, 2009; Tritsch *et al*, 2010). As with other sensory systems (Katz & Shatz, 1996; Stellwagen & Shatz, 2002; Moody & Bosma, 2005; Blankenship & Feller, 2010), patterned peripheral firing activity was identified as being critical for the refinement of neural circuits in the brain (Clause *et al*, 2014, 2017; Müller *et al*, 2019; Maul *et al*, 2022). Additionally, Ca^{2+}-dependent APs in IHCs have been shown to instruct the normal functional differentiation of the IHCs themselves (Johnson *et al*, 2007, 2013), most likely via regulating gene expression (Dolmetsch *et al*, 1997). However, due to the complex extracellular modulation of the intrinsic Ca^{2+} action potentials in developing IHCs, the exact role of this activity is largely unknown.

Spontaneous intrinsic Ca^{2+} action potentials first appear in the IHCs of the mouse cochlea at late embryonic stages (Marcotti *et al*, 2003a), and their frequency and pattern are controlled by the transiently expressed small-conductance Ca^{2+}-activated K^+ current I_{SK2} (Marcotti *et al*, 2004) and the inward rectifier K^+ current I_{K1} (Marcotti *et al*, 1999). The frequency and pattern of the electrical activity in IHCs are also extrinsically evoked and modulated by the spontaneous release of ATP from the neighboring nonsensory cells (Tritsch *et al*, 2010; Wang *et al*, 2015; Johnson *et al*, 2017). This complex regulation makes it difficult to identify and separate the specific functional roles of the intrinsic and externally driven Ca^{2+}-dependent AP activity in IHCs.

In this study, we used a mouse model in which the inward rectifier K^+ channel Kir2.1 (Yu *et al*, 2004) was selectively overexpressed

1 School of Biosciences, University of Sheffield, Sheffield, UK
2 Gladstone Institute of Neurological Disease, San Francisco, CA, USA
3 Neuroscience Institute, University of Sheffield, Sheffield, UK
4 School of Life Sciences, University of Sussex, Falmer, Brighton, UK
*Corresponding author. Tel: +44 114 2221098; E-mail: w.marcotti@sheffield.ac.uk

© 2023 The Authors. Published under the terms of the CC BY 4.0 license.

in vivo in the IHCs under the control of doxycycline (DOX), lowering their membrane potential and preventing them from firing the intrinsic spontaneous Ca^{2+} action potentials. These "silent" IHCs, however, retained their ability to respond with AP activity to extrinsic modulation by the ATP-induced signaling from the nonsensory cochlear cells. Our results show that prehearing IHCs require spontaneous intrinsic Ca^{2+} firing to maintain the normal morphological and biophysical characteristics of the mechanoelectrical transducer apparatus for a period of time in the second postnatal week, before the onset of hearing. We also found several key genes that are upregulated in the absence of the intrinsic Ca^{2+} action potential activity in IHCs, several of which are involved in pathways related to maintaining cytoskeletal homeostasis.

Results

Overexpression of *Kir2.1* (*Kir2.1*-OE) in cochlear IHCs *in vivo* prevents spontaneous firing activity

The role of spontaneous Ca^{2+} action potential activity in IHCs, which are spikes generated intrinsically as opposed to those induced by Ca^{2+} waves originating in the nonsensory cells, was investigated by conditionally overexpressing the inwardly rectifying K^+ channel Kir2.1 (*Kcnj2*) in the IHCs, thereby hyperpolarizing their resting membrane potential.

DOX-induced overexpression of Kir2.1 channels in the IHCs was evident from the presence of Kir2.1 immunofluorescence in the basolateral membrane of P6 (Fig EV1B) and P11 *Kir2.1*-OE mice (*Otof*$^{tTA+/-}$; *Kir2.1*$^{+/-}$: Fig 1B), but not in age-matched littermate control mice that were also exposed to DOX (*Otof*$^{tTA+/-}$; *Kir2.1*$^{+/+}$: P6, Fig EV1A; P9-P11, Fig 1A). OHCs and nonsensory cells surrounding the hair cells showed no or very little overexpression of Kir2.1 (Appendix Fig S1), indicating specificity of the *Otof* promoter for targeting the IHCs. Prehearing IHCs overexpressing Kir2.1 showed a significantly larger inward K^+ current compared with control cells but normal outward K^+ currents (Fig 1C–G, P9-P11). The larger inward K^+ current in the IHCs from ***Kir2.1*-OE** led to a hyperpolarized shift of the resting membrane potential (V_m) of the IHCs of about 10 mV compared with control cells (Fig 1H). The slope conductance around the respective resting V_m values was also significantly increased in IHCs from *Kir2.1-OE* mice compared with control littermates (Fig 1I). The overexpression of Kir2.1 in neonatal P4 mice had a similar effect on the biophysical properties of the IHC basolateral membrane (Fig EV1D) as that described in P9-P11 IHCs (Fig 1D). We also found that the number of presynaptic ribbons, postsynaptic glutamate receptors and their co-localization in prehearing IHCs was not affected by the overexpression of Kir2.1 channels (Fig EV2A–D). In agreement with the normal morphological profile of the synapses, exocytosis in IHCs was not significantly different between the two genotypes ($P = 0.4709$, 2-way ANOVA, Fig EV2E and F). These data indicate that the overexpression of Kir2.1 channels is not affecting the expression of the ion channels that are normally present in developing IHCs or their ribbon synapses.

We then investigated the ability of IHCs to fire intrinsic and induced Ca^{2+} action potentials at near body temperature (34–37°C) with an *in vivo* endolymph-like solution surrounding the IHC hair bundles. IHC Ca^{2+} action potentials are elicited by the opening of Ca^{2+} channels that activate at around −60 mV (Marcotti *et al*, 2003a). During the first postnatal week, the ionic composition of the endolymph is comparable to that of the perilymph, which contains 1.3 mM Ca^{2+} (Wangemann & Schacht, 1996). Under these recording conditions, spontaneous Ca^{2+} spiking activity was recorded from P4 control IHCs (Fig 2A). The mean spike frequency of IHCs was 2.19 ± 1.09 Hz ($n = 6$), and the coefficient of variation (CV) was 1.30 ± 0.63 ($n = 7$, duration of the recordings 45–101 s), which being greater than one, is indicative of a bursting pattern of activity as previously demonstrated (Johnson *et al*, 2011). In P4 *Kir2.1*-OE mice, due to the more hyperpolarized resting V_m, IHCs do not fire action potentials spontaneously, although they retain the ability to do so during large depolarizing current injections (Fig 2B). During the second postnatal week, spontaneous action potentials in IHCs disappear when using *ex vivo* cochlear preparations, which is due to a progressive hyperpolarization of the IHC resting V_m (Marcotti *et al*, 2003b) but could still be elicited by depolarizing current injections (Fig 2C, top panel). This membrane hyperpolarization is likely to be compensated *in vivo* by the resting open probability of the mechanoelectrical transducer (MET) channel (Johnson *et al*, 2012). This is because *in vivo* the endolymphatic Ca^{2+} concentration during the second postnatal week has been estimated to be near 0.3 mM (Johnson *et al*, 2012), which will increase the open probability of the MET channels and thus cause the IHCs to depolarize to around the action potential threshold (Fig 2C, bottom panel; for spike frequency and CV see Fig 7E). We found that the IHCs from *Kir2.1*-OE mice failed to elicit spontaneous action potentials even in the estimated 0.3 mM endolymphatic Ca^{2+} concentration, causing the IHCs to remain silent at rest (Fig 2D).

IHCs are surrounded by nonsensory cells in the greater epithelial ridge (GER, also known as Kölliker's organ: Fig 2E). The release of ATP from nonsensory cells of the GER leads to spatially and temporally coordinated Ca^{2+} waves that propagate across the epithelium and cause IHCs to depolarize as much as 28 mV (Tritsch *et al*, 2010). This depolarization has been shown to produce periodic bursts of Ca^{2+} action potentials in IHCs (Tritsch *et al*, 2007, 2010; Wang *et al*, 2015; Johnson *et al*, 2017). The frequency and duration of the Ca^{2+} waves in the nonsensory cells were not affected by the overexpression of the Kir2.1 channels (Fig EV3A and B). Therefore, we investigated whether the more hyperpolarized IHCs (by about 10 mV) from *Kir2.1*-OE mice retained the ability to respond to spontaneous Ca^{2+} waves originating in the GER. We found that in the presence of the estimated *in vivo* endolymph-like Ca^{2+} (0.3 mM), the Ca^{2+} signals caused by the opening of the Ca^{2+} channels in IHCs followed very closely the time course of the Ca^{2+} wave originating in the GER in both control (Fig 2F) and *Kir2.1*-OE (Fig 2G) P7-P9 mice. Moreover, the correlation between IHC Ca^{2+} activity and Ca^{2+} waves in the nonsensory cells was unaffected in Kir2.1 mice (Fig EV3C). This indicates that the large depolarization caused by the extracellular input of the nonsensory cells was necessary and sufficient to depolarize the IHCs in *Kir2.1*-OE mice and cause the opening of voltage-gated calcium channels.

Progressive loss of mechanoelectrical transduction in IHCs lacking intrinsic Ca^{2+} action potentials

MET currents were recorded from apical-coil IHCs by displacing their hair bundles using a 50 Hz sinusoidal force stimulus from a

 © 2023 The Authors

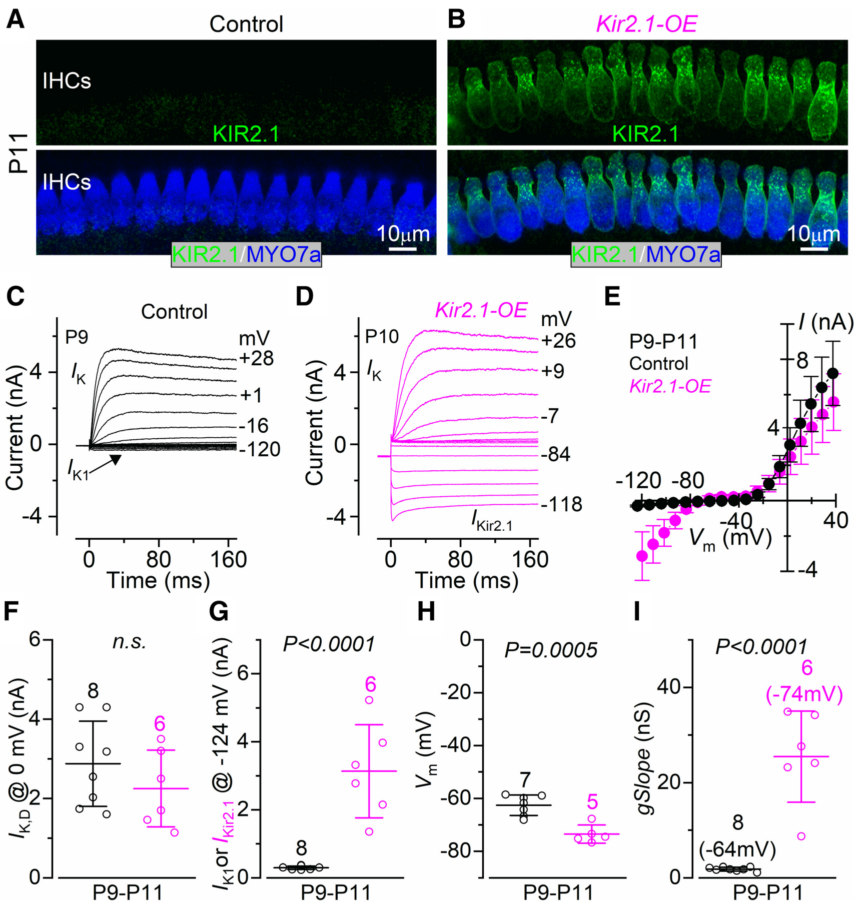

Figure 1. Basolateral membrane properties of IHCs overexpressing Kir2.1 channels.

A, B Maximum intensity projections of confocal z-stacks taken from the apical cochlear region of control (A) and littermate *Kir2.1* overexpressing (B, *Kir2.1*-OE) mice at postnatal day 11. Inner hair cells (IHCs) were stained with antibodies against Kir2.1 (green) and the hair cell marker Myo7a (blue). At least 3 mice for each genotype were used. Scale bars: 10 µm.

C, D Currents from IHCs of control (C, P9) and *Kir2.1*-OE (D, P10) prehearing mice. Currents were elicited by using depolarizing and hyperpolarizing voltage steps, with a nominal increment of 10 mV, from a holding potential of −84 mV. Test potentials are shown next to some of the traces. Note that the large inward rectifier Kir2.1 current is only present in the IHC of the *Kir2.1*-OE mouse (D). The outward current is primarily carried by a delayed rectifier current I_K. I_{K1} identifies the small inwardly rectifying K^+ current normally expressed in IHCs.

E Steady-state current–voltage curves obtained from IHCs of control (P9-P11) and *Kir2.1*-OE (P9-P11) mice.

F, G Size of the total steady-state outward (F, I_K: Control 2.88 ± 1.07 nA, $n = 8$; *Kir2.1*-OE 2.25 ± 0.97 nA, $n = 6$) and inward (G, Control, I_{K1}: 0.30 ± 0.05 nA, $n = 8$; *Kir2.1*-OE, $I_{Kir2.1}$: 3.13 ± 1.37 nA, $n = 6$) K^+ currents measured at 0 mV and − 124 mV, respectively. n.s: $P = 0.2836$.

H Resting membrane potential (V_m) measured in IHCs from Control (−62.6 ± 3.8 mV, $n = 7$) and *Kir2.1*-OE (−73.5 ± 3.5 mV, $n = 5$).

I Slope conductance of the current measured at around the respective resting V_m (Control 1.8 ± 0.4 nS, $n = 8$; *Kir2.1*-OE 25.5 ± 9.6 nA, $n = 6$).

Data information: In panels F–I, data are shown as means ± SD, and the single cell value recordings (open symbols) are plotted with the average data. All statistical tests were performed using the Student's *t*-test. The number of IHCs investigated is shown above the average data points (6 control and 3 *Kir2.1*-OE mice).
Source data are available online for this figure.

piezo-driven fluid jet (Corns *et al*, 2018; Carlton *et al*, 2021). A large MET current was elicited in all IHCs tested from control (Fig 3A) and *Kir2.1*-OE (Fig 3B) mice at P6-P7 when their stereociliary bundles were moved towards the taller stereocilia (i.e., in the excitatory direction) at negative membrane potentials. By stepping the membrane potential from −124 mV to more depolarized values in 20 mV increments, the transducer current decreased in size at first and then reversed near 0 mV in IHCs from both genotypes (Fig 3A–C), consistent with the nonselective permeability of MET channels to cations. The maximal MET current at both −124 mV and +96 mV was not significantly different between control and littermate *Kir2.1*-OE mice ($P = 0.2269$ and $P = 0.3620$, respectively, *t*-test, Fig 3D). The resting open probability of the MET channel, which is derived from the current flowing through open transducer channels in the absence of mechanical stimulation (arrows: Fig 3A and B), was also not significantly different between the IHCs from the two genotypes (−124 mV: $P = 0.3766$; +96 mV: $P = 0.2846$, Fig 3E). At P8-P9, the size of the MET currents became more variable in the IHCs overexpressing the Kir2.1 channel, with some cells showing a third of the current recorded from control mice (Fig 4A–C). Overall, the size of

© 2023 The Authors

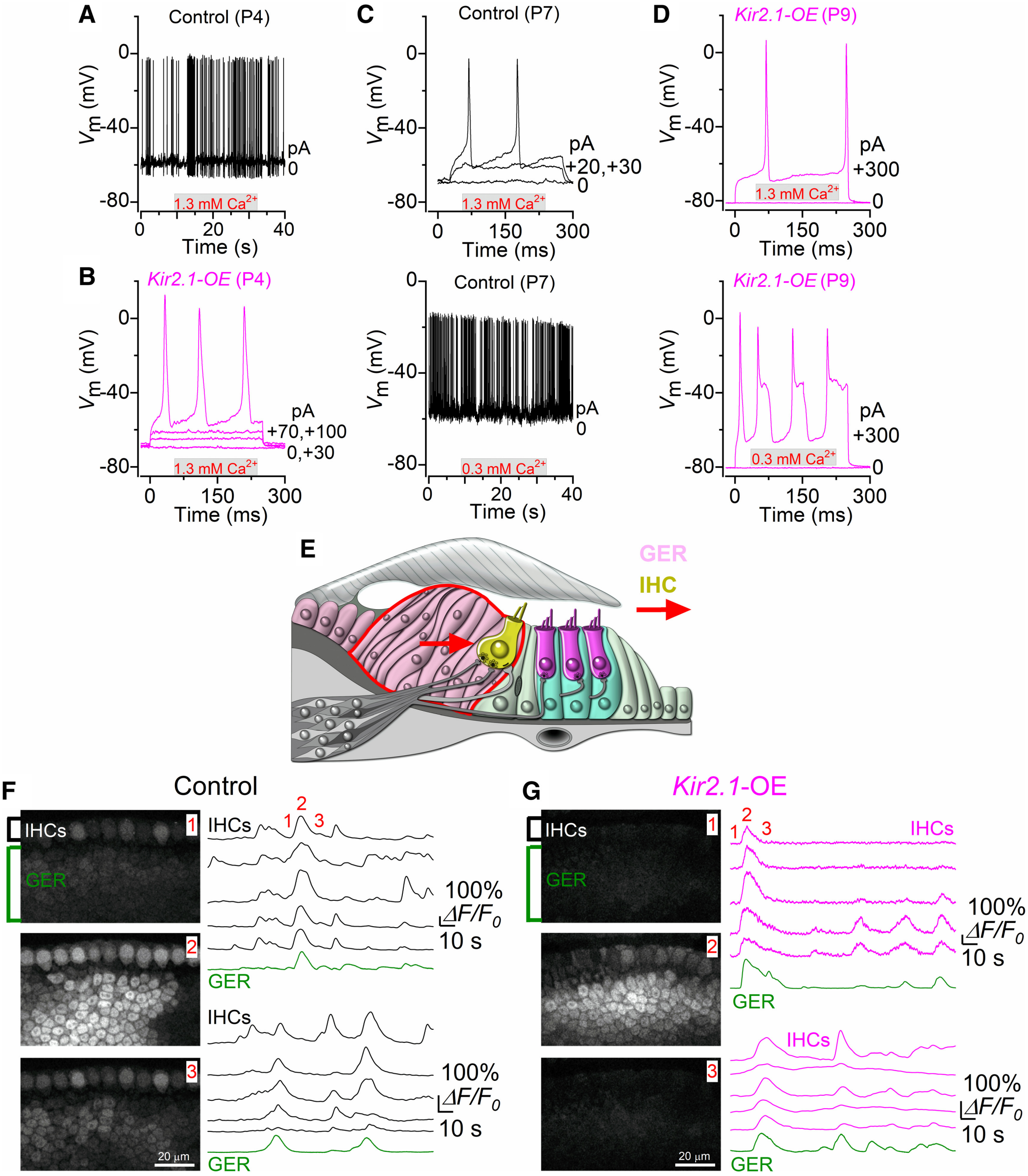

Figure 2. Kir2.1 overexpression prevents spontaneous, but not induced, Ca^{2+} action potentials in IHCs.

A, B Whole-cell recordings of Ca^{2+} action potential activity in apical-coil IHCs from P4 control (A) and *Kir2.1*-OE (B) mice in the presence of 1.3 mM Ca^{2+} in the extracellular solution and at body temperature. Note that IHCs from control mice (A) fire spontaneous action potentials (40 s out of 142 s recording time), while those from overexpressing *Kir2.1* IHCs (B) require a substantial current injection to elicit any spikes. For voltage-clamp data see also Fig EV1C–I. Data in Panel A,B, and Fig EV1C–I were obtained from 8 control IHCs (7 mice) and 11 *Kir2.1*-OE IHCs (6 mice).

C, D Calcium action potentials in IHCs from control (C) and *Kir2.1*-OE (D) mice during the second postnatal week. IHC voltage responses were recorded during the application of a solution containing 1.3 mM Ca^{2+} (top panels) or 0.3 mM Ca^{2+} (bottom panels). The latter Ca^{2+} concentration (0.3 mM), which was used to mimic the estimated *in vivo* Ca^{2+} concentration in the endolymphatic compartment (Johnson *et al*, 2012), caused control IHCs, but not those from *Kir2.1*-OE mice, to elicit spontaneous action potentials (40 s out of 56 s recording time).

E Diagram showing a cross-section of an immature organ of Corti. IHCs: inner hair cells; GER: greater epithelial ridge, which includes nonsensory cells surrounding the IHCs. Red arrows indicate the propagation of ATP-induced Ca^{2+} waves from the GER towards the IHCs, which leads to their depolarization (Tritsch *et al*, 2007; Wang *et al*, 2015; Johnson *et al*, 2017).

F, G Representative $\Delta F/F_0$ traces from the IHCs and GER of P7-P9 control (F) and *Kir2.1*-OE (G) mice in the presence of 0.3 mM Ca^{2+}. Spontaneous ATP-dependent Ca^{2+} waves from the GER (green traces) were eliciting coordinated Ca^{2+} signals in the IHCs from both controls and *Kir2.1*-OE mice. For each genotype, two separate sets of recordings from 2 mice are shown (top and bottom right), with the top traces being linked to the images on the left: before [1], during [2] and after [3]) the generation of a large Ca^{2+} wave from the GER. For details about the frequency and duration of the Ca^{2+} waves, and the number of mice and recordings see Fig EV3. All recordings were obtained at body temperature. Traces are computed as pixel averages of regions of interest centred on IHCs.

Source data are available online for this figure.

© 2023 The Authors

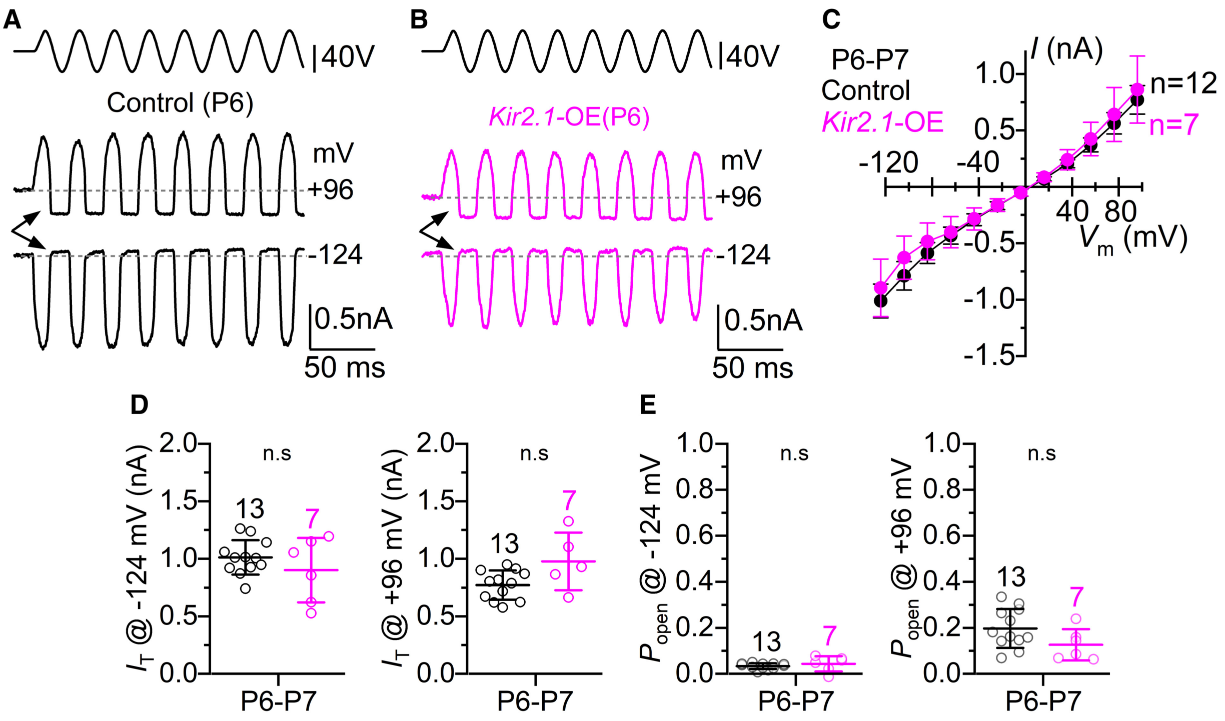

Figure 3. Mechanoelectrical transduction in Kir2.1 overexpressing IHCs is normal during the first postnatal week.

A, B Saturating MET currents recorded from apical IHCs of P6 control (A) and *Kir2.1*-OE (B) mice in response to 50 Hz sinusoidal force stimuli to the hair bundles at membrane potentials of −124 and +96 mV. Driver voltage (DV) stimuli to the fluid jet are shown above the traces, with positive deflections of the DV being excitatory. The arrows indicate the closure of the transducer channel in response to inhibitory bundle stimuli at −124 and +96 mV.

C Peak-to-peak MET current–voltage curves from P6-P7 apical-coil IHCs of 7 control (12 IHCs) and 3 littermate *Kir2.1*-OE mice (7 IHCs). Recordings were obtained by mechanically stimulating the hair bundles of IHCs at the same time as stepping their membrane potential from −124 mV to +96 mV in 20 mV increments. The two sets of data are not significantly different: $P = 0.6320$, 2-way ANOVA.

D Maximum size of the MET current recorded at −124 mV (left panel) and +96 mV (right panel) in IHCs from both genotypes.

E Resting open probability (*Popen*) of the MET current in IHCs from the two genotypes measured at −124 mV (left) and +96 mV (right). The resting open probability was calculated by dividing the resting MET current (the difference between the current level before the stimulus, indicated by the dashed line, and the current level at the negative phase of the stimulus when all channels are closed) by the maximum peak-to-peak MET current.

Data information: All comparisons in panels D and E are not significantly different between the two genotypes (D: −124 mV: $P = 0.2269$; +96 mV: $P = 0.3620$; E: −124 mV: $P = 0.3766$; +96 mV: $P = 0.2846$, *t*-test). In panels C-E, data are shown as means ± SD, and the single cell value recordings (open symbols) are plotted behind the average data. The number of IHCs investigated is shown above the averaged data points from 7 control and 3 *Kir2.1*-OE mice.
Source data are available online for this figure.

the MET current was significantly reduced (−124 mV: $P < 0.0001$; +96 mV: $P = 0.0003$, Fig 4D) and the resting open probability increased (−124 mV: $P = 0.0080$; +96 mV: $P = 0.0130$, Fig 4E) in IHCs from *Kir2.1*-OE mice compared with controls. Since an increased resting open probability of the MET channel could be associated with changes, specifically a reduction, in the free Ca^{2+} inside the stereocilia, we tested this possibility by changing the intracellular Ca^{2+} buffering capacity by using different concentrations of the fast Ca^{2+} chelator BAPTA. Increasing the intracellular BAPTA from 0.1 to 5 mM significantly augmented the resting open probability of the MET channel in IHCs from both genotypes, although at both BAPTA concentrations it was significantly higher in the IHCs of *Kir2.1*-OE mice (Fig EV4). This indicates that in the absence of spontaneous intrinsic firing activity in the IHCs of *Kir2.1*-OE mice, the MET channels are likely to have a reduced Ca^{2+} sensitivity during the second postnatal week. By P10-P11, we found that the MET current in the IHCs of *Kir2.1*-OE mice was very small or absent (Fig 4F–I). At this stage, IHCs from P10-P11 *Kir2.1*-OE mice also failed to load with the styryl dye FM1-43 (Fig 4J), which is a permeant blocker of the hair cell MET channel and functions as an optical readout for the presence of the resting MET current (Gale *et al*, 2001).

IHCs from *Kir2.1*-OE mice undergo progressive loss and fusion of the stereocilia

We investigated whether the rapid reduction in the MET current was caused by defects in the growth and/or maintenance of the stereociliary bundles in IHCs. Using scanning electron microscopy we found that the hair bundles of the IHCs from *Kir2.1*-OE mice were able to develop a staircase structure composed of rows of stereocilia that were indistinguishable from those present in control cells (arrows: Fig 5A and B). This is consistent with the presence of a normal MET current at least up to the end of the first postnatal

© 2023 The Authors

week (Fig 3). However, from about P9 onwards, IHCs from *Kir2.1*-OE mice started to lose the shorter third row of stereocilia (Fig 5B). A few IHCs also started to exhibit stereocilia fusion, which became more pronounced at older ages. By P26, none of the IHCs in the *Kir2.1*-OE mice showed normal-looking bundles, which instead exhibited profound stereocilia fusion (Fig 5C and D). *Kir2.1*$^{+/-}$ mice that were not crossed with *Otof*$^{tTA+/-}$ mice showed normal hair-bundle development when treated with DOX, highlighting the specificity of the *Kir2.1*-OE strategy (Fig EV5). These data indicate that spontaneous Ca^{2+} actions potential activity during the second

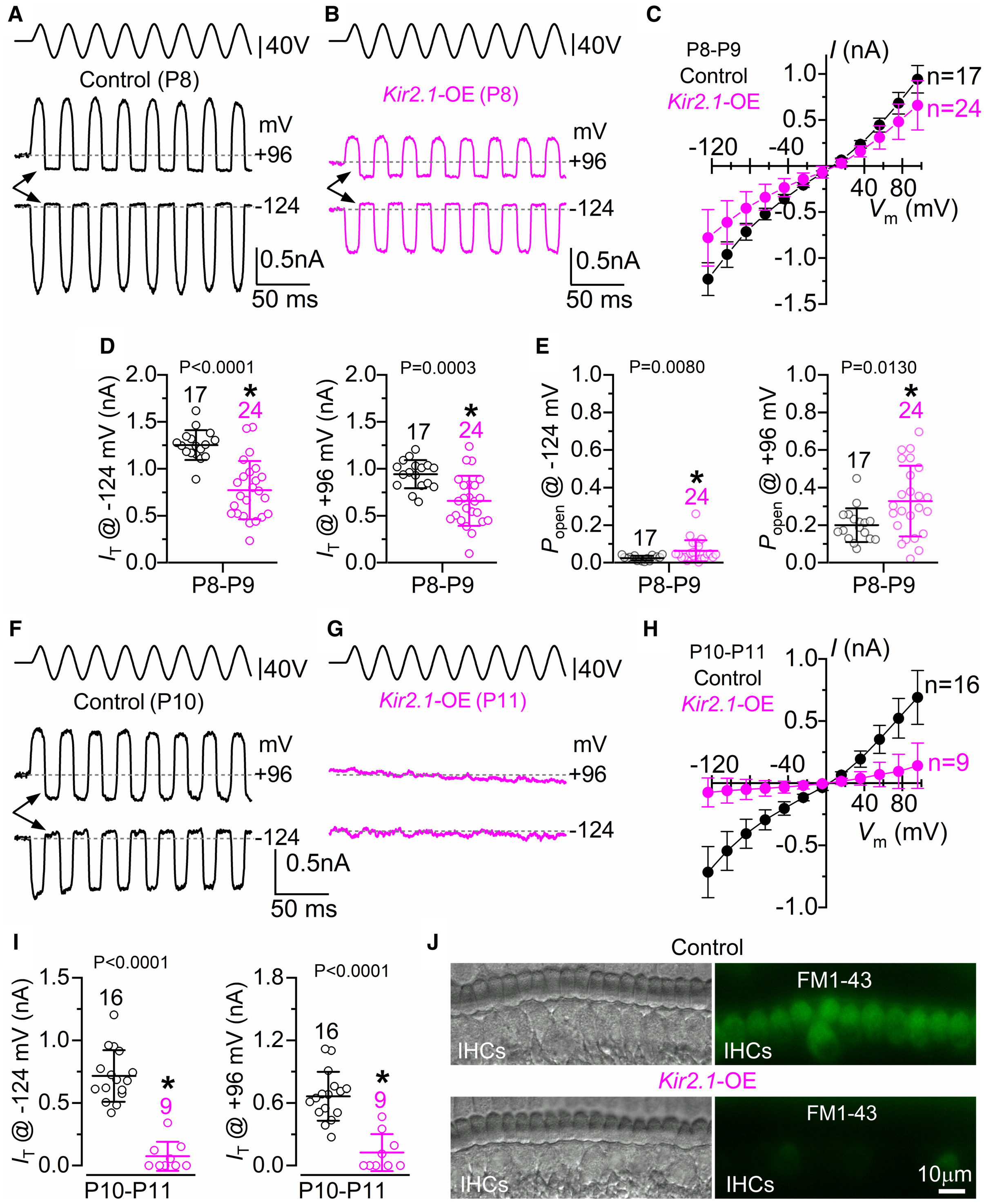

Figure 4.

 © 2023 The Authors

Figure 4. Rapid disappearance of the MET current in *Kir2.1* overexpressing IHCs during the second postnatal week.

A, B Saturating MET currents recorded from apical IHCs of P8 control (A) and *Kir2.1*-OE (B) mice. IHC hair bundles were stimulated as described in Fig 3.
C Peak-to-peak MET current–voltage curves from P8-P9 apical-coil IHCs of 12 control (17 IHCs) and 13 littermates *Kir2.1*-OE mice (24 IHCs). The two sets of data are significantly different: $P < 0.0001$, 2-way ANOVA.
D The maximum size of the MET current measured in IHCs at −124 mV (left panel) and +96 mV (right panel) from *Kir2.1*-OE mice was significantly reduced compared to that of control cells.
E The resting open probability (P_{open}) of the MET current in IHCs was significantly increased in *Kir2.1*-OE compared with control cells at both −124 mV (left) and +96 mV (right).
F, G Saturating MET currents recorded from apical IHCs of control (F, P10) and *Kir2.1*-OE (G, P11) mice. IHC hair bundles were stimulated as described in Fig 3.
H Peak-to-peak MET current–voltage curves from P10-P11 apical-coil IHCs of 13 control (16 IHCs) and 4 littermate *Kir2.1*-OE mice (9 IHCs). The two sets of data are significantly different: $P < 0.0001$, 2-way ANOVA.
I The maximum size of the MET current measured in IHCs at −124 mV (left panel) and +96 mV (right panel) from *Kir2.1*-OE mice is significantly reduced compared to that of control cells.
J Example of FM1-43 uptake by IHCs from P11 control (top) and *Kir2.1*-OE (bottom) mice, showing the lack of fluorescence labeling in the latter, which is an indication of the lack of MET channels open at rest at this stage in the IHCs overexpressing the Kir2.1 channels. At least 3 mice for each genotype were used.

Data information: In panels D, E, I, data are shown as means ± SD, and the single cell value recordings (open symbols) are plotted with the average data. The number of IHCs investigated is shown above the average data points from 12 control and 13 littermates *Kir2.1*-OE mice (panels D and E) and 13 control and 4 littermate *Kir2.1*-OE mice (panel I). Statistical tests in panels D, E, I was done using the *t*-test. The * defines the presence of statistical significance, with the *P*-value shown above the data.
Source data are available online for this figure.

postnatal week is required for the maintenance of the stereociliary bundles in the mature IHCs.

Localization of bundle proteins is not affected in *Kir2.1*-OE mice

To establish whether the progressive loss and fusion of the stereocilia were linked to the mislocalization of some of the key proteins expressed in the hair bundles, we performed immunostaining experiments on both genotypes. Stereocilia fusion has previously been documented in hair cells from mice lacking *Myo6*, the gene encoding for the (F-actin) minus end-directed unconventional myosin 6 (Self *et al*, 1999). We found that MYOSIN VI was expressed in the stereocilia of the IHCs from both control and littermate *Kir2.1*-OE mice (Fig 6A and C). The disorganized hair bundle of the IHCs from *Kir2.1*-OE mice also showed a normal distribution at the tip of the taller rows of stereocilia of EPS8, MYOSIN XV-isoform 1 and WHIRLIN (Fig 6B and D); key proteins required for growth and maintenance of stereocilia (Belyantseva *et al*, 2005; Delprat *et al*, 2005; Manor *et al*, 2011; Zampini *et al*, 2011).

IHC action potentials exert their developmental role in stereocilia maintenance during a critical period

Next, we tested whether Ca^{2+} action potential activity in IHCs was regulating hair-bundle maintenance during a specific time window or "critical period" of prehearing development. This was achieved by downregulating *Kir2.1*-OE *in vivo* by removing DOX from the drinking water at a specific developmental time point. Considering that the hair bundles of the IHCs in *Kir2.1*-OE mice were able to acquire a staircase structure and have normal MET current at the end of the first postnatal week, we sought to test whether the role of the Ca^{2+}-action potentials was restricted to the second week, just before hearing onset at ~P12.

As for the above investigation, DOX was continuously supplied to the females from the time of conception, but for this set of experiments, it was then removed from the drinking water when the pups were P5. We found that 2–3 days without DOX was sufficient to strongly downregulate Kir2.1 from the membrane of the IHCs of *Kir2.1*-OE mice (Fig 7A and B). This indicates that the overexpression of Kir2.1 in IHCs was primarily occurring during the first postnatal week under these conditions. We then investigated whether the downregulation of Kir2.1 channels following DOX removal (Appendix Fig S2) re-established the ability of IHCs to fire intrinsic spontaneous action potentials. In 1.3 mM extracellular Ca^{2+}, action potentials only occurred during depolarizing current injections in the IHCs from both control and *Kir2.1*-OE mice in the second postnatal week (Fig 7C and D; see also Fig 2C and D). In the presence of the *in vivo* endolymph-like Ca^{2+} concentration (0.3 mM), spontaneous intrinsic firing was present not only in the IHCs of control mice (Fig 7E; see also Fig 2C) but also in *Kir2.1*-OE mice (Fig 7F). For long-lasting current clamp recordings, spikes occurred in a bursting pattern in both controls and *Kir2.1*-OE mice. The mean spike frequency (1.17 ± 0.47 Hz, $n = 4$) and CV (1.12 ± 0.17, $n = 4$ IHCs, duration of the recordings 62–125 s) in control mice were not significantly different from those measured in Kir2.1-OE mice (frequency: 1.29 ± 0.83 Hz, $n = 5$ IHCs, $P = 0.7766$; CV: 1.15 ± 0.11, $n = 5$, duration of the recordings 32–101 s, $P = 0.7954$). The IHC resting membrane potential was not significantly different between control and *Kir2.1*-OE mice in the presence of both 1.3 mM and 0.3 mM Ca^{2+} (Fig 7G). These data indicate that the removal of DOX was effective in downregulating Kir2.1 channels and restoring the normal physiology of the IHCs. We also found that when DOX was removed at P5, IHCs were able to maintain their hair-bundle structure after the onset of hearing (Fig 7H–K). These findings indicate that Ca^{2+} regulation via action potentials in IHCs is required for the final maturation and maintenance of the hair bundles after a critical point just before hearing onset.

Identification of genes regulated by the intrinsic Ca^{2+} action potentials using RNA-sequencing

To understand the molecular pathways underpinning the changes in the hair-bundle structure observed in the absence of the intrinsic Ca^{2+} action potential activity in IHCs, we performed RNA-seq on P9 controls and littermate *Kir2.1*-OE mice. At this age, most of the hair bundles still showed a normal-looking structure, but with some IHCs having lost the 3rd row of stereocilia and some showing stereocilia fusion (Fig 5B). This was associated with the onset of MET

© 2023 The Authors

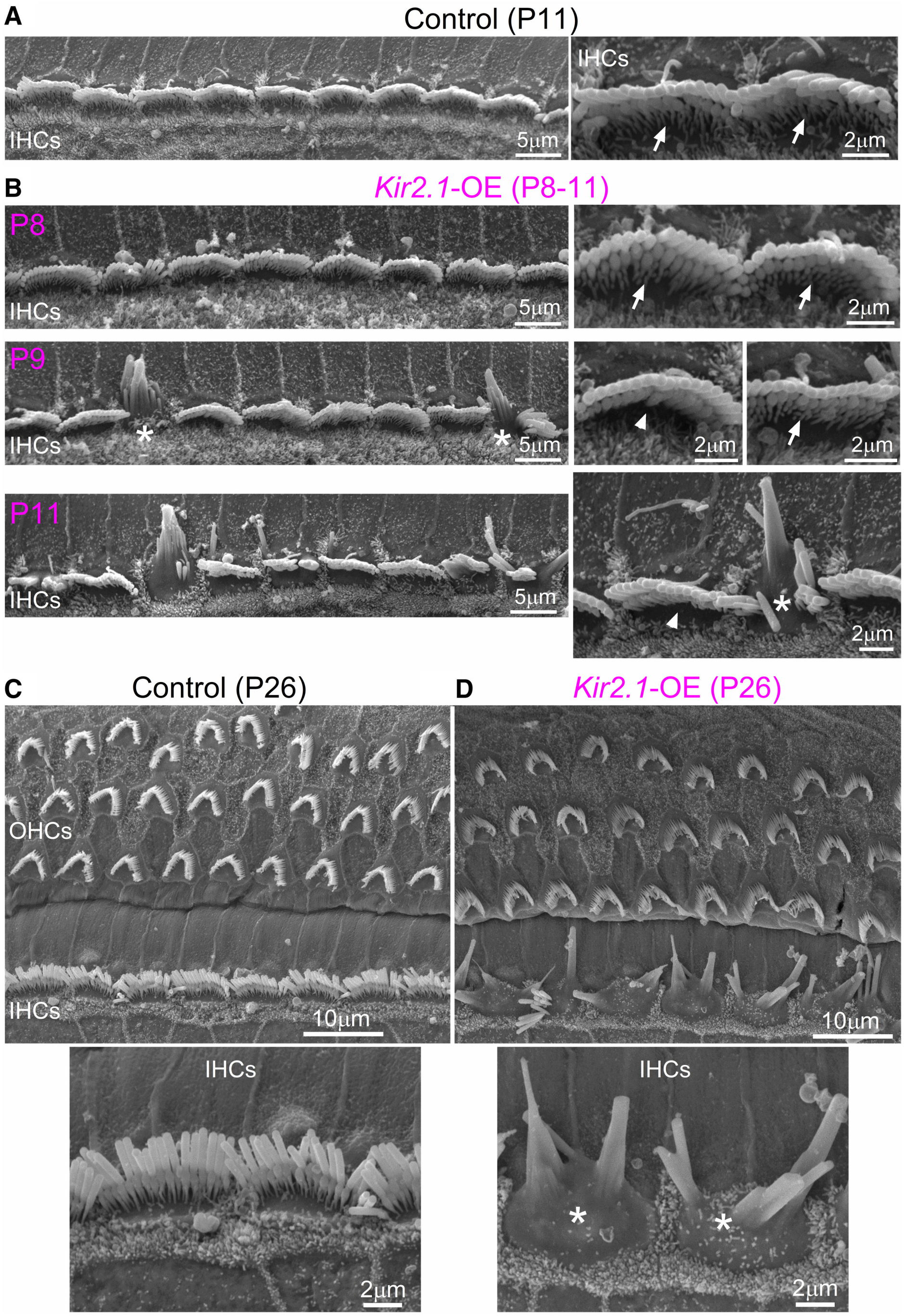

Figure 5. IHC bundle morphology progressively deteriorates in *Kir2.1* overexpressing mice.

A, B Scanning electron microscope (SEM) images showing the IHC hair-bundle structure in the apical coil of the cochlea of P11 control (A) and P8-P11 *Kir2.1*-OE (B) mice. Control IHCs and the large majority of P8 IHCs from *Kir2.1*-OE mice show a normal hair-bundle structure composed of three rows of stereocilia: tall, intermediate and short (arrows). From about P9 in *Kir2.1*-OE mice, some IHCs start to lose the third row of stereocilia (arrowheads) and some already exhibit some fusion of the stereocilia (asterisk). These changes in hair-bundle structure became more prominent at P11. At least 3 mice for each genotype were used. In these panels and those below, asterisks are used to define some of the abnormal hair bundles.

C, D SEM images of both IHCs and OHCs from the cochlea of P26 control (C, upper panel) and P26 *Kir2.1*-OE (D, upper panel) mice. Lower panels show a higher-magnification view of the hair bundle of IHCs from both genotypes, highlighting the profound disruption of the stereocilia in IHCs overexpressing Kir2.1 channels. At least 3 mice for each genotype were used.

Source data are available online for this figure.

 © 2023 The Authors

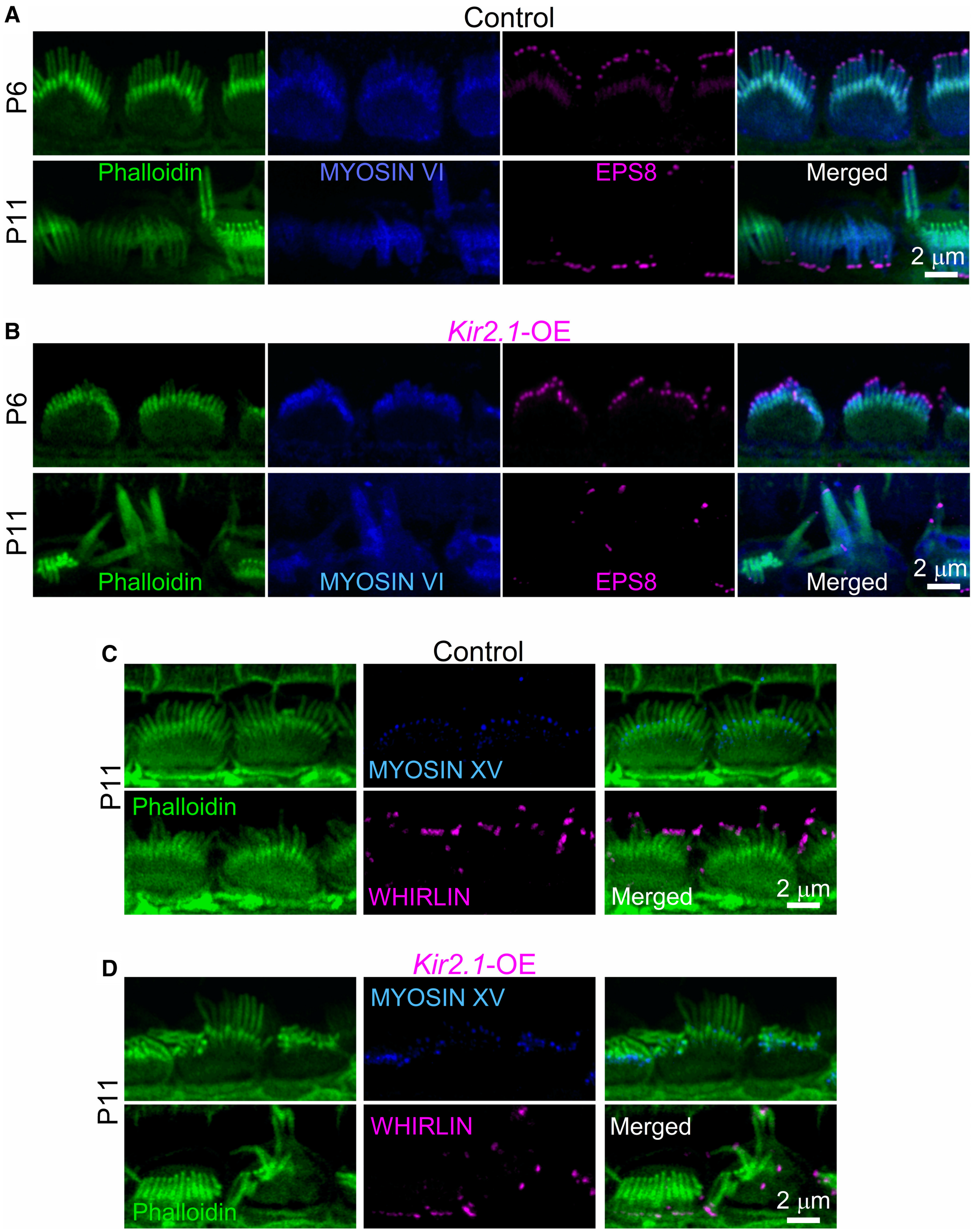

Figure 6. Hair-bundle proteins involved in stereociliary elongation are not affected in IHCs from *Kir2.1*-OE mice.

A, B Maximum intensity projections of confocal z-stacks showing images of the hair bundles from apical-coil IHCs of P6 and P11 control (A) and *Kir2.1*-OE (B) mice immunostained with antibodies against MYOSIN VI (blue) and EPS8 (magenta). At least 3 mice for each genotype were used.

C, D Confocal images of the hair bundles of P11 IHCs from control (C) and *Kir2.1*-OE (D) mice immunostained with antibodies against MYOSIN XV-isoform 1 (blue) and WHIRLIN (magenta). In all panels (A–D), stereocilia are labeled with phalloidin (green). Note that despite the disrupted hair-bundle structure in the IHCs overexpressing Kir2.1 channels; the stereocilia retained a normal distribution of these bundle proteins. At least 3 mice for each genotype were used.

Source data are available online for this figure.

current reduction in at least some of the IHCs (Fig 4A–E). We reasoned that by profiling animals at this age we could understand the early molecular response that leads to abnormal hair-bundle morphology.

RNA-sequencing was performed on three replicates, each with eight pooled organs of Corti from four mice. Total RNA was extracted and sent for library preparation and sequencing. Sequence data were mapped to the mouse genome (mm10) using the

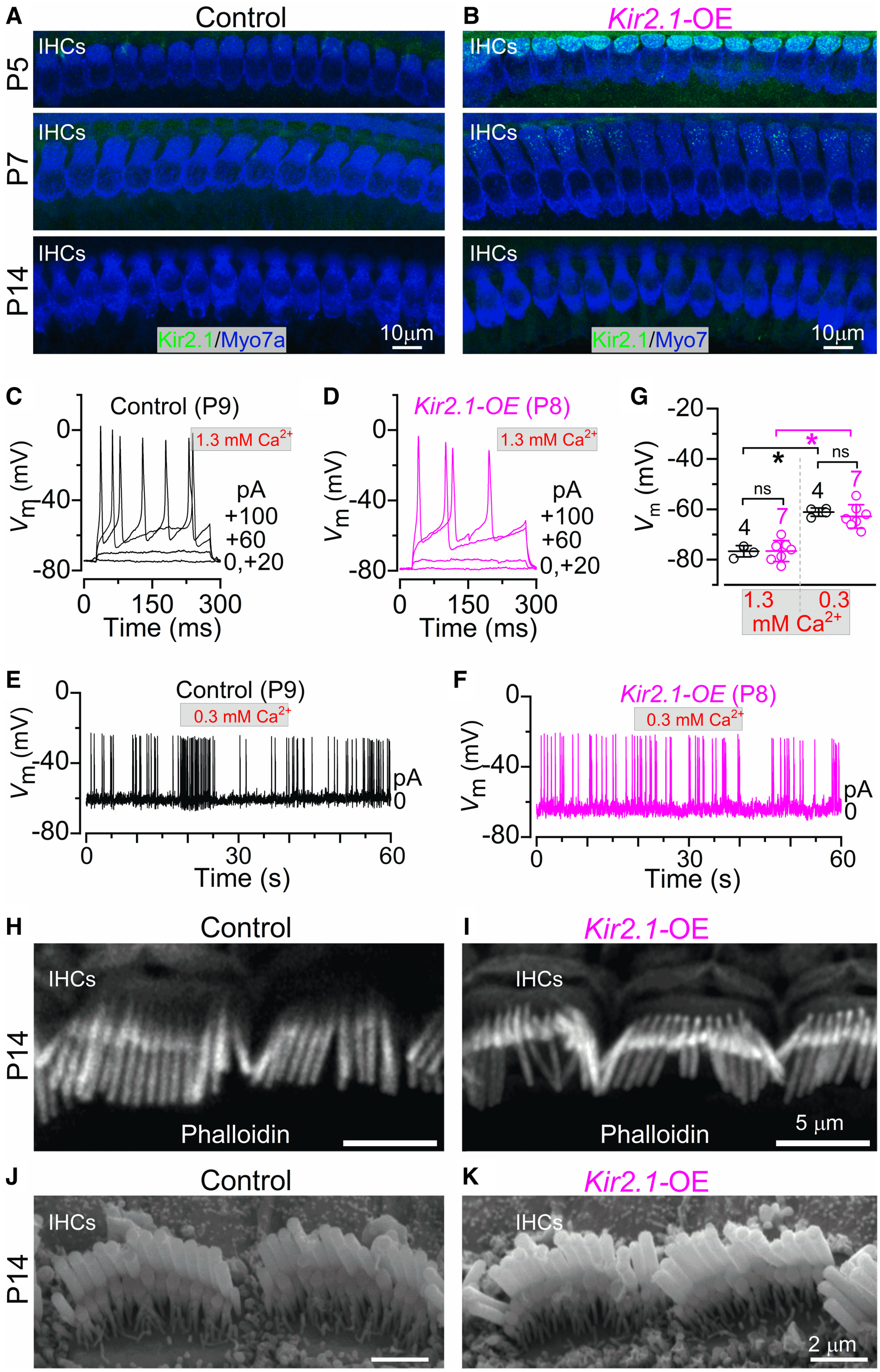

Figure 7.

© 2023 The Authors

Figure 7. Ca^{2+} spikes in IHCs regulate bundle morphology over a critical period during the second postnatal week of development.

A, B Maximum intensity projections of confocal z-stacks showing the IHCs of the apical cochlear region from control (A) and *Kir2.1*-OE (B) pups with the females being in the continuous presence of DOX in the drinking water from conception up to when the pups were P5 (upper panels). Middle and bottom panels show IHCs at P7 and P14 following the removal of DOX at P5 for both control (A) and *Kir2.1*-OE mice (B). IHCs were stained with antibodies against the K^+ channel Kir2.1 (green) and Myosin 7a (Myo7a, blue: cell marker). Note that after 2 days following the removal of DOX, *Kir2.1* overexpression was already largely downregulated. At least 3 mice for each genotype were used.

C, D Calcium action potentials in IHCs from control (C) and *Kir2.1*-OE (D) mice during the second postnatal week (P8–P9). IHC voltage responses were recorded during the application of 1.3 mM Ca^{2+} extracellular solution. The voltage-clamp data recorded from the same two IHCs displayed in panels C and D are shown in Appendix Fig S2; the IHCs from *Kir2.1*-OE mice show a strongly reduced Kir2.1 current. DOX was removed from the drinking water at P5.

E, F Spontaneous Ca^{2+} action potentials in IHCs from control (E, 60 s out of 92 s recording time) and *Kir2.1*-OE (F, 60 s out of 103 s recording time) mice during the second postnatal week in the presence of the *in vivo* endolymph-like 0.3 mM Ca^{2+}. Note that in contrast to when DOX was present throughout development (Fig 2A–D), the removal of DOX at P5 restored the ability of IHCs from *Kir2.1*-OE mice to generate spontaneous intrinsic Ca^{2+} action potentials.

G IHC resting membrane potentials from 2 control (4 IHCs) and 3 *Kir2.1*-OE (7 IHCs) mice in the presence of 1.3 mM Ca^{2+} (left) or 0.3 mM Ca^{2+} (right) in the extracellular solution. One-way ANOVA followed by the Bonferroni's post-test: ns, $P > 0.9990$ (1.3 mM Ca^{2+}); ns, $P = 0.8864$ (0.3 mM Ca^{2+}); all other comparisons were $*P < 0.0001$.

H, I Maximum intensity projections of confocal z-stacks showing images of the hair bundles from apical-coil IHCs of P14 control (H) and *Kir2.1*-OE (I) mice stained with phalloidin. DOX was removed from the mother's drinking water when the pups were P5. At least 3 mice for each genotype were used.

J, K SEM images showing the normal structure of the hair bundles of the IHCs in the apical coil of the cochlea of P14 control (J) and P14 *Kir2.1*-OE (K) mice. DOX was removed from the mother's drinking water when the pups were P5. Note that the morphological profile of the hair bundles in IHCs is comparable between control and *Kir2.1*-OE mice, indicating that the removal of the intrinsic Ca^{2+} action potentials prior to the second postnatal week has no effect on the mechanoelectrical transduction apparatus. At least 3 mice for each genotype were used.

Source data are available online for this figure.

NextFlow RNA pipeline and gene counts were performed using Salmon (see Materials and Methods). These raw counts were then used as the input for differential gene expression analysis using DeSEQ2 (Love *et al*, 2014). After performing principal component analysis (PCA) on the top 1,000 expressed genes in the samples, we observed a clear separation between the different genotypes with PC1, which separated *Kir2.1*-OE and control mice, explaining 85% of the observed variance. Conversely, PC2, which mostly separated the different biological replicates, explained 8% of variance between samples (Fig 8A). As expected, we observed a 13-fold increase in *Kcnj2* (Fig 8B), validating the overexpression of the *Kir2.1*.

We next performed differential gene expression analysis ($P_{adj} <$ 0.05 and fold-changes >1.5), yielding 589 upregulated genes and 30 downregulated genes (Dataset EV1; Appendix Fig S3). Pathway analysis showed an enrichment of GO terms related to cell morphogenesis, actin filament-based processes and Rho-GTPase signaling (Fig 8C). Among the differentially expressed genes were 118 upregulated genes with annotations related to actin filament or microtubule regulation (Fig 8D). We also noted several genes related to the Golgi body and the trans-Golgi network (TGN), for example, *Golga3*, *Golga4*, and *Trip11*, which are all hypothesized to play a role in maintaining Golgi structure. In line with the stereocilia phenotype, we observed the upregulation of some components of the stereocilia, *Myo7a* and *Pcdh15* (2.25 and 2.15-fold, respectively) (Fig 8E).

We also performed network analysis on known protein–protein interactions on the differentially expressed genes (Fig 8G, Dataset EV2). Chromatin remodeling genes were also overrepresented among the upregulated genes, including DNA methylation (*Dnmt1*, *Dnmt3a*) and demethylation (*Tet1*, *Tet2*, *Tet3*) and histone modifying enzymes (*Hdac4*, *Setd2*, *Setd5*). Several components of the LINC complex that connects the nuclear lamina to the cytoskeletal network, including the subunits of the laminin complex (*Lama 1*, *2*, *4*, *5*, *Lamb1*,*2*, *Lamc1 and 2*) and the Nesperin family (*Syne1*, *Syne2*, *Syne3*) that connect the cytoskeletal network to laminin, were upregulated (Fig 8G, Dataset EV2). Mechanical signals are directly transduced from extracellular stimulus to the nuclear interior through the interaction of the nesperin proteins (Khilan *et al*, 2021). Moreover, the maintenance of nuclear structure and

Figure 8. RNA-sequencing reveals upregulation of microtubule and cytoskeletal genes in *Kir2.1*-OE mice.

A PCA plot of each RNA library. Each point represents one pool of 4 mice (8 cochleae) for both control and littermates *Kir2.1*-OE mice. Principal components were calculated using the top 1,000 genes after rlog transformation, using DESeq2. The percent variance for each principal component is noted on the axis.

B Transcripts per million (TPM) counts of *Kcnj2* for each RNA-seq library. Counts were performed using Salmon, and length normalized. Each point represents a single library. Bars represent the mean ± SD.

C Top GO terms associated with the significantly ($P_{adj.} < 0.05$) upregulated genes in the *Kir2.1*-OE mouse.

D Heatmap of the counts for each RNA library for the 118 genes associated with microtubule or cytoskeletal processes. Each row is z-scored. 118 genes were all differentially expressed as per the previous analysis.

E TPM counts of *Pcdh15*, *Myo7a*, *Macf1*, *Ank2*, *Myh9*, *Map1a*, *Map1b*, *Cep290* and *Sptbn1* for each RNA-seq library. Counts were performed using Salmon and normalized to the length of the gene sequence. Each point represents a single library. Bars represent the mean ± SD. The numbers above the data represent the multiple hypotheses corrected adjusted *P*-values derived from DESeq2 using the Wald test.

F Results of the top 15 motifs that were overrepresented in the TSS of the upregulated genes in *Kir2.1*. Enrichment analysis was performed using HOMER, in the ± 2,000 bp region around the transcriptional start site of upregulated genes.

G A network rendering of the top GO processes associated with the upregulated gene set. Networks were seeded with the upregulated genes and their nearest interaction and visualized using Cytoscape.

Source data are available online for this figure.

© 2023 The Authors

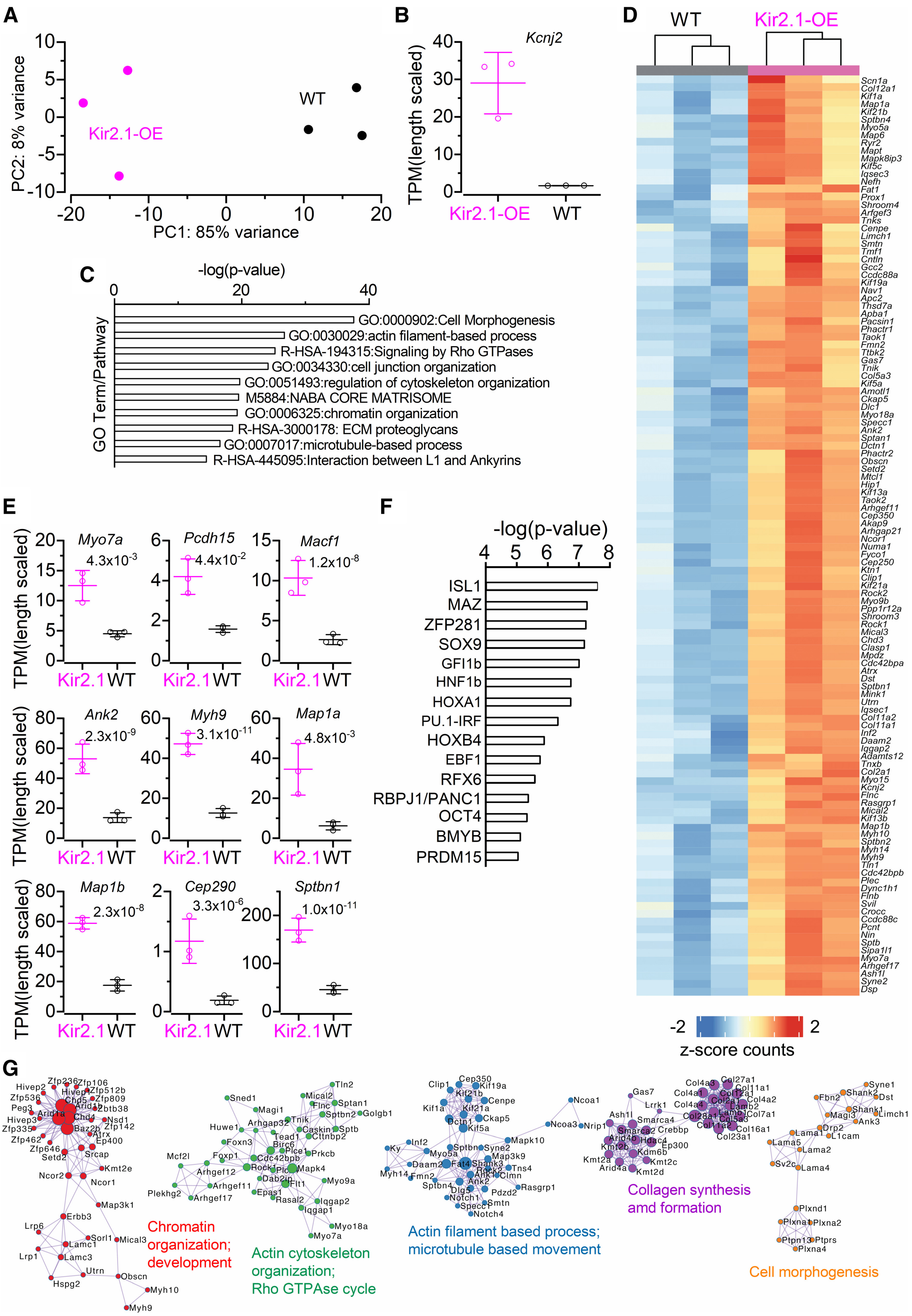

Figure 8.

 © 2023 The Authors

organization is regulated by laminins, which also help to transduce mechanical strain forces into a transcriptional response.

We next sought to determine which transcription factors (TFs) might be mediating the upregulated genes in *Kir2.1* overexpression. Using the list of 589 upregulated genes, we used HOMER (Heinz *et al*, 2010) to scan the region ± 2,000 bp from the transcriptional start site (TSS) of each gene for TF binding motifs. Within the top fifteen enriched motifs were several hair cell-enriched TFs, such as *Isl1*, *Sox9*, and *Gfi1* (Fig 8F). Several classic targets of SOX9, including *Acan*, *Col2a1*, *Col4a1*, *Col5a1*, *Col11a1/2*, *Col23a1*, and several ankyrin family proteins (genes: *Ank1*, *Ank2*, *Ankrd11*, *Ankrd12*) and *Myo9b*, were found to be SOX9 target genes in chromatin immunoprecipitation with sequencing (ChIP-seq) studies conducted in rib chondrocytes (Ohba *et al*, 2015). Of the 602 upregulated genes, 65% overlapped with SOX9 target genes in chondrocytes. Similarly, SOX9 ChIP-seq in the developing testis found SOX9 bound on *Myo7a* (Li *et al*, 2014). We also observed enrichment for the RFX family, which plays a conserved role in ciliogenesis in many different organisms (Lemeille *et al*, 2020).

Discussion

Here we show that spontaneous intrinsic Ca^{2+} action potential activity present in the developing IHCs, and thus Ca^{2+} regulation, is crucial for the final stages of maturation and maintenance of the stereociliary hair bundles. The absence of the intrinsic action potentials during the second postnatal week led to a progressive reabsorption of stereocilia in the short 3rd row and a fusion of the tallest rows, generating "giant" stereocilia. The functional consequence of this hair-bundle disruption was a complete loss of mechanoelectrical transduction prior to the onset of hearing at P12. Furthermore, we show that this intrinsic regulation of IHC development occurs during a critical time window that spans the second postnatal week of development, just before hearing onset. The RNA-sequencing analysis highlighted that absence of intrinsic APs caused the upregulation of genes involved in cytoskeleton and Rho-GTPase-related pathways, several of which have not been previously associated with cochlear development.

Calcium-dependent activity in the developing cochlea

The initial morphological and functional differentiation of cochlear sensory hair cells depends on intrinsic genetic programs that are coordinated by a combination of transcription factors, including *Atoh1* (Bermingham *et al*, 1999), *Helios* (Chessum *et al*, 2018) and *Tbx2* (García-Añoveros *et al*, 2022), and microRNAs such as *miR-96* (Kuhn *et al*, 2011). However, evidence from other sensory systems, especially from the visual system (e.g., Grubb & Thompson, 2004; Blankenship & Feller, 2010), shows that the final maturation of sensory pathways is driven by experience-independent Ca^{2+}-dependent activity, which occurs during a critical period of development. This early electrical activity has been shown to regulate several cellular responses (Berridge *et al*, 2000), including the remodeling of synaptic connections (Zhang & Poo, 2001) and ion-channel expression (Moody & Bosma, 2005).

In the mammalian cochlea, spontaneous Ca^{2+}-dependent action potentials have been recorded throughout the postnatal development of the IHCs (Kros *et al*, 1998; Glowatzki & Fuchs, 2000; Beutner & Moser, 2001; Marcotti *et al*, 2003a; Brandt *et al*, 2007). The firing activity of neighboring IHCs is normally synchronized by spontaneous intercellular Ca^{2+} signaling originating in the nonsensory cells via the release of ATP (Tritsch *et al*, 2007; Johnson *et al*, 2011, 2017; Wang *et al*, 2015; Eckrich *et al*, 2018). ATP acts on purinergic autoreceptors expressed in the nonsensory cells surrounding the IHCs, which leads to the opening of TMEM16A Ca^{2+}-activated Cl^{-} channels and the efflux of K^{+} in the intercellular space, causing IHC depolarization (Wang *et al*, 2015). Although Ca^{2+} action potential activity in developing IHCs has been linked to the refinement of the tonotopic organization in the brainstem (Clause *et al*, 2014, 2017; Müller *et al*, 2019; Maul *et al*, 2022) and auditory neuron survival (Zhang-Hooks *et al*, 2016), its direct role in regulating and/or maintaining IHC development is still largely unknown. A previous study has shown that increasing the IHC firing activity prevented linearization of their exocytotic Ca^{2+} dependence in the adult cochlea (Johnson *et al*, 2013), although both the intrinsic and ATP-dependent mechanisms could have contributed.

The mouse model used in this study (*Kir2.1*-OE) has allowed us to specifically silence the intrinsic Ca^{2+} action potentials in developing IHCs *in vivo* while retaining the ability of nonsensory cells to depolarize the IHCs via ATP-dependent Ca^{2+} signaling. We found that the absence of spontaneous Ca^{2+} action potentials that are intrinsically generated by the IHCs prevented the full maturation and maintenance of the hair bundles in IHCs, thus abolishing the mechanoelectrical transducer current that is required for the conversion of acoustic stimuli into electrical signals. We found that such crucial control over the hair-bundle structure and function is only established after a critical time point in the second postnatal week, just before hearing onset.

Role of Ca^{2+}-dependent action potentials in the maturation of hair cells

Calcium-dependent electrical activity regulates several cellular responses (Berridge *et al*, 2000). Changes in intracellular Ca^{2+} signals mediated by L-type Ca^{2+} channels have been implicated in regulating gene expression in many intracellular pathways including those associated with remodeling and development (Bading *et al*, 1993; Dolmetsch *et al*, 1997; Fields *et al*, 2005; Hagenston & Bading, 2011). Here we show using RNA-seq analysis that the dysregulation of Ca^{2+} in prehearing IHCs, which only retain the extrinsic modulation of the Ca^{2+} signals from the nonsensory cells, led to 589 upregulated and 30 downregulated genes.

One of the characteristic phenotypes of the *Kir2.1*-OE mouse cochlea was the formation of giant or fused stereocilia, which was previously reported in knockout mice for the protein TRIOBP (Kitajiri *et al*, 2010), which is a component of the stereocilia rootles (Pacentine *et al*, 2020), for the unconventional MYO6 (Self *et al*, 1999) that localizes at the base and all along the length of the stereocilia (Hertzano *et al*, 2008) and for a protein associated with the shaft connectors located between stereocilia (PTPRQ, Goodyear *et al*, 2003). RNA-seq analysis did not show any significant changes in the genes encoding the above three proteins in the cochlea of *Kir2.1*-OE mice but did identify 118 upregulated genes with annotations related to actin filament or microtubule regulation. This included cytoskeletal genes *Mapt*, *Sptb*, *Plec*, and *Nefh*, Kinesin

© 2023 The Authors

superfamily proteins, which are microtubule-dependent molecular motors (*Kif1a*, *Kif5a*, *Kif5c*, *Kif21a*, *Kif21b*), and several components of the Rho-GTPase pathway (*Rock1*, *Iqgap1*, *Iqgap2*, *Itpr1*, *Itpr2*, *Itpr3*, *Arhgap13*, *Argef11*, *Argef17*, *Trio*, *and Kalrn*). Although most of the identified genes are possible novel candidates involved in hair cell development, we found some that have previously been associated with hair-bundle morphology. For example, the actin-binding protein spectrin isoform SPTBN1 (*Sptbn1*), which is expressed in the rootlets actin filaments of the stereocilia (Furness *et al*, 2008), and together with TRIOBP contributes to strengthen their insertion point into the apical membrane of the hair cells (Pacentine *et al*, 2020), is required for the correct hair-bundle morphology. In the absence of SPTBN1 mice are deaf (Liu *et al*, 2019). Furthermore, the actin crosslinking family protein 7 (ACF7) and the microtubule-associated protein 1A (MAP-1A), which are encoded by the genes *Macf1* and *Macp1a*, respectively, are also involved in the organization of the cuticular plate of the hair cells (Jaeger *et al*, 1994; Antonellis *et al*, 2014), which is the point of stereocilia insertion of the hair bundles. Finally, the non-muscle myosin Type IIA (MYH9) has been linked to both syndromic and nonsyndromic hearing loss due to the disruption of the hair cell stereociliary bundles (Mhatre *et al*, 2006). Among the identified transcription factors, we found enrichment for regulatory factor X (RFX), which plays a role in ciliogenesis in many different organisms (Lemeille *et al*, 2020). In mammals, *Rfx3* is involved in ciliary assembly and motility, and *Rfx4* is known to modulate Shh signaling and regional control of ciliogenesis (Ashique *et al*, 2009). Moreover, recent work has shown that the RFX family is essential for hearing in mice, with mice at 3 months of age showing a loss of stereocilia structure (Elkon *et al*, 2015).

Altogether, these results indicate that the intrinsic Ca^{2+}-dependent action potential activity in IHCs during the second postnatal week is necessary to drive their full morphological and functional maturation into auditory sensory receptors. The absence of such activity led to the upregulation of the genetic pathways involved in the maintenance of cytoskeletal homeostasis, possibly as an attempt to repair or compensate for the progressive deterioration of the actin-based hair bundles. Moreover, we found that the MET channel of the IHCs from *Kir2.1*-OE mice acquire a reduced Ca^{2+} sensitivity, which could be a potential compensatory mechanism for maintaining resting MET current size as the MET current is rapidly declining. Although genetic compensation responses following the mutation of genes have been described in many organisms including zebrafish and mice (e.g., El-Brolosy & Stainier, 2017; Buglo *et al*, 2020), their underlying mechanisms remain largely unknown.

Materials and Methods

Reagents and Tools table

Reagent/Resource	Reference or Source	Identifier or Catalog Number
**Experimental Models**		
Otof^{tTA} (*M. musculus*)	Ozgene Pty Ltd	n/a
TetO-Kir2.1-IRES-tauLacZ	The Jackson Lab	009136
**Recombinant DNA**		
Example: pCMV-BE3	Addgene	Cat #73021
**Antibodies**		
Mouse-IgG1 anti-Eps8	BD Biosciences	610143
Rabbit-IgG anti-Whirlin	gift from Dr. Thomas Friedman	n/a
Rabbit-IgG anti-Myo6	Proteus Biosciences	25-6791
Rabbit-IgG anti-Myo15 isoform 1	gift from Dr. Thomas Friedman	n/a
Mouse-IgG1 anti-Kir2.1	Alomone Lab	APC026
Mouse-IgG1 anti-CtBP2	BD Biosciences	612044
Mouse-IgG2a anti-GluR2	Millipore	MAB397
**Chemicals, enzymes, and other reagents**		
Doxycycline	Karidox 100 mg/ml	
Vitamins	Thermo Fisher	11120-037
Amino acids	Thermo Fisher	11130-036
DMEM/F12	Sigma	D8062
Fluo-4 AM	Thermo Fisher	F14201
FM1-43	Molecular Probes	T3163
Texas Red-X phalloidin	ThermoFisher	T7471

© 2023 The Authors

Reagents and Tools table (continued)

Reagent/Resource	Reference or Source	Identifier or Catalog Number
**Software**		
pClamp 10	Molecular Devices	RRID:SCR_011323
Origin	OriginLab	RRID:SCR_014212
Python 2.7	Python Software Foundation	RRID:SCR_008394
ImageJ	NIH	RRID:SCR_003070
DeSeq2	Love *et al*, 2014	
Metascape	Zhou *et al*, 2019	
Reactome	Gillespie *et al*, 2022	
HOMER	Heinz *et al*, 2010	
**Other**		
Digidata 1440A	Molecular Devices	RRID:SCR_021038
Two-photon laser-scanning microscope (Bergamo II system B232)	Thorlabs Inc	
Vega3 LMU scanning electron microscope	Tescan	
LSM 880 AiryScan microscope	Zeiss	
RNeasy Plus Micro Kit	Qiagen	
NovaSeq sequencer	Illumina	

Methods and Protocols

Ethics statement

Animal work was licensed by the Home Office under the Animals (Scientific Procedures) Act 1986 (PPL_PCC8E5E93) and was approved by the University of Sheffield Ethical Review Committee (180626_Mar). For *ex vivo* experiments mice were killed by cervical dislocation followed by decapitation. Mice had free access to food and water and a 12 h light/dark cycle.

Transgenic mice

The transgenic mouse line $Otof^{rtTA}$ expressing rtTA driven by the *Otof* promoter was constructed by Ozgene Pty Ltd (Bentley WA, Australia). In these mice, the expression of a target gene is controlled by a reverse tetracycline-controlled transactivator *rtTA* (Tet-On system: Baron & Bujard, 2000). *Otof* encodes for the ribbon synaptic Ca^{2+} sensor otoferlin, which in the cochlea is expressed exclusively in hair cells but primarily in IHCs from around birth (Roux *et al*, 2006). Homozygous $Otof^{rtTA}$ mice were paired with heterozygous tet_o*-Kir2.1-IRES-tau-lacZ* mice (*Kir2.1*: Jackson laboratories, 009136, Yu *et al*, 2004). Both mouse lines ($Otof^{rtTA}$ and *Kir2.1*) were maintained on the C57BL/6N background. The resultant compound heterozygous mice, which we named *Kir2.1*-OE (*Kir2.1*-OverExpression) mice for simplicity, allowed cell-specific overexpression of the inward rectifier K^+ channel Kir2.1 in the IHCs when mice were treated with doxycycline (DOX). Littermate heterozygous $Otof^{rtTA}$ mice treated with DOX were used as controls. Pregnant, breast-feeding females and weaned pups (controls and *Kir2.1*-OE) were given 0.5 mg/ml of DOX daily in their drinking water, a dose that was previously optimized for the mouse cochlea (Johnson *et al*, 2013).

Tissue preparation

Cochleae were dissected out from the inner ear of the mouse using an extracellular solution composed of (in mM): 135 NaCl, 5.8 KCl, 1.3 $CaCl_2$, 0.9 $MgCl_2$, 0.7 NaH_2PO_4, 5.6 D-glucose, 10 HEPES. Sodium pyruvate (2 mM), amino acids, and vitamins were added from concentrates (Thermo Fisher Scientific, UK). The pH was adjusted to 7.48 with 1 M NaOH (osmolality ~308 mOsm/kg). The dissected cochleae were transferred to a microscope chamber and immobilized via a nylon mesh attached to a stainless-steel ring as previously described (Marcotti *et al*, 2003b). The chamber (volume ~ 2 ml) was perfused from a peristaltic pump and mounted on the stage of an upright microscope (Olympus BX51, Japan; Leica DMLFS, Germany) equipped with Nomarski Differential Interference Contrast (DIC) optics (60× or 64× water immersion objective) and 15× eyepieces. The microscope chamber was continuously perfused with the extracellular solution by a peristaltic pump (Cole-Palmer, UK).

Whole-cell electrophysiology

Patch clamp experiments were performed from hair cells positioned at the 9–12 kHz region of the cochlear apical coil (Müller *et al*, 2005). Recordings were performed at room temperature (20–24°C) using an Optopatch amplifier (Cairn Research Ltd, UK) as previously described (Jeng *et al*, 2020; Carlton *et al*, 2021). Patch pipettes were pulled from soda glass capillaries, which had a typical resistance in the extracellular solution of 2–3 MΩ. The intracellular solution used for the patch pipette contained (in mM): 131 KCl, 3 $MgCl_2$, 1 EGTA-KOH, 5 Na_2ATP, 5 HEPES, 10 Na-phosphocreatine (pH was adjusted with 1 M KOH to 7.28; 294 mOsm/kg). Data acquisition was controlled by pClamp software using Digidata 1440A (Molecular Devices, USA). In order to reduce the electrode capacitance, patch electrodes were coated with surf wax (Mr Zoggs SexWax, USA). Recordings were low-pass filtered at 2.5 kHz (8-pole Bessel), sampled at 5 kHz, and stored on a computer for offline analysis (Clampfit, Molecular Devices; Origin 2021: OriginLab, USA). Membrane potentials under voltage-clamp conditions were corrected offline for the residual series resistance R_s after compensation (usually 80%)

© 2023 The Authors

and the liquid junction potential (LJP) of −4 mV, which was measured between electrode and bath solutions. Voltage-clamp protocols are referred to a holding potential of −84 mV unless otherwise stated.

Real-time changes in membrane capacitance (ΔC_m) were tracked at body temperature as previously described (Johnson *et al*, 2005, 2017). Briefly, a 4 kHz sine wave of 13 mV RMS was applied to IHCs from the holding potential of −81 mV and was interrupted for the duration of the voltage step. The capacitance signal from the Optopatch was filtered at 250 Hz and sampled at 5 kHz. ΔC_m was measured by averaging the C_m trace over a 200 ms period following the voltage step and subtracting the pre-pulse baseline. Data were acquired using pClamp software and a Digidata 1440A (Molecular Devices). ΔC_m experiments were performed during the local perfusion of the IHCs with 30 mM TEA, 15 mM 4-AP (Fluka) to block the outward K^+ currents (Johnson *et al*, 2005), and 5 mM CsCl to block the inward rectifier K^+ current (Marcotti *et al*, 1999).

For mechanoelectrical transducer (MET) current recordings, the hair bundles of hair cells were displaced using a fluid-jet system from a pipette driven by a 25 mm diameter piezoelectric disc (Corns *et al*, 2014, 2018; Carlton *et al*, 2021). For these experiments, the intracellular solution contained (in mM): 131 CsCl, 3 $MgCl_2$, 1 EGTA-KOH, 5 Na_2ATP, 5 HEPES, 10 Na-phosphocreatine (pH was adjusted with 1 M CsOH to 7.28; 290 mOsm/kg). The extracellular solution was as described above, although for most of the recordings we included 5 mM CsCl, which was used to block the inward rectifier K^+ current (Marcotti *et al*, 1999). In order to maintain the osmolality of the extracellular solution constant, NaCl was reduced to 130 mM in this case.

The fluid-jet pipette tip had a diameter of 8–10 μm and was positioned near the hair bundles to elicit a maximal MET current (typically 10 μm). Mechanical stimuli were applied as 50 Hz sinusoids (filtered at 1 kHz, 8-pole Bessel). Prior to the positioning of the fluid jet by the hair bundles, any steady-state pressure was removed. The use of the fluid jet allows for the efficient displacement of the hair bundles in both the excitatory and inhibitory directions, which is essential to perform reliable measurements of the resting open probability of the MET channels.

Two-photon confocal Ca^{2+} imaging

Acutely dissected cochleae were incubated for 40 min at 37°C in DMEM/F12, supplemented with fluo-4 AM at a final concentration of 10 μM (Thermo Fisher Scientific) as recently described (Ceriani *et al*, 2019). The incubation medium contained also pluronic F-127 (0.1%, w/v) and sulfinpyrazone (250 μM) to prevent dye sequestration and secretion. Calcium signals were recorded using a two-photon laser-scanning microscope (Bergamo II System B232, Thorlabs Inc., USA) based on a mode-locked laser system operating at 925 nm, 80-MHz pulse repetition rate, < 100-fs pulse width (Mai Tai HP DeepSee, Spectra-Physics, USA). Images were captured with a 60× objective (LUMFLN60XW, Olympus, Japan) using a GaAsp PMT (Hamamatsu) coupled with a 525/40 bandpass filter (FF02-525/40-25, Semrock). Images were analyzed offline using custom-built software routines written in Python (Python 2.7, Python Software Foundation) and ImageJ (NIH). Calcium signals were measured as relative changes in fluorescence emission intensity ($\Delta F/F_0$).

Scanning electron microscopy (SEM)

The isolated inner ear was very gently perfused with fixative for 1–2 min through the round window. A small hole in the apical portion of the cochlear bone was made prior to perfusion to allow the fixative to flow out from the cochlea. The fixative contained 2.5% vol/vol glutaraldehyde in 0.1 M sodium cacodylate buffer plus 2 mM $CaCl_2$ (pH 7.4). The inner ears were then immersed in the above fixative and placed on a rotating shaker for 2 h at room temperature. After fixation, the organ of Corti was exposed by removing the bone from the apical coil of the cochlea and then immersed in 1% osmium tetroxide in 0.1 M cacodylate buffer for 1 h. For osmium impregnation, which avoids gold coating, cochleae were incubated in solutions of saturated aqueous thiocarbohydrazide (20 min) alternating with 1% osmium tetroxide in buffer (2 h) twice (the OTOTO technique: Furness & Hackney, 1986). The cochleae were then dehydrated through an ethanol series and critical point dried using CO_2 as the transitional fluid (Leica EM CPD300) and mounted on specimen stubs using conductive silver paint (Agar Scientific, Stansted, UK). The apical coil of the organ of Corti was examined at 10 kV using a Tescan Vega3 LMU scanning electron microscope in the electron microscopy unit at the University of Sheffield.

Immunofluorescence microscopy

As for SEM, the isolated inner ear was initially gently perfused with 4% paraformaldehyde in phosphate-buffered saline (PBS, pH 7.4) through the round window. Following this initial short fixation, the inner ear was fixed for 20 min at room temperature and then washed three times in PBS for 10 min. The apical coil of the organ of Corti was then washed in PBS, removed by fine dissection, and incubated in PBS supplemented with 5% normal goat or horse serum and 0.5% Triton X-100 for 1 h at room temperature. The samples were immunolabeled with primary antibodies overnight at 37°C, washed three times with PBS, and incubated with the secondary antibodies for 1 h at 37°C. Antibodies were prepared in 1% serum and 0.5% Triton X-100 in PBS. Primary antibodies were mouse-IgG1 anti-Eps8 (1:1,000, BD Biosciences, 610,143), rabbit-IgG anti-WHIRLIN (1:200, gift from Dr. Thomas Friedman, NIH, USA); rabbit-IgG anti-MYO6 (1:150, Proteus Biosciences, 25–6,791); rabbit-IgG anti-MYO15-isoform 1 (1:1,000, gift from Dr. Thomas Friedman, NIH, USA) mouse-IgG1 anti-Kir2.1 channel (1:100, Alomone Lab, Israel, APC026); mouse IgG1 anti-CtBP2 (1:200, Biosciences, #612044) and mouse IgG2a anti-GluR2 (1:200, Millipore, MAB397). F-actin was stained with Texas Red-X phalloidin (1:400, ThermoFisher, T7471) in the secondary antibody solution. Secondary antibodies were species-appropriate Alexa Fluor or Northern Lights secondary antibodies. Samples were mounted in VECTASHIELD (H-1000). The images from the apical cochlear region (8–12 kHz) were captured with Nikon A1 confocal microscope equipped with a Nikon CFI Plan Apo 60× Oil objective or a Zeiss LSM 880 AiryScan equipped with Plan-Apochromat 63× Oil DIC M27 objective for super-resolution images of hair bundles. Both microscopes are part of the Wolfson Light Microscope Facility at the University of Sheffield. Image stacks were processed with Fiji ImageJ software.

FM1-43 staining

A 3 mM stock solution of the dye FM1-43 (T3163, Molecular Probes) was prepared in water. The dissected organs of Corti (aged P11–P12) were transferred to the bottom of a chamber filled with

 © 2023 The Authors

extracellular solution, and held in position using a nylon mesh, as described above (see above: *Tissue preparation*). All experiments were performed at room temperature (20–24°C), as previously described (Gale *et al*, 2001). Briefly, the solution bathing the cochleae was very rapidly exchanged with that containing 3 μM FM1-43 for 10 s and immediately washed several times with normal extracellular solution. The cochleae were then viewed with an upright microscope equipped with epifluorescence optics and FITC filters (excitation 488 nm, emission 520 nm) using a 63× water immersion objective and a CCD camera.

RNA isolation and library preparation

The sensory epithelium from four control and four littermates *Kir2.1-OE* mice under DOX were microdissected in DNase-free ice-cold PBS 1× and immediately snap frozen in liquid nitrogen. RNA was extracted using RNeasy Plus Micro Kit (Qiagen) according to the manufacturer's instructions. RNA quantity was established using a Nanodrop spectrophotometer and RNA integrity number (RIN) was calculated using a BioAnalyzer. All samples had RIN score greater than 9.1. Preparation of the mRNA library was performed using poly A enrichment and sequenced on the Illumina NovaSeq sequencer using paired-end 150 bp reads.

RNA-sequencing analysis and differential gene expression

The sequencing libraries were processed using the nf-core RNA pipeline (Ewels *et al*, 2020, https://nf-co.re/rnaseq/usage) using the standard parameters. Reads were mapped to the mouse genome (mm10). The resulting gene counts were determined using Salmon (Patro *et al*, 2017) and used for downstream analysis with DESeq2 (Love *et al*, 2014). Metascape (Zhou *et al*, 2019) and Reactome (Gillespie *et al*, 2022) were used to query for enriched GO terms and pathways in the list of differentially expressed genes. HOMER (Heinz *et al*, 2010) was used to find known and *de novo* motifs among the upregulated genes in a 2000 bp window up and downstream of the transcriptional start site (TSS).

Statistical analysis

Statistical comparisons of means were made by the Student's two-tailed *t*-test or, for multiple comparisons, the analysis of variance (one-way or two-way ANOVA followed by a suitable post-test) and Mann–Whitney *U* test (when normal distribution could not be assumed) were used. $P < 0.05$ was selected as the criterion for statistical significance. Only mean values with a similar variance between groups were compared. Average values are quoted in text and figures as means ± S.D. Animals of either sex were randomly assigned to the different experimental groups. No statistical methods were used to define sample size, which was determined based on previously published similar work from our laboratory. Animals were taken from several cages and breeding pairs over a period of several months. Most of the electrophysiological and morphological (but not imaging) experiments were performed blind to animal genotyping and in most cases, experiments were replicated at least 3 times.

Data availability

The data that support the findings of this study are available from the corresponding author. RNA-sequencing data have been deposited in GEO under accession number (GSE215951; http://www.ncbi.nlm.nih.gov/geo/query/acc.cgi?acc=GSE215951).

Expanded View for this article is available online.

Acknowledgements

The authors thank Michelle Bird (University of Sheffield) for her assistance with the mouse husbandry, and Catherine Gennery and Laila Moushtaq-Kheradmandi for their genotyping work. This work was supported by the BBSRC (BB/S006257/1 and BB/T004991/1) and Wellcome Trust (224326/Z/21/Z) to WM, the MRC (MR/S002510/1) to MM. AU was supported by a PhD studentship from the MRC DiMeN Doctoral Training Partnership to WM. For the purpose of Open Access, the author has applied a CC BY public copyright license to any author accepted manuscript version arising from this submission.

Author contributions

Adam J Carlton: Conceptualization; data curation; formal analysis; validation; investigation; methodology; writing – original draft; writing – review and editing. **Jing-Yi Jeng:** Data curation; formal analysis; validation; investigation; methodology; writing – review and editing. **Fiorella C Grandi:** Data curation; formal analysis; validation; investigation; methodology; writing – review and editing. **Francesca De Faveri:** Data curation; formal analysis; validation; investigation; methodology; writing – review and editing. **Federico Ceriani:** Data curation; formal analysis; validation; investigation; methodology; writing – review and editing. **Lara De Tomasi:** Investigation; methodology. **Anna Underhill:** Data curation; formal analysis; investigation; methodology. **Stuart L Johnson:** Data curation; formal analysis; validation; investigation; methodology. **Kevin P Legan:** Investigation. **Corné J Kros:** Methodology; writing – review and editing. **Guy P Richardson:** Investigation; methodology; writing – review and editing. **Mirna Mustapha:** Resources; funding acquisition; methodology; writing – review and editing. **Walter Marcotti:** Conceptualization; resources; data curation; formal analysis; supervision; funding acquisition; validation; investigation; methodology; writing – original draft.

Disclosure and competing interests statement

The authors declare that they have no conflict of interest.

References

Antonellis PJ, Pollock LM, Chou SW, Hassan A, Geng R, Chen X, Fuchs E, Alagramam KN, Auer M, McDermott BM Jr (2014) ACF7 Is a hair-bundle antecedent, positioned to integrate cuticular plate Actin and somatic tubulin. *J Neurosci* 34: 305–312

Ashique AM, Choe Y, Karlen M, May SR, Phamluong K, Solloway MJ, Ericson J, Peterson AS (2009) The Rfx4 transcription factor modulates Shh signaling by regional control of ciliogenesis. *Sci Signal* 2: ra70

Bading H, Ginty DD, Greenberg ME (1993) Regulation of gene expression in hippocampal neurons by distinct calcium signaling pathways. *Science* 260: 181–186

Baron U, Bujard H (2000) Tet repressor-based system for regulated gene expression in eukaryotic cells: principles and advances. *Methods Enzymol* 327: 401–421

Belyantseva IA, Boger ET, Naz S, Frolenkov GI, Sellers JR, Ahmed ZM, Griffith AJ, Friedman TB (2005) Myosin-XVa is required for tip localization of whirlin and differential elongation of hair-cell stereocilia. *Nat Cell Biol* 7: 148–156

© 2023 The Authors

Bermingham NA, Hassan BA, Price SD, Vollrath MA, Ben-Arie N, Eatock RA, Bellen HJ, Lysakowski A, Zoghbi HY (1999) Math1: an essential gene for the generation of inner ear hair cells. *Science* 284: 1837 – 1841

Berridge MJ, Lipp P, Bootman MD (2000) The versatility and universality of calcium signalling. *Nat Rev Mol Cell Biol* 1: 11 – 21

Beutner D, Moser T (2001) The presynaptic function of mouse cochlear inner hair cells during development of hearing. *J Neurosci* 21: 4593 – 4599

Blankenship AG, Feller MB (2010) Mechanisms underlying spontaneous patterned activity in developing neural circuits. *Nat Rev Neurosci* 11: 18 – 29

Brandt N, Kuhn S, Münkner S, Braig C, Winter H, Blin N, Vonthein R, Knipper M, Engel J (2007) Thyroid hormone deficiency affects postnatal spiking activity and expression of Ca^{2+} and K^{+} channels in rodent inner hair cells. *J Neurosci* 27: 3174 – 3186

Buglo E, Sarmiento E, Martuscelli NB, Sant DW, Danzi MC, Abrams AJ, Dallman JE, Züchner S (2020) Genetic compensation in a stable slc25a46 mutant zebrafish: a case for using F0 CRISPR mutagenesis to study phenotypes caused by inherited disease. *PLoS One* 15: e0230566

Carlton AJ, Halford J, Underhill A, Jeng JY, Avenarius MR, Gilbert ML, Ceriani F, Ebisine K, Brown SDM, Bowl MR *et al* (2021) Loss of Baiap2l2 destabilizes the transducing stereocilia of cochlear hair cells and leads to deafness. *J Physiol* 599: 1173 – 1198

Ceriani F, Hendry A, Jeng JY, Johnson SL, Stephani F, Olt J, Holley MC, Mammano F, Engel J, Kros CJ *et al* (2019) Coordinated calcium signalling in cochlear sensory and non-sensory cells refines afferent innervation of outer hair cells. *EMBO J* 38: e99839

Chessum L, Matern MS, Kelly MC, Johnson SL, Ogawa Y, Milon B, McMurray M, Driver EC, Parker A, Song Y *et al* (2018) Helios is a key transcriptional regulator of outer hair cell maturation. *Nature* 563: 696 – 700

Clause A, Kim G, Sonntag M, Weisz CJ, Vetter DE, Rűbsamen R, Kandler K (2014) The precise temporal pattern of prehearing spontaneous activity is necessary for tonotopic map refinement. *Neuron* 82: 822 – 835

Clause A, Lauer AM, Kandler K (2017) Mice lacking the Alpha9 subunit of the nicotinic acetylcholine receptor exhibit deficits in frequency difference limens and sound localization. *Front Cell Neurosci* 11: 167

Corns LF, Johnson SL, Kros CJ, Marcotti W (2014) Calcium entry into stereocilia drives adaptation of the mechanoelectrical transducer current of mammalian cochlear hair cells. *Proc Natl Acad Sci U S A* 111: 14918 – 14923

Corns LF, Johnson SL, Roberts T, Ranatunga KM, Hendry A, Ceriani F, Safieddine S, Steel KP, Forge A, Petit C *et al* (2018) Mechanotransduction is required for establishing and maintaining mature inner hair cells and regulating efferent innervation. *Nat Commun* 9: 4015

Delprat B, Michel V, Goodyear R, Yamasaki Y, Michalski N, El-Amraoui A, Perfettini I, Legrain P, Richardson G, Hardelin JP *et al* (2005) Myosin XVa and whirlin, two deafness gene products required for hair bundle growth, are located at the stereocilia tips and interact directly. *Hum Mol Genet* 14: 401 – 410

Dolmetsch RE, Lewis RS, Goodnow CC, Healy JI (1997) Differential activation of transcription factors induced by Ca^{2+} response amplitude and duration. *Nature* 386: 855 – 858

Eckrich T, Blum K, Milenkovic I, Engel J (2018) Fast Ca^{2+} transients of inner hair cells Arise coupled and uncoupled to Ca^{2+} waves of inner supporting cells in the developing mouse cochlea. *Front Mol Neurosci* 11: 264

Ehret G (1983) Development of hearing and response behavior to sound stimuli, behavioral studies. In *Development of the Auditory and Vestibular Systems*, Romand R (ed), pp 211 – 237. New York, NY: Academic Press

El-Brolosy MA, Stainier DYR (2017) Genetic compensation: a phenomenon in search of mechanisms. *PLoS Genet* 13: e1006780

Elkon R, Milon B, Morrison L, Shah M, Vijayakumar S, Racherla M, Leitch CC, Silipino L, Hadi S, Weiss-Gayet M *et al* (2015) RFX transcription factors are essential for hearing in mice. *Nat Commun* 6: 8549

Ewels PA, Peltzer A, Fillinger S, Patel H, Alneberg J, Wilm A, Garcia MU, Di Tommaso P, Nahnsen S (2020) The nf-core framework for community-curated bioinformatics pipelines. *Nat Biotechnol* 38: 276 – 278

Fields RD, Lee PR, Cohen JE (2005) Temporal integration of intracellular Ca^{2+} signaling networks in regulating gene expression by action potentials. *Cell Calcium* 37: 433 – 442

Fuchs PA (2005) Time and intensity coding at the hair cell's ribbon synapse. *J Physiol* 566: 7 – 12

Furness DN, Hackney CM (1986) High-resolution scanning-electron microscopy of stereocilia using the osmium-thiocarbohydrazide coating technique. *Hear Res* 21: 243 – 249

Furness DN, Mahendrasingam S, Ohashi M, Fettiplace R, Hackney CM (2008) The dimensions and composition of stereociliary rootlets in mammalian cochlear hair cells: comparison between high and low-frequency cells and evidence for a connection to the lateral membrane. *J Neurosci* 28: 6342 – 6353

Gale JE, Marcotti W, Kennedy HJ, Kros CJ, Richardson GP (2001) FM1-43 dye behaves as a permeant blocker of the hair-cell mechanotransducer channel. *J Neurosci* 21: 7013 – 7025

García-Añoveros J, Clancy JC, Foo CZ, García-Gómez I, Zhou Y, Homma K, Cheatham MA, Duggan A (2022) Tbx2 is a master regulator of inner versus outer hair cell differentiation. *Nature* 605: 298 – 303

Gillespie M, Jassal B, Stephan R, Milacic M, Rothfels K, Senff-Ribeiro A, Griss J, Sevilla C, Matthews L, Gong C *et al* (2022) The reactome pathway knowledgebase 2022. *Nucleic Acids Res* 50: D687 – D692

Glowatzki E, Fuchs PA (2000) Cholinergic synaptic inhibition of inner hair cells in the neonatal mammalian cochlea. *Science* 288: 2366 – 2368

Goodyear RJ, Legan PK, Wright MB, MarcottiW OA, Coats SA, Booth CJ, Kros CJ, Seifert RA, Bowen-Pope DF, Richardson GP (2003) A receptor-like inositol lipid phosphatase is required for the maturation of developing cochlear hair bundles. *J Neurosci* 23: 9208 – 9219

Grubb MS, Thompson ID (2004) The influence of early experience on the development of sensory systems. *Curr Opin Neurobiol* 14: 503 – 512

Hagenston AM, Bading H (2011) Calcium signaling in synapse-to-nucleus communication. *Cold Spring Harb Perspect Biol* 3: a004564

Heinz S, Benner C, Spann N, Bertolino E, Lin YC, Laslo P, Cheng JX, Murre C, Singh H, Glass CK (2010) Simple combinations of lineage-determining transcription factors prime cis-regulatory elements required for macrophage and B cell identities. *Mol Cell* 38: 576 – 589

Hertzano R, Shalit E, Rzadzinska AK, Dror AA, Song L, Ron U, Tan JT, Shitrit AS, Fuchs H, Hasson T *et al* (2008) A Myo6 mutation destroys coordination between the myosin heads, revealing new functions of myosin VI in the stereocilia of mammalian inner ear hair cells. *PLoS Genet* 4: e1000207

Jaeger RG, Fex J, Kachar B (1994) Structural basis for mechanical transduction in the frog vestibular sensory apparatus: II. The role of microtubules in the organization of the cuticular plate. *Hear Res* 77: 207 – 215

Jeng JY, Ceriani F, Olt J, Brown SDM, HolleyMC BMR, Johnson SL, Marcotti W (2020) Pathophysiological changes in inner hair cell ribbon synapses in the ageing mammalian cochlea. *J Physiol* 598: 4339 – 4355

Johnson SL, Marcotti W, Kros CJ (2005) Increase in efficiency and reduction in Ca^{2+} dependence of exocytosis during development of mouse inner hair cells. *J Physiol* 563: 177 – 191

Johnson SL, Adelman JP, Marcotti W (2007) Genetic deletion of SK2 channels in mouse inner hair cells prevents the developmental linearization in the Ca^{2+} dependence of exocytosis. *J Physiol* 583: 631 – 646

 © 2023 The Authors

Johnson SL, Eckrich T, Kuhn S, Zampini V, Franz C, Ranatunga KM, Roberts TP, Masetto S, Knipper M, Kros CJ *et al* (2011) Position-dependent patterning of spontaneous action potentials in immature cochlear inner hair cells. *Nat Neurosci* 14: 711–717

Johnson SL, Kennedy HJ, Holley MC, Fettiplace R, Marcotti W (2012) The resting transducer current drives spontaneous activity in prehearing mammalian cochlear inner hair cells. *J Neurosci* 32: 10479–10483

Johnson SL, Kuhn S, Franz C, Ingham N, Furness DN, Knipper M, Steel KP, Adelman JP, Holley MC, Marcotti W (2013) Presynaptic maturation in auditory hair cells requires a critical period of sensory-independent spiking activity. *Proc Natl Acad Sci U S A* 110: 8720–8725

Johnson SL, Ceriani F, Houston O, Polishchuk R, Polishchuk E, Crispino G, Zorzi V, Mammano F, Marcotti W (2017) Connexin-mediated signaling in nonsensory cells is crucial for the development of sensory inner hair cells in the mouse cochlea. *J Neurosci* 37: 258–268

Jones TA, Leake PA, Snyder RL, Stakhovskaya O, Bonham B (2007) Spontaneous discharge patterns in cochlear spiral ganglion cells before the onset of hearing in cats. *J Neurophysiol* 98: 1898–1908

Katz LC, Shatz CJ (1996) Synaptic activity and the construction of cortical circuits. *Science* 274: 1133–1138

Khilan AA, Al-Maslamani NA, Horn HF (2021) Cell stretchers and the LINC complex in mechanotransduction. *Arch Biochem Biophys* 702: 108829

Kitajiri S, Sakamoto T, Belyantseva IA, Goodyear RJ, Stepanyan R, Fujiwara I, Bird JE, Riazuddin S, Riazuddin S, Ahmed ZM *et al* (2010) Actin-bundling protein TRIOBP forms resilient rootlets of hair cell stereocilia essential for hearing. *Cell* 141: 786–798

Kros CJ, Ruppersberg JP, Rüsch A (1998) Expression of a potassium current in inner hair cells during development of hearing in mice. *Nature* 394: 281–284

Kuhn S, Johnson SL, Furness DN, Chen J, Ingham N, Hilton JM, Steffes G, Lewis MA, Zampini V, Hackney CM *et al* (2011) miR-96 regulates the progression of differentiation in mammalian cochlear inner and outer hair cells. *Proc Natl Acad Sci U S A* 108: 2355–2360

Lemeille S, Paschaki M, Baas D, Morlé L, Duteyrat JL, Ait-Lounis A, Barras E, Soulavie F, Jerber J, Thomas J *et al* (2020) Interplay of RFX transcription factors 1, 2 and 3 in motile ciliogenesis. *Nucleic Acids Res* 48: 9019–9036

Li Y, Zheng M, Lau YF (2014) The sex-determining factors SRY and SOX9 regulate similar target genes and promote testis cord formation during testicular differentiation. *Cell Rep* 8: 723–733

Lippe WR (1994) Rhythmic spontaneous activity in the developing avian auditory system. *J Neurosci* 14: 1486–1495

Liu Y, Qi J, Chen X, Tang M, Chu C, Zhu W, Li H, Tian C, Yang G, Zhong C *et al* (2019) Critical role of spectrin in hearing development and deafness. *Sci Adv* 5: eaav7803

Love MI, Huber W, Anders S (2014) Moderated estimation of fold change and dispersion for RNA-seq data with DESeq2. *Genome Biol* 15: 550

Manor U, Disanza A, Grati M, Andrade L, Lin H, Di Fiore PP, Scita G, Kachar B (2011) Regulation of stereocilia length by myosin XVa and whirlin depends on the Actin-regulatory protein Eps8. *Curr Biol* 21: 167–172

Marcotti W, Géléoc GS, Lennan GW, Kros CJ (1999) Transient expression of an inwardly rectifying potassium conductance in developing inner and outer hair cells along the mouse cochlea. *Pflugers Arch* 439: 113–122

Marcotti W, Johnson SL, Rüsch A, Kros CJ (2003a) Sodium and calcium currents shape action potentials in immature mouse inner hair cells. *J Physiol* 552: 743–761

Marcotti W, Johnson SL, Holley MC, Kros CJ (2003b) Developmental changes in the expression of potassium currents of embryonic, neonatal and mature mouse inner hair cells. *J Physiol* 548: 383–400

Marcotti W, Johnson SL, Kros CJ (2004) A transiently expressed SK current sustains and modulates action potential activity in immature mouse inner hair cells. *J Physiol* 557: 613–633

Maul A, Huebner AK, Strenzke N, Moser T, Rübsamen R, Jovanovic S, Hübner CA (2022) The Cl^--channel TMEM16A is involved in the generation of cochlear Ca^{2+} waves and promotes the refinement of auditory brainstem networks in mice. *Elife* 11: e72251

Mhatre AN, Li Y, Atkin G, Maghnouj A, Lalwani AK (2006) Expression of Myh9 in the mammalian cochlea: localization within the stereocilia. *J Neurosci Res* 84: 809–818

Mikaelian D, Ruben RJ (1964) Development of hearing in the normal CBA-J mouse: correlation of physiological observations with behavioural responses and with cochlear anatomy. *Arch Otolaryngol* 59: 451–461

Moody WJ, Bosma MM (2005) Ion channel development, spontaneous activity, and activity-dependent development in nerve and muscle cells. *Physiol Rev* 85: 883–941

Moser T, Grabner CP, Schmitz F (2020) Sensory processing at ribbon synapses in the retina and the cochlea. *Physiol Rev* 100: 103–144

Müller M, von Hünerbein K, Hoidis S, Smolders JW (2005) A physiological place-frequency map of the cochlea in the CBA/J mouse. *Hear Res* 202: 63–73

Müller NIC, Sonntag M, Maraslioglu A, Hirtz JJ, Friauf E (2019) Topographic map refinement and synaptic strengthening of a sound localization circuit require spontaneous peripheral activity. *J Physiol* 597: 5469–5493

Ohba S, He X, Hojo H, McMahon AP (2015) Distinct transcriptional programs underlie Sox9 regulation of the mammalian chondrocyte. *Cell Rep* 12: 229–243

Pacentine I, Chatterjee P, Barr-Gillespie PG (2020) Stereocilia rootlets: actin-based structures that are essential for structural stability of the hair bundle. *Int J Mol Sci* 21: 324

Patro R, Duggal G, Love MI, Irizarry RA, Kingsford C (2017) Salmon provides fast and bias-aware quantification of transcript expression. *Nat Methods* 14: 417–419

Romand R (1983) Development of the cochlea. In *Development of the auditory and vestibular systems*, Romand R (ed), pp 47–88. New York, NY: Academic Press

Roux I, Safieddine S, Nouvian R, Grati M, Simmler MC, Bahloul A, Perfettini I, Le Gall M, Rostaing P, Hamard G *et al* (2006) Otoferlin, defective in a human deafness form, is essential for exocytosis at the auditory ribbon synapse. *Cell* 127: 277–289

Self T, Sobe T, Copeland NG, Jenkins NA, Avraham KB, Steel KP (1999) Role of myosin VI in the differentiation of cochlear hair cells. *Dev Biol* 214: 331–341

Sonntag M, Englitz B, Kopp-Scheinpflug C, Rübsamen R (2009) Early postnatal development of spontaneous and acoustically evoked discharge activity of principal cells of the medial nucleus of the trapezoid body: an *in vivo* study in mice. *J Neurosci* 29: 9510–9520

Stellwagen D, Shatz CJ (2002) An instructive role for retinal waves in the development of retinogeniculate connectivity. *Neuron* 33: 357–367

Tritsch NX, Yi E, Gale JE, Glowatzki E, Bergles DE (2007) 2007 the origin of spontaneous activity in the developing auditory system. *Nature* 450: 50–55

Tritsch NX, Rodríguez-Contreras A, Crins TT, Wang HC, Borst JG, Bergles DE (2010) Calcium action potentials in hair cells pattern auditory neuron activity before hearing onset. *Nat Neurosci* 13: 1050–1052

Wang HC, Lin CC, Cheung R, Zhang-Hooks Y, Agarwal A, Ellis-Davies G, Rock J, Bergles DE (2015) Spontaneous activity of Cochlear hair cells triggered by fluid secretion mechanism in adjacent support cells. *Cell* 163: 1348–1359

© 2023 The Authors

Wangemann P, Schacht J (1996) Homeostatic mechanisms in the cochlea. In *The cochlea*, Dallos P, Popper A, Fay R (eds), pp 130 – 185. New York, NY: Springer

Yu CR, Power J, Barnea G, O'Donnell S, Brown HE, Osborne J, Axel R, Gogos JA (2004) Spontaneous neural activity is required for the establishment and maintenance of the olfactory sensory map. *Neuron* 42: 553 – 566

Zampini V, Rüttiger L, Johnson SL, Franz C, Furness DN, Waldhaus J, Xiong H, Hackney CM, Offenhauser N, Di Fiore PP *et al* (2011) Eps8 regulates hair bundle length and functional maturation of mammalian auditory hair cells. *PLoS Biol* 9: e1001048

Zhang LI, Poo MM (2001) Electrical activity and development of neural circuits. *Nat Neurosci* 4: 1207 – 1214

Zhang-Hooks Y, Agarwal A, Mishina M, Bergles DE (2016) NMDA receptors enhance spontaneous activity and promote neuronal survival in the developing cochlea. *Neuron* 89: 337 – 350

Zhou Y, Zhou B, Pache L, Chang M, Khodabakhshi AH, Tanaseichuk O, Benner C, Chanda SK (2019) Metascape provides a biologist-oriented resource for the analysis of systems-level datasets. *Nat Commun* 10: 1523

License: This is an open access article under the terms of the Creative Commons Attribution License, which permits use, distribution and reproduction in any medium, provided the original work is properly cited.

© 2023 The Authors

Expanded View Figures

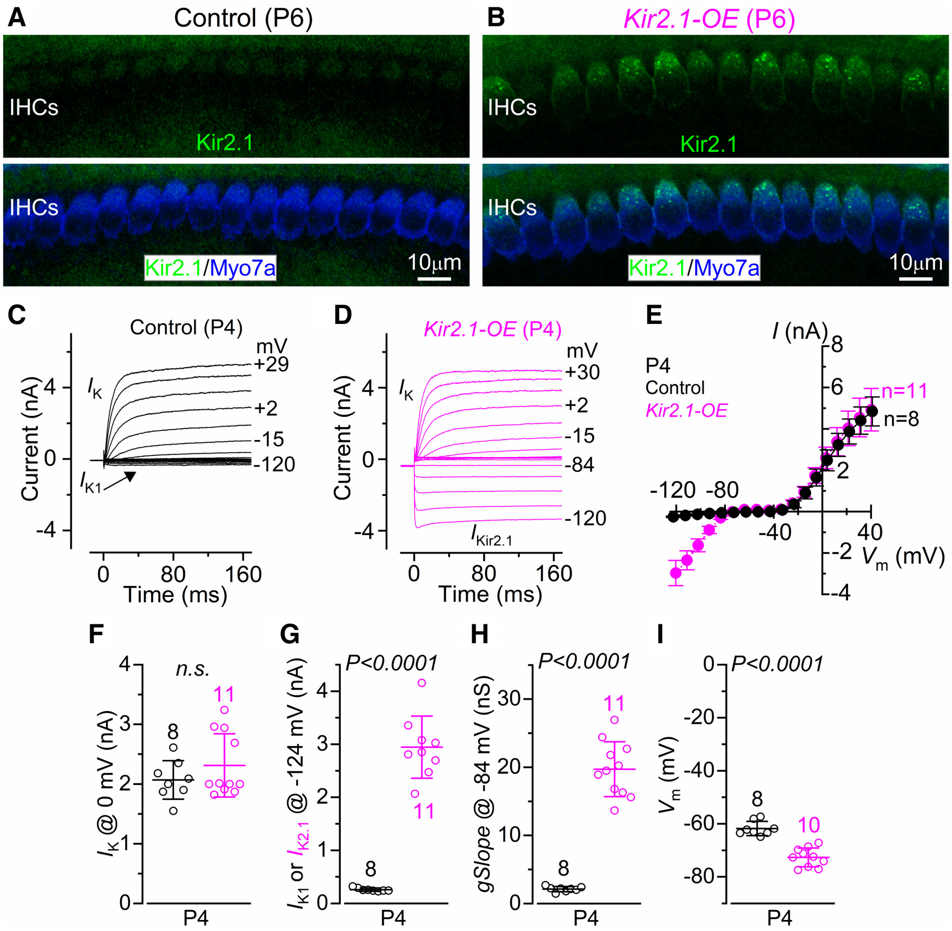

Figure EV1. Basolateral membrane properties of P4 IHCs overexpressing Kir2.1 channels.

A, B Maximum intensity projections of confocal *z*-stacks showing the inner hair cells (IHCs) of the apical cochlear region from control (A) and littermate *Kir2.1* overexpressing (B, *Kir2.1*-OE) mice at postnatal day 6 (P6). IHCs were stained with antibodies against Kir2.1 (green) and the hair cell marker Myo7a (blue). At least 3 mice for each genotype were used. Scale bars: 10 μm.

C, D Currents from IHCs of control (C) and *Kir2.1*-OE (D) prehearing P4 mice. Currents were elicited by using depolarizing and hyperpolarizing voltage steps (10 mV nominal increment), from a holding potential of −84 mV. Test potentials are shown next to some of the traces. Note that the large inward rectifier Kir2.1 current is only present in the IHCs of the *Kir2.1*-OE mouse (D). The outward current is primarily carried by a delayed rectifier current I_K. I_{K1} identifies the small inwardly rectifying K^+ current normally expressed in immature IHCs.

E Steady-state current–voltage curves obtained from IHCs of control and *Kir2.1*-OE P4 mice.

F, G Size of the total steady-state outward (F, I_K: Control 2.07 ± 0.32 nA, $n = 8$; *Kir2.1*-OE 2.31 ± 0.53 nA, $n = 11$) and inward (G, Control, I_{K1}: 0.26 ± 0.03 nA, $n = 8$; *Kir2.1*-OE, $I_{Kir2.1}$: 2.97 ± 0.61 nA, $n = 11$) K^+ currents from P4 IHCs measured at 0 mV and −124 mV, respectively. n.s: $P = 0.2836$.

H Slope conductance of the current measured at −84 mV (Control 2.12 ± 0.38 nS, $n = 8$; *Kir2.1*-OE 19.71 ± 4.03 nA, $n = 11$).

I Resting membrane potential (V_m) measured in IHCs from control (−61.8 ± 2.7 mV, $n = 8$) and *Kir2.1*-OE (−72.7 ± 3.6 mV, $n = 10$).

Data information: In panels F–I, data are shown as means ± SD, and the single cell value recordings (open symbols) are plotted with the average data. The number of IHCs investigated is shown above the average data points. All statistical tests were performed using the Student's *t*-test.
Source data are available online for this figure.

Figure EV2. The morphology and function of the synaptic machinery in IHCs are not affected by Kir2.1 overexpression.

A, B Maximum intensity projections of confocal *z*-stacks of IHCs taken from the apical cochlear region (9–12 kHz) of control (A) and *Kir2.1*-OE (B) mice at P11 using antibodies against CtBP2 (ribbon synaptic marker: red) and GluR2 (postsynaptic receptor marker: green). Myosin 7a (Myo7a) was used as the IHC marker (blue). At least 3 mice for each genotype were used. Scale bar 10 μm.

C, D Number of CtBP2 and GluR2 puncta in IHCs from control and *Kir2.1*-OE (C) and co-localized CtBP2 and GluR2 puncta in IHCs from both genotypes (D). Data are plotted as mean values (lines) and individual CtBP2 and GluR2 counts or colocalized counts (smaller open symbols). Numbers above or below the data in panels represent the IHCs (and mice) used for each time point. All comparisons were not significantly different, and *P*-values are shown below the data and were obtained using the Student's *t*-test. Average values are mean ± SD.

E, F Calcium current (I_{Ca}) and corresponding changes in membrane capacitance (ΔC_m) recorded from IHCs of control and *Kir2.1-OE* mice. Recordings were obtained in response to 50 ms voltage steps (10 mV increments) from −81 mV and using 1.3 mM extracellular Ca^{2+} and at body temperature. For clarity, only maximal responses at −11 mV are shown. Average peak I_{Ca} (bottom) and ΔC_m (top) curves from IHCs of control (black) and *Kir2.1-OE* (magenta) mice (F). Average values are mean ± SD.

Source data are available online for this figure.

 © 2023 The Authors

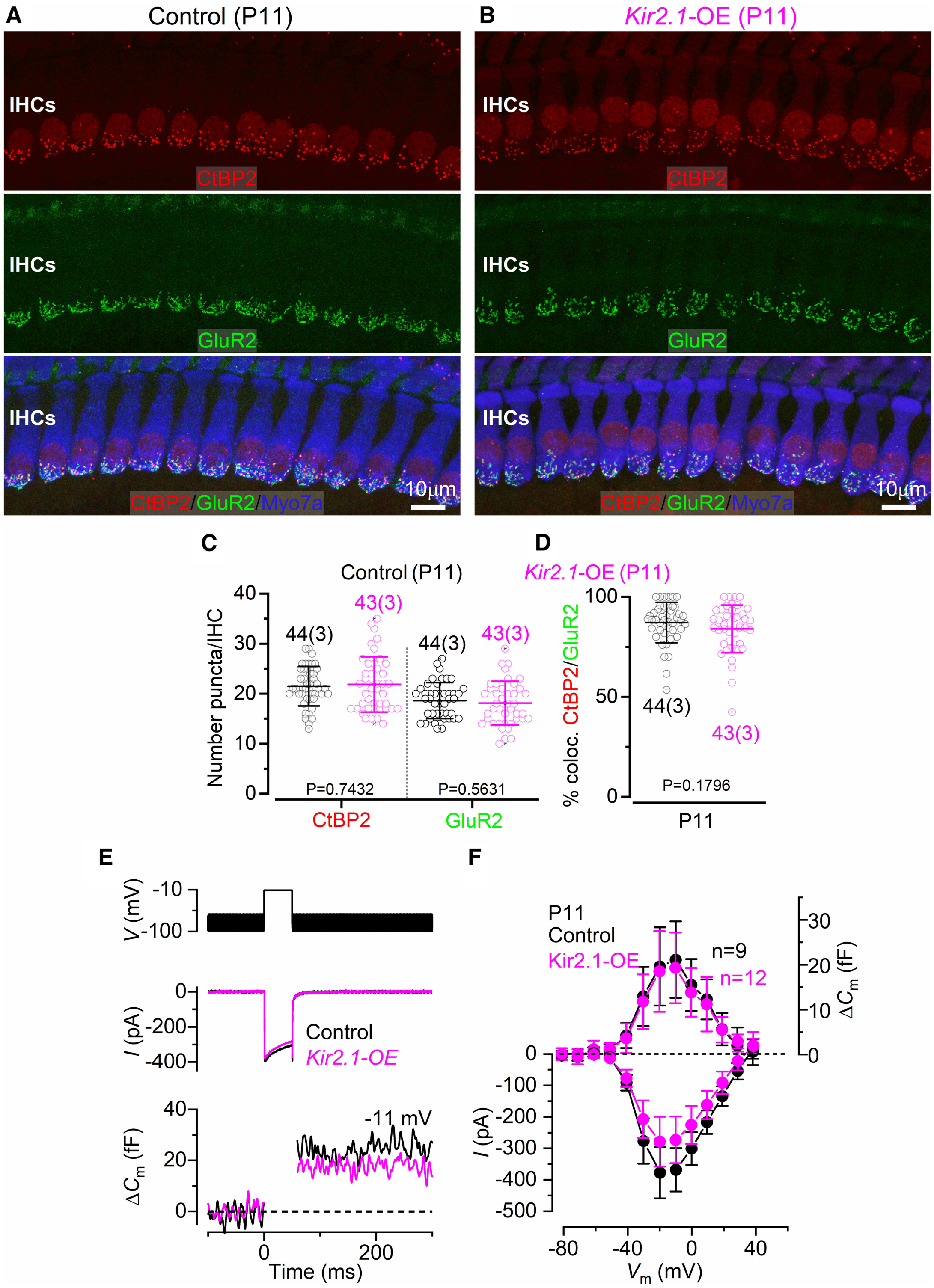

A
Control (P11)
B
Kir2.1-OE (P11)
IHCs
CtBP2
GluR2
CtBP2/GluR2/Myo7a
10µm
C
D
Number puncta/IHC
44(3)
43(3)
P=0.7432
P=0.5631
CtBP2
GluR2
% coloc. CtBP2/GluR2
P=0.1796
P11
E
V (mV)
I (pA)
ΔCm (fF)
Control
Kir2.1-OE
-11 mV
Time (ms)
F
P11
n=9
n=12
Vm (mV)

Figure EV2.

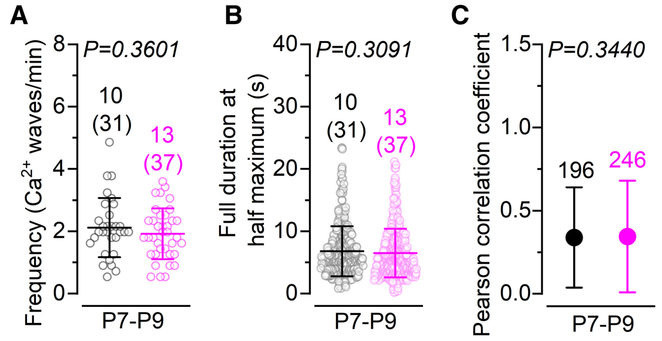

Figure EV3. Ca^{2+} signals in IHCs are correlated with the Ca^{2+} waves in nonsensory cells.

A, B Frequency (A) and full duration at half maximum (B) of the Ca^{2+} waves recorded from P7-P9 control and *Kir2.1*-OE mice. The full duration at half maximum measures how long the Ca^{2+} waves last in time, which was measured as the duration of the Ca^{2+} trace at half the peak. Data are shown as means ± SD and single data points are shown as open circles. The number above the data represents the number of mice tested and, in between the brackets, the number of recordings. Note that for the frequency (A) each data point represents one recording (usually 2–3 recordings per mouse). For the full duration at half maximum (B), we plotted one data point for each of the Ca^{2+} waves recorded from both control (365 Ca^{2+} waves) and *Kir2.1*-OE (395 Ca^{2+} waves) mice. *P*-values shown above the panels were calculated using the Student's *t*-test.

C Average Pearson correlation coefficient between the Ca^{2+} activity in individual IHCs and the Ca^{2+} waves in the nonsensory cells from control and *Kir2.1-OE* mice (10 and 13, respectively). Numbers of IHCs analyzed are shown above the data. Data are shown as means ± SD. The correlation coefficients for both control and *Kir2.1-OE* mice were significantly different from zero ($P < 0.001$, Wilcoxon rank-sum test). There was no significant difference between control and *Kir2.1-OE* mice ($P = 0.3440$, Mann–Whitney U test). For calculating descriptive statistics and tests of significance, Pearson correlation coefficients were transformed using Fisher transformation (inverse hyperbolic tangent).

Source data are available online for this figure.

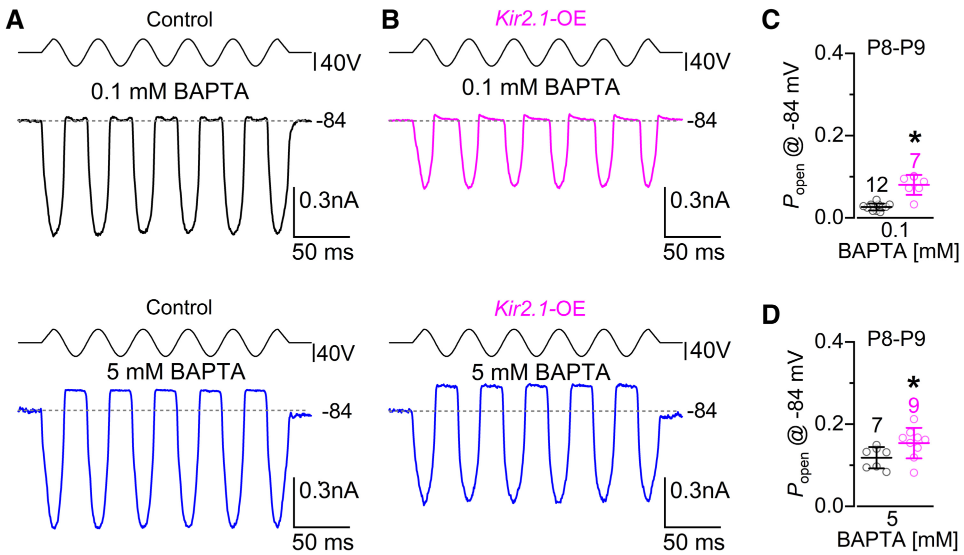

Figure EV4. Calcium sensitivity of the MET current is reduced in Kir2.1 overexpressing IHCs at the start of the second postnatal week.

A, B Saturating MET currents recorded from apical IHCs of P8 control (A) and *Kir2.1*-OE (B) mice in the presence of 0.1 mM (upper panels) and 5 mM (lower panels) of the fast Ca^{2+} chelator BAPTA. Responses were obtained in response to 50 Hz sinusoidal force stimuli to the hair bundles at membrane potentials of −84 mV. Driver voltage (DV) stimuli to the fluid jet are shown above the traces, with positive deflections of the DV being excitatory.

C, D Resting open probability (P_{open}) of the MET current in IHCs from the two genotypes (control: black; *Kir2.1*-OE: magenta) measured at −84 mV in 0.1 mM BAPTA (C) and 5 mM BAPTA (D). The P_{open} was found significantly different between the two genotypes (C: $^*P < 0.0001$; D: $^*P = 0.0496$, *t*-test). The increased resting P_{open} using a fixed BAPTA concentration indicates a reduced Ca^{2+} sensitivity of the MET channel in the IHCs of *Kir2.1*-OE mice. In panels C and D, data are shown as means ± SD, and the single cell value recordings (open symbols) are plotted behind the average data. The number of IHCs investigated is shown above the average data points from 5 control and 5 littermates *Kir2.1*-OE mice.

Source data are available online for this figure.

 © 2023 The Authors

A critical period of spontaneous spiking is required for hair-bundle maintenance in inner hair cells

Adam J. Carlton, Jing-Yi Jeng, Fiorella Grandi, Francesca De Faveri, Federico Ceriani, Lara De Tomasi, Anna Underhill, Stuart L. Johnson, Kevin Legan, Corné J. Kros, Guy P Richardson, Mirna Mustapha, Walter Marcotti

This document includes the following documents:

Appendix Figure S1
Appendix Figure S2
Appendix Figure S3

Appendix Figure S1

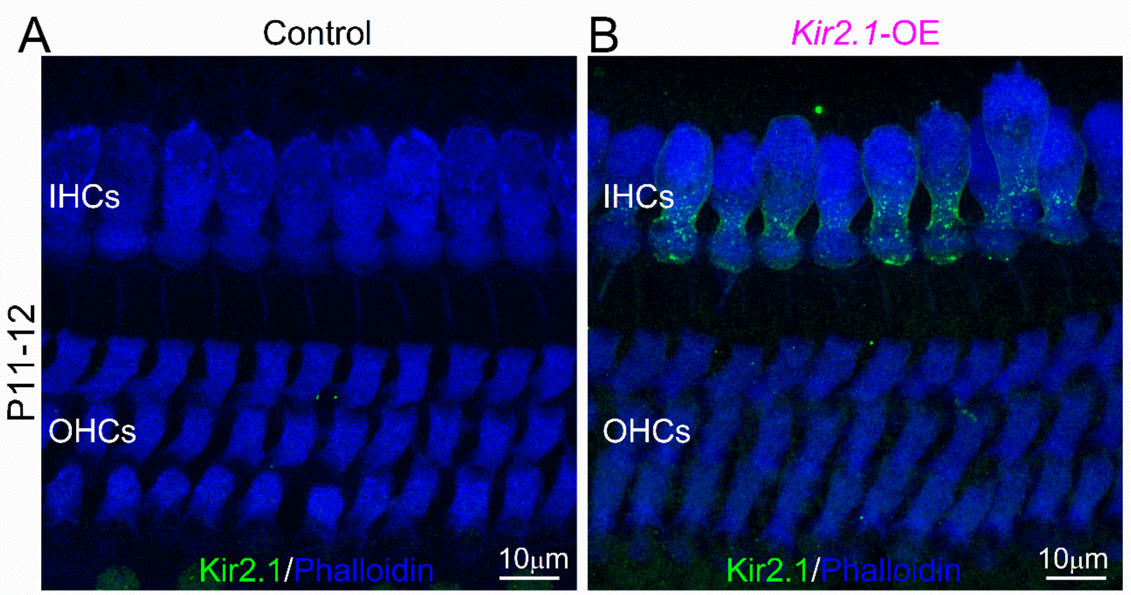

Appendix Figure S1: Outer hair cells do not express Kir2.1 channels.

A,B, Representative maximum intensity projections of confocal z-stacks showing the IHCs and OHCs of the apical cochlear region from control (**A**) and *Kir2.1*-OE (**B**) mice at postnatal day 11-P12. Hair cells were stained with antibodies against the K^+ channel Kir2.1 (green) and Phalloidin (blue: cell marker). Scale bars: 10 µm.

Appendix Figure S2

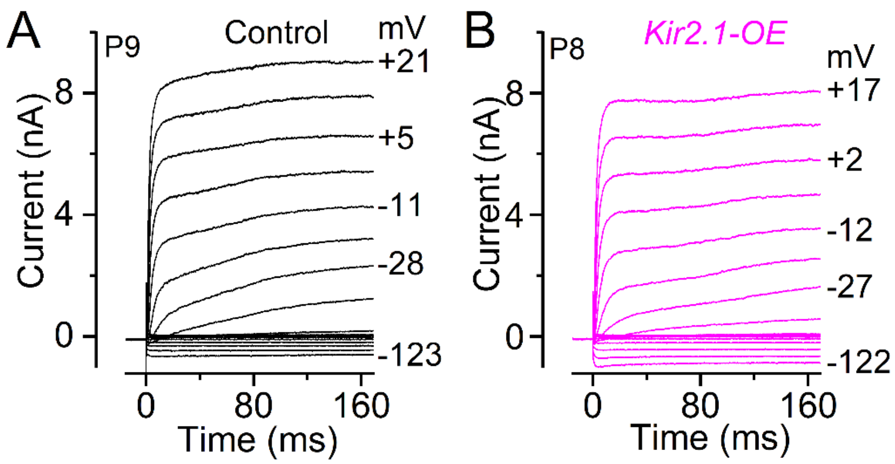

Appendix Figure S2. Basolateral membrane currents in the IHCs from mice in which DOX was removed at P5

A, B, Currents from IHCs of control (**A**, P9) and *Kir2.1*-OE (**B**, P8) pre-hearing mice; these recordings are from the same IHCs shown in **Fig. 7C,D**. Currents were elicited by using depolarizing and hyperpolarizing voltage steps, with a nominal increment of 10 mV, from a holding potential of −84 mV. Test potentials are shown next to some of the traces. Note the very similar current profile between the IHCs from both genotypes.

Appendix Figure S3

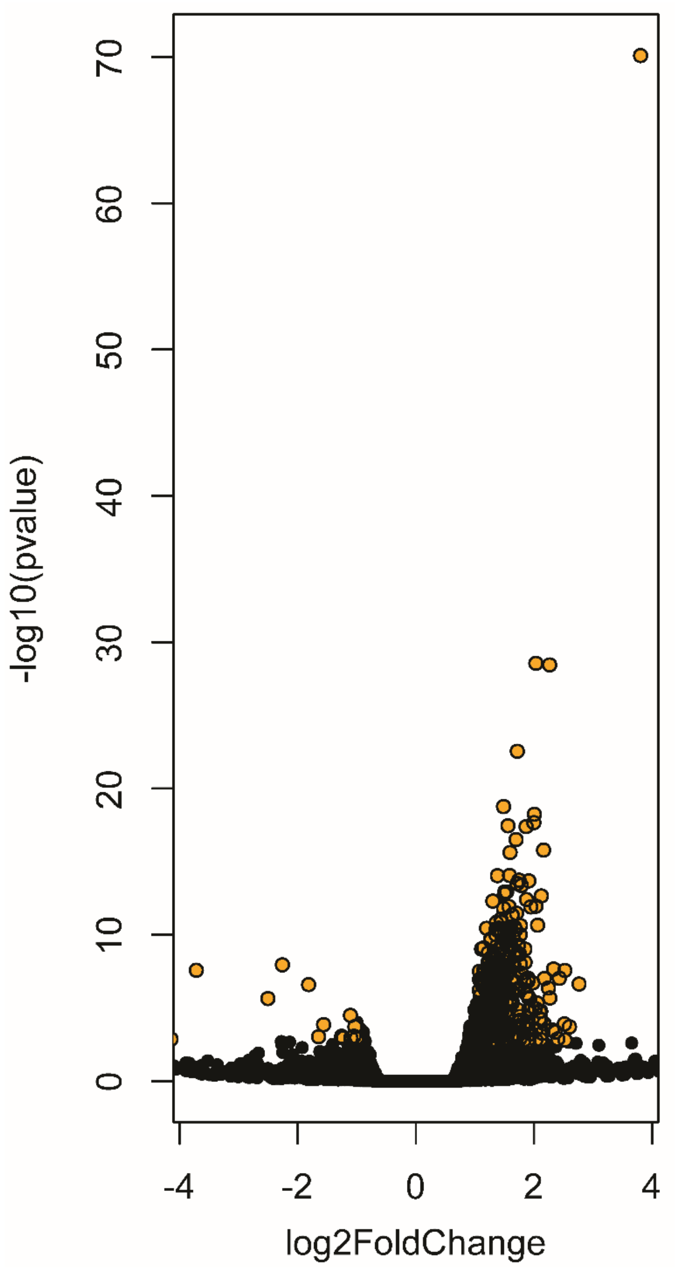

Appendix Figure S3. Volcano Plot of Differentially Expressed Genes in RNA-sequencing. Volcano plot of differentially expressed genes. Each point represents a gene. Points colored in yellow represent those that passed the differential expression analysis thresholds. The point at the highest value is *Kir2.1*.

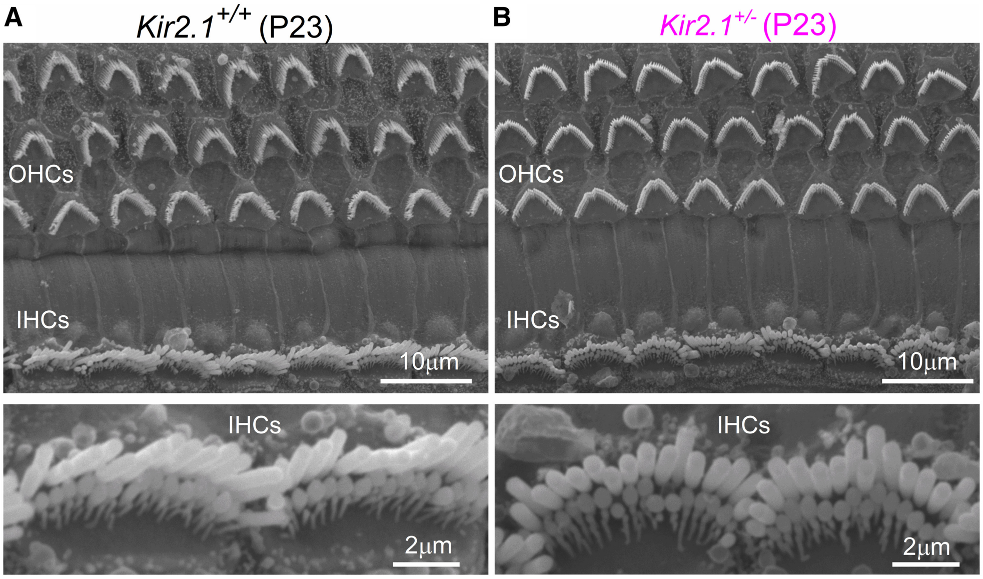

Figure EV5. IHC bundle morphology in Kir2.1 mice.

A, B Scanning electron micrographs showing the typical hair-bundle structure of apical-coil IHCs and OHCs in *Kir2.1* P23 mice (without pairing them with *Otof*$^{tTA+/−}$ mice) fed with DOX in the drinking water throughout their life. This result shows that the application of DOX to heterozygous *Kir2.1* mice (without crossing with *Otof*^{tTA} mice) does not affect the hair-bundle morphology in adult mice. At least 3 mice for each genotype were used.

Source data are available online for this figure.
