# Supplementary material for: A critical period of prehearing spontaneous Ca2+ spiking is required for hair‐bundle maintenance in inner hair cells
Source: EMBO J. 2023 Jan 3;42(4):e112118. doi: 10.15252/embj.2022112118 (PMC9929643; doi:10.15252/embj.2022112118)
Supplement: Supplementary file 7 — Source Data for Figure 1 [file EMBJ-42-e112118-s002.zip › Figure 1/Figure 1E-I.docx]

**Figure 1E**

| **Control** | | | |  | **Kir2.1-OE** | | | |
| --- | --- | --- | --- | --- | --- | --- | --- | --- |
| **Vm** | **IK** | **SD** | **N** |  | **Vm** | **IK** | **SD** | **N** |
| -123.64928 | -297.34582 | 49.92772 | 8 |  | -119.68481 | -3134.19796 | 1370.38336 | 6 |
| -113.7235 | -235.32451 | 37.55062 | 8 |  | -110.57291 | -2469.27683 | 1059.22834 | 6 |
| -103.80104 | -169.67924 | 26.98943 | 8 |  | -101.45954 | -1820.14484 | 757.93103 | 6 |
| -93.86011 | -120.06249 | 17.13591 | 8 |  | -92.41635 | -1119.71761 | 464.07085 | 6 |
| -83.90811 | -79.3846 | 11.22214 | 8 |  | -83.30203 | -480.88248 | 197.82362 | 6 |
| -73.94389 | -48.73332 | 10.33234 | 8 |  | -73.86664 | -90.89504 | 163.84228 | 6 |
| -63.96533 | -30.15575 | 9.76659 | 8 |  | -64.17511 | 119.51711 | 155.16842 | 6 |
| -53.98544 | -12.31203 | 7.87024 | 8 |  | -54.31359 | 212.01105 | 132.11069 | 6 |
| -44.00704 | 5.9158 | 7.40322 | 8 |  | -44.30044 | 205.19898 | 83.04408 | 6 |
| -34.09699 | 78.18165 | 49.04359 | 8 |  | -34.33155 | 246.3539 | 156.34337 | 6 |
| -24.40814 | 335.32094 | 154.41743 | 8 |  | -24.68596 | 516.31158 | 311.26679 | 6 |
| -15.13905 | 940.34058 | 319.77195 | 8 |  | -15.30556 | 965.24528 | 464.40312 | 6 |
| -6.31822 | 1925.08915 | 627.42566 | 8 |  | -6.2363 | 1633.95287 | 697.20513 | 6 |
| 2.21492 | 3153.12096 | 970.45429 | 8 |  | 2.56328 | 2485.51517 | 1009.86662 | 6 |
| 10.7686 | 4379.03433 | 1304.57448 | 8 |  | 11.31492 | 3357.99756 | 1296.2237 | 6 |
| 19.49043 | 5471.04355 | 1557.09546 | 8 |  | 20.16638 | 4168.62803 | 1533.96171 | 6 |
| 28.41591 | 6400.44839 | 1719.78039 | 8 |  | 29.13704 | 4873.42495 | 1580.67207 | 6 |
| 37.50085 | 7197.41646 | 1818.51973 | 8 |  | 38.10814 | 5577.73939 | 1600.47193 | 6 |

**Figure 1F-I**

| **Control** | | | |  | **Kir2.1-OE** | | | |
| --- | --- | --- | --- | --- | --- | --- | --- | --- |
| **Figure 1F** | **Figure 1G** | **Figure 1H** | **Figure 1I** |  | **Figure 1F** | **Figure 1G** | **Figure 1H** | **Figure 1I** |
| **IK,D** | **IK1** | **Vm** | **gSlope** |  | **IK,D** | **IKir2.1** | **Vm** | **gSlope** |
| 2.55 | 301 | -59.8 | 1.5 |  | 1.14 | 2152 | -74.5 | 24.1 |
| 3.18 | 254 | -61.7 | 1.8 |  | 3.5 | 3974 | -76.7 | 27.6 |
| 1.6 | 249 |  | 1.8 |  | 2.5 | 3318 |  | 8.7 |
| 1.74 | 305 | -58.5 | 2.3 |  | 1.46 | 1353 | -73.4 | 23.2 |
| 2.03 | 303 | -66.7 | 2.3 |  | 1.7 | 2779 | -75.2 | 34.2 |
| 4.3 | 372 | -64.2 | 1.7 |  | 3.2 | 5228 | -67.7 | 34.9 |
| 4.3 | 235 | -68.1 | 1.1 |  |  |  |  |  |
| 3.3 | 359 | -58.9 | 2.1 |  |  |  |  |  |
