# Supplementary material for: A critical period of prehearing spontaneous Ca2+ spiking is required for hair‐bundle maintenance in inner hair cells
Source: EMBO J. 2023 Jan 3;42(4):e112118. doi: 10.15252/embj.2022112118 (PMC9929643; doi:10.15252/embj.2022112118)
Supplement: Supplementary file 9 — Source Data for Figure 3 [file EMBJ-42-e112118-s011.zip › Figure 3/Figure 3C-E.docx]

**Figure 3C**

| **Control** | | | |  | **Kir2.1-OE** | | | |
| --- | --- | --- | --- | --- | --- | --- | --- | --- |
| **Vm** | **IK** | **SD** | **N** |  | **Vm** | **IK** | **SD** | **N** |
| -124 | -1011.59306 | 149.80107 | 12 |  | -124 | -895.99679 | 255.61644 | 7 |
| -104 | -788.87394 | 126.0294 | 12 |  | -104 | -627.66323 | 192.03242 | 7 |
| -84 | -586.29027 | 91.94906 | 12 |  | -84 | -483.81328 | 162.08423 | 7 |
| -64 | -432.50075 | 74.95755 | 12 |  | -64 | -402.27649 | 137.34988 | 7 |
| -44 | -292.21429 | 52.43334 | 12 |  | -44 | -283.98084 | 97.21288 | 7 |
| -24 | -167.19897 | 34.68912 | 12 |  | -24 | -162.37117 | 55.91269 | 7 |
| -4 | -50.92657 | 31.71077 | 12 |  | -4 | -52.63101 | 28.64657 | 7 |
| 16 | 70.30535 | 18.93075 | 12 |  | 16 | 84.7009 | 39.25515 | 7 |
| 36 | 204.47806 | 35.71707 | 12 |  | 36 | 240.85658 | 89.36552 | 7 |
| 56 | 372.30645 | 61.9262 | 12 |  | 56 | 424.55334 | 147.70809 | 7 |
| 76 | 562.80451 | 95.08155 | 12 |  | 76 | 641.49517 | 238.69724 | 7 |
| 96 | 771.28043 | 126.7183 | 12 |  | 96 | 862.27499 | 297.91627 | 7 |

**Figure 3D-E**

| **Control** | | | |  | **Kir2.1-OE** | | | |
| --- | --- | --- | --- | --- | --- | --- | --- | --- |
| **Figure 3D** | | **Figure 3E** | |  | **Figure 3D** | | **Figure 3E** | |
| **I_T_(-124mV)** | **I_T_(+96mV)** | **Po(-124mV)** | **Po(+96mV)** |  | **I_T_(-124mV)** | **I_T_(+96mV)** | **Po(-124mV)** | **Po(+96mV)** |
| 1014.25171 | 717.92603 | 0.0162 | 0.09387 |  | 865.83813 | 733.06482 | 0.03503 | 0.3026 |
| 1010.13184 | 949.93591 | 0.04286 | 0.16177 |  | 527.51587 | 415.86865 | -0.0127 | 0.08501 |
| 1005.5542 | 817.56592 | 0.00778 | 0.06848 |  | 1150.41089 | 1103.46484 | 0.05854 | 0.15876 |
| 740.6413 | 621.01233 | 0.05001 | 0.27966 |  | 1054.78931 | 929.31104 | 0.04787 | 0.06793 |
| 926.36108 | 787.5061 | 0.04365 | 0.14629 |  | 1194.8396 | 1325.68335 | 0.06583 | 0.06285 |
| 1239.21716 | 913.89978 | 0.02615 | 0.18122 |  | 623.12524 | 664.45239 | 0.02182 | 0.14347 |
| 871.58203 | 577.13831 | 0.02845 | 0.30466 |  | 855.4585 | 864.07983 | 0.07769 | 0.23917 |
| 947.41821 | 672.45483 | 0.02429 | 0.23087 |  |  |  |  |  |
| 1059.29057 | 803.55322 | 0.03773 | 0.3346 |  |  |  |  |  |
| 1142.65442 | 869.29321 | 0.03499 | 0.26435 |  |  |  |  |  |
| 1262.06175 | 900.89337 | 0.04319 | 0.15803 |  |  |  |  |  |
| 919.95239 | 624.18616 | 0.04108 | 0.1417 |  |  |  |  |  |
