# Supplementary material for: A critical period of prehearing spontaneous Ca2+ spiking is required for hair‐bundle maintenance in inner hair cells
Source: EMBO J. 2023 Jan 3;42(4):e112118. doi: 10.15252/embj.2022112118 (PMC9929643; doi:10.15252/embj.2022112118)
Supplement: Supplementary file 10 — Source Data for Figure 4 [file EMBJ-42-e112118-s013.zip › Figure 4/Figure 4C-E.docx]

**Figure 4C**

| **Control** | | | |  | **Kir2.1-OE** | | | |
| --- | --- | --- | --- | --- | --- | --- | --- | --- |
| **Vm** | **IK** | **SD** | **N** |  | **Vm** | **IK** | **SD** | **N** |
| -124 | -1228.82209 | 178.64103 | 17 |  | -124 | -779.47468 | 306.49682 | 24 |
| -104 | -962.35541 | 137.6972 | 17 |  | -104 | -612.27276 | 235.09711 | 24 |
| -84 | -715.43689 | 91.46156 | 17 |  | -84 | -459.25918 | 181.12023 | 24 |
| -64 | -522.18512 | 62.957 | 17 |  | -64 | -339.6398 | 140.11508 | 24 |
| -44 | -357.0956 | 47.23558 | 17 |  | -44 | -235.50333 | 97.52045 | 24 |
| -24 | -207.1676 | 25.23372 | 17 |  | -24 | -143.80994 | 67.99553 | 24 |
| -4 | -77.45526 | 24.1019 | 17 |  | -4 | -68.99219 | 63.32087 | 24 |
| 16 | 64.00549 | 21.77389 | 17 |  | 16 | 29.31271 | 43.67403 | 24 |
| 36 | 234.70141 | 41.48863 | 17 |  | 36 | 159.13818 | 60.44555 | 24 |
| 56 | 444.22026 | 75.55958 | 17 |  | 56 | 310.15351 | 126.72514 | 24 |
| 76 | 681.25161 | 117.22064 | 17 |  | 76 | 480.62299 | 191.6135 | 24 |
| 96 | 941.83001 | 149.69852 | 17 |  | 96 | 658.38507 | 266.87208 | 24 |

**Figure 4D-E**

| **Control** | | | |  | **Kir2.1-OE** | | | |
| --- | --- | --- | --- | --- | --- | --- | --- | --- |
| **Figure 4D** | | **Figure 4E** | |  | **Figure 4D** | | **Figure 4E** | |
| **I_T_(-124mV)** | **I_T_(+96mV)** | **Po(-124mV)** | **Po(+96mV)** |  | **I_T_(-124mV)** | **I_T_(+96mV)** | **Po(-124mV)** | **Po(+96mV)** |
| 1241.30237 | 647.98987 | 0.00856 | 0.18716 |  | 964.22825 | 695.29199 | 0.10768 | 0.25022 |
| 1318.14124 | 1031.05798 | 0.0178 | 0.21642 |  | 915.52729 | 687.51746 | 0.02235 | 0.12912 |
| 1617.05005 | 1133.34631 | 0.00519 | 0.23157 |  | 799.56049 | 653.6864 | 0.10466 | 0.3781 |
| 1155.09021 | 929.6416 | 0.01064 | 0.07363 |  | 1176.1982 | 829.82385 | 0.0358 | 0.12267 |
| 1208.87744 | 960.92212 | 0.02203 | 0.21254 |  | 1443.22701 | 1237.48755 | 0.00297 | 0.01958 |
| 1167.67871 | 1008.9873 | 0.03724 | 0.25696 |  | 1096.49646 | 822.75378 | 0.04884 | 0.27492 |
| 1463.57203 | 1089.98596 | 0.00898 | 0.10285 |  | 665.45266 | 544.2301 | 0.10756 | 0.36334 |
| 1107.78797 | 820.41418 | 0.01965 | 0.22095 |  | 421.14255 | 311.02502 | 0.02017 | 0.35473 |
| 1344.90967 | 1036.07178 | 0.03992 | 0.25508 |  | 1003.26526 | 823.97449 | 0.01103 | 0.1537 |
| 1257.019 | 1018.98193 | 0.02879 | 0.1605 |  | 872.49756 | 655.74634 | 0.02315 | 0.30295 |
| 1245.52414 | 937.09314 | 0.01894 | 0.12868 |  | 763.5498 | 886.6958 | 0.26003 | 0.1973 |
| 1181.64063 | 845.94727 | 0.02201 | 0.12679 |  | 711.1106 | 1083.72998 | 0.05457 | 0.09854 |
| 1381.02214 | 1205.85114 | 0.03151 | 0.17129 |  | 852.95996 | 1091.35913 | 0.14319 | 0.06426 |
| 1128.84032 | 823.47089 | 0.02723 | 0.12317 |  | 1428.22266 | 887.87842 | 0.02851 | 0.2921 |
| 1277.13013 | 1008.27026 | 0.02794 | 0.16411 |  | 688.47656 | 580.24084 | 0.04504 | 0.26283 |
| 1304.6502 | 806.59656 | 0.0428 | 0.31704 |  | 234.06982 | 96.72034 | 0.02642 | 0.46487 |
| 887.36979 | 706.48193 | 0.04387 | 0.44838 |  | 607.04549 | 534.26111 | 0.04764 | 0.60786 |
|  |  |  |  |  | 495.18406 | 449.40759 | 0.04511 | 0.5574 |
|  |  |  |  |  | 911.65597 | 687.93591 | 0.02842 | 0.27243 |
|  |  |  |  |  | 439.46838 | 436.06567 | 0.05385 | 0.60085 |
|  |  |  |  |  | 523.55957 | 504.5166 | 0.0464 | 0.34721 |
|  |  |  |  |  | 483.70361 | 449.52393 | 0.12564 | 0.55499 |
|  |  |  |  |  | 522.995 | 385.48792 | 0.08761 | 0.69651 |
|  |  |  |  |  | 519.85677 | 465.88135 | 0.04338 | 0.50531 |
