# Supplementary material for: A critical period of prehearing spontaneous Ca2+ spiking is required for hair‐bundle maintenance in inner hair cells
Source: EMBO J. 2023 Jan 3;42(4):e112118. doi: 10.15252/embj.2022112118 (PMC9929643; doi:10.15252/embj.2022112118)
Supplement: Supplementary file 10 — Source Data for Figure 4 [file EMBJ-42-e112118-s013.zip › Figure 4/Figure 4H-I.docx]

**Figure 4H**

| **Control** | | | |  | **Kir2.1-OE** | | | |
| --- | --- | --- | --- | --- | --- | --- | --- | --- |
| **Vm** | **IK** | **SD** | **N** |  | **Vm** | **IK** | **SD** | **N** |
| -124 | -715.63792 | 205.8158 | 16 |  | -124 | -74.15883 | 115.71913 | 9 |
| -104 | -545.5231 | 155.67693 | 16 |  | -104 | -59.97043 | 99.90909 | 9 |
| -84 | -407.13277 | 116.85475 | 16 |  | -84 | -50.07482 | 80.93244 | 9 |
| -64 | -295.16348 | 80.54203 | 16 |  | -64 | -41.93682 | 61.37115 | 9 |
| -44 | -203.59211 | 54.67077 | 16 |  | -44 | -32.25142 | 47.31981 | 9 |
| -24 | -115.17667 | 26.33938 | 15 |  | -24 | -18.84516 | 28.40235 | 9 |
| -4 | -34.94721 | 23.63469 | 15 |  | -4 | -4.59234 | 10.7815 | 9 |
| 16 | 64.29066 | 27.27799 | 15 |  | 16 | 14.72304 | 24.48358 | 9 |
| 36 | 193.58088 | 65.65269 | 16 |  | 36 | 38.44198 | 54.09533 | 9 |
| 56 | 352.28473 | 112.76965 | 16 |  | 56 | 69.32237 | 98.59359 | 9 |
| 76 | 522.02031 | 159.03209 | 15 |  | 76 | 94.08458 | 138.45895 | 9 |
| 96 | 690.06901 | 216.72356 | 15 |  | 96 | 140.40625 | 182.89549 | 8 |

**Figure 4I**

| **Control** | | **Kir2.1-OE** | | |
| --- | --- | --- | --- | --- |
| **Figure 4I** | | | | |
| **I_T_(-124mV)** | **I_T_(+96mV)** | | **I_T_(-124mV)** | **I_T_(+96mV)** |
| 1203.00291 | 1101.0741 | | 340.7796 | 467.93604 |
| 483.19482 | 681.55933 | | 53.91431 | 111.7959 |
| 421.65121 | 435.38403 | | 0 | 0 |
| 950.45901 | 1117.54272 | | 0 | 0 |
| 685.1196 | 685.88257 | | 0 | 0 |
| 575.38347 | 549.82507 | | 150.39063 | 351.44043 |
| 724.28375 | 759.6261 | | 122.34497 | 192.07764 |
| 604.2301 | 506.82007 | | 0 | 0 |
| 619.60858 | 272.31848 | | 0 | 0 |
| 676.69678 | 647.37952 | |  |  |
| 960.00162 | 909.01685 | |  |  |
| 773.31543 | 711.46655 | |  |  |
| 743.30647 | 611.26721 | |  |  |
| 914.00146 | 736.31299 | |  |  |
| 505.14221 | 385.6405 | |  |  |
| 610.80933 | 512.23755 | |  |  |
|  |  | |  |  |
