# Supplementary material for: A critical period of prehearing spontaneous Ca2+ spiking is required for hair‐bundle maintenance in inner hair cells
Source: EMBO J. 2023 Jan 3;42(4):e112118. doi: 10.15252/embj.2022112118 (PMC9929643; doi:10.15252/embj.2022112118)
Supplement: Supplementary file 13 — Source Data for Figure 7 [file EMBJ-42-e112118-s006.zip › Figure 7/Figure 7G.docx]

**Figure 7G**

| **Control** | |  | **Kir2.1-OE** | |
| --- | --- | --- | --- | --- |
| **1.3 mM Ca^2+^** | **0.3 mM Ca^2+^** |  | **1.3 mM Ca^2+^** | **0.3 mM Ca^2+^** |
| -75.2 mV | -59.8 mV |  | -70.0 mV | -68.9 mV |
| -74.7 mV | -61.3 mV |  | -78.7 mV | -64.8 mV |
| -79.6 mV | -63.4 mV |  | -82.6 mV | -63.8 mV |
| -77.1 mV | -60.0 mV |  | -79.9 mV | -66.1 mV |
|  |  |  | -74.2 mV | -62.0 mV |
|  |  |  | -74.7 mV | -54.6 mV |
|  |  |  | -76.0 mV | -59.7 mV |
