# Supplementary material for: A critical period of prehearing spontaneous Ca2+ spiking is required for hair‐bundle maintenance in inner hair cells
Source: EMBO J. 2023 Jan 3;42(4):e112118. doi: 10.15252/embj.2022112118 (PMC9929643; doi:10.15252/embj.2022112118)
Supplement: Supplementary file 14 — Source Data for Figure 8 [file EMBJ-42-e112118-s010.zip › Figure 8/Figure 8A,B,E.docx]

| **Figure 8A** | | | | | |
| --- | --- | --- | --- | --- | --- |
| **Control** | | | **Kir2.1-OE** | | |
| **PC1** | **PC2** |  | | **PC1** | **PC2** |
| 10.6 | -1.66 |  |  | -18.43 | 1.88 |
| 16.8 | 3.906 |  |  | -12.7 | 6.22 |
| 17.52 | -2.46 |  |  | -13.8 | -7.87 |

| **Figure 8AB** | | |
| --- | --- | --- |
| **Control** | **Kir2.1-OE** | |
| 34.16051 |  | 1.68928 |
| 33.34937 |  | 1.64691 |
| 19.58571 |  | 1.70301 |
| 34.16051 |  | 1.68928 |

| **Figure 8E** | | | | | |
| --- | --- | --- | --- | --- | --- |
| ***Myo7a*** | | ***Pcdh15*** | | ***Macf1*** | |
| **Control** | **Kir2.1-OE** | **Control** | **Kir2.1-OE** | **Control** | **Kir2.1-OE** |
| 4.78872 | 14.57944 | 1.70965 | 5.14004 | 3.35527 | 12.73739 |
| 3.89717 | 13.26094 | 1.63958 | 4.09727 | 2.20879 | 9.80312 |
| 4.77149 | 9.67797 | 1.39369 | 3.37267 | 2.3419 | 8.48031 |
| ***Ank2*** | | ***Myh9*** | | ***Map1a*** | |
| **Control** | **Kir2.1-OE** | **Control** | **Kir2.1-OE** | **Control** | **Kir2.1-OE** |
| 17.52502 | 64.07272 | 15.14524 | 52.70201 | 7.86931 | 33.48561 |
| 11.93962 | 45.5057 | 10.97007 | 46.79157 | 3.92533 | 22.15379 |
| 11.80936 | 49.26166 | 11.79589 | 42.08133 | 6.83965 | 47.93466 |
| ***Map1b*** | | ***Cep290*** | | ***Sptbn1*** | |
| **Control** | **Kir2.1-OE** | **Control** | **Kir2.1-OE** | **Control** | **Kir2.1-OE** |
| 21.30291 | 59.46082 | 0.27026 | 1.59558 | 55.2448 | 196.91419 |
| 13.80654 | 54.80921 | 0.14657 | 0.9087 | 42.4587 | 164.60029 |
| 17.29888 | 62.33769 | 0.14726 | 1.01446 | 39.1112 | 147.79467 |
