# Supplementary material for: A critical period of prehearing spontaneous Ca2+ spiking is required for hair‐bundle maintenance in inner hair cells
Source: EMBO J. 2023 Jan 3;42(4):e112118. doi: 10.15252/embj.2022112118 (PMC9929643; doi:10.15252/embj.2022112118)
Supplement: Supplementary file 15 [file EMBJ-42-e112118-s015.pdf]

THE EMBO JOURNAL

A critical period of pre-hearing spontaneous Ca2+ spiking is required for hair-bundle maintenance in inner hair cells

Adam Carlton, Jing-Yi Jeng, Fiorella Grandi, Francesca De Faveri, Federico Ceriani, Lara De Tomasi, Anna Underhill, Stuart Johnson, Kevin Legan, Corne J. Kros, Guy Richardson, Mirna Mustapha, and Walter Marcotti
DOI: 10.15252/embj.2022112118

Corresponding author(s): Walter Marcotti (w.marcotti@sheffield.ac.uk)

Review Timeline:

Submission Date:	16th Jul 22
Editorial Decision:	17th Aug 22
Revision Received:	16th Oct 22
Editorial Decision:	12th Nov 22
Revision Received:	22nd Nov 22
Accepted:	28th Nov 22

Editor: Karin Dumstrei

Transaction Report:

(Note: With the exception of the correction of typographical or spelling errors that could be a source of ambiguity, letters and reports are not edited. Depending on transfer agreements, referee reports obtained elsewhere may or may not be included in this compilation. Referee reports are anonymous unless the Referee chooses to sign their reports.)

Dear Prof. Marcotti,

Thank you for submitting your manuscript to The EMBO Journal. Your study has now been seen by three referees and their comments are provided below.

As you can see from the comments, the referees find the analysis interesting and are supportive of the study for publication here. Should you be able to address the raised concerns then I would like to invite you to submit a revised version.

I think it would be helpful to discuss the revisions further and I am available to do so via email or a video call.

When preparing your letter of response to the referees' comments, please bear in mind that this will form part of the Review Process File, and will therefore be available online to the community. For more details on our Transparent Editorial Process, please visit our website: https://www.embopress.org/page/journal/14602075/authorguide#transparentprocess

Thank you for the opportunity to consider your work for publication. I look forward to discussing your revisions further with you!

Yours sincerely,

Karin

Karin Dumstrei, PhD
Senior Editor
The EMBO Journal

Instructions for preparing your revised manuscript:

I have attached a PDF with helpful tips on how to prepare the revised version.

Guide For Authors:

https://www.embopress.org/page/journal/14602075/authorguide

I realize that it is difficult to revise to a specific deadline. In the interest of protecting the conceptual advance provided by the work, we recommend a revision within 3 months (15th Nov 2022). Should you foresee a problem in meeting this three-month deadline, please let me know in advance as I can grant an extension.

As a matter of policy, competing manuscripts published during this period will not negatively impact on our assessment of the conceptual advance presented by your study.

Use the link below to submit your revision:

https://emboj.msubmit.net/cgi-bin/main.plex

Referee #1:

The manuscript by Carlton et al. investigates the role of calcium action potentials in inner hair cells, that have been known to only occur before hearing onset and are though to inject activity into the auditory system before a sound signal activates the pathway, with the role of refining the developing pathway. This is a highly significant question, and the approach chosen here is appreciated as it provides interesting new insights.

The authors overexpress the inward rectifier Kir2.1 in cochlear inner hair cells, and thereby hyperpolarize the IHC membrane potential by ~ 10 mV, to silence spontaneous firing activity in immature IHC before the inset of hearing. Care is taken in the experimental approaches, for example by using experimental conditions mimicking the endolymph around the IHC stereocilia bundles, and thus predicting that in the second postnatal week no APs in IHCs might be elicited in the Kir2.1 overexpressor.

Results show that membrane changes in IHC due to supporting cell activity still occur (although from a hyperpolarized level). As a result of the Kir2.1 overexpression, a reduced transduction current was found from P8-9 on, possibly due to a reduced calcium sensitivity. At P10-11, the transduction current was found to be very small or absent. Scanning EM shows that bundles appear

normal before P9 and loss and fusion of sterecilia becomes obvious after. These data suggest that calcium APs during second postnatal week are needed for refinement and maintenance of stereocilia bundles and transduction current, an important result for the field. In in vivo experiments, Kir2.1 was removed selectively in postnatal week 1 or 2, and results show that the removal of calcium APs in postnatal week 2 results in effects on bundle structure, pointing to a role in maintenance, again. Additionally, RNA-seq on P9 of WT and K2.1 overexpressor organs of Corti was used to identify candidate genes involved in the process, which will provide starting points for future analysis of mechanisms.

All in all the study is performed carefully, and provides important insights in the role of calcium action potentials during IHC postnatal development. Data are presented clearly and convincingly.

One major issue the reviewer has, is the way the authors separate between supporting cell driving IHC activity and IHC activity, the terms of 'spontaneous' and 'intrinsic' activity which seem to be artificial. This is confusing for the reader, and also not the focus of the otherwise straightforward story (see below)

Major:
Introduction: it is not clear how 'spontaneous' activity in IHCs is defined here. Is it meant to mean 'not sound-induced', or independent from calcium waves in supporting cells?
The reviewer is assuming that calcium APs in IHCs are likely to be induced by supporting cell activity, based on papers from the Bergles lab and others. Do the authors here assume that this is not the case? Do I understand correctly that the authors try to say that IHC APs are initiated separately from input of supporting cells? Is this based on recordings in excised tissue, where the artificially set conditions set the level of the IHC membrane potential? If so, please clarify, and cite the evidence here, as the general reader may not be tuned into these differences in interpretation between research groups.

Results
169-170: intrinsic activity: its not clear here what the author's mean with 'intrinsic', IHC activated and not supporting cell activated?
the reviewer assumes that by hyperpolarizing the IHC, now the supporting cell activity does not reach the AP threshold anymore, so cannot activate the AP as it normally may. Do the authors interpret this differently? Its not clear, due to the word 'intrinsic'.

452 "The mouse model used in this study (Kir2.1-OE) has allowed us to specifically silence the intrinsic Ca2+ action potentials in developing IHCs in vivo, while retaining the ability of non- sensory cells to depolarise the IHCs via the ATP-dependent Ca2+ signalling. "
Although the non-sensory input is kept intact, of course the non- sensory input is not eliciting the signal in IHCs it normally would, as the IHC is hyperpolarized, and thereby the ATP-driven calcium changes in the IHC happen at a more hyperpolarized potential, and are not the same as they would be normally. Therefore I find the distinction here made by the authors not useful, declaring the effects of the ATP driven input the same (which it isn't).

In Summary, the distinction between supporting cell driven activity and 'intrinsic' IHC activity in the IHC seems artificial and besides the point. By hyperpolarizing the HC, whatever extrinsic or intrinsic input is at play, both will have changed impact on the IHC. Studies up until now really can only suggest mechanisms to be involved, no one can prove which input is how large, as conditions in vitro are always different than in vivo. Why then separate and not be inclusive?

For the story here, in the reviewer's opinion, its only important that Ca APs are eliminated and that leads to a loss in function. The separation between intrinsic and extrinsic is not really made experimentally, and therefore (in my opinion) just confuses the reader. I suggest to either provide more evidence in the manuscript for these separate mechanisms, by data or better citations, or to simplify the logic of the current manuscript. This would help the clarity with which the important results are presented.

Minor:
251: 'This membrane hyperpolarization is likely to be compensated...' is a little misleading as a statement, as you show at least in vitro, that its not (line 256). Maybe rather state: "could possibly be compensated.."?

Referee #2:

This study by Carlton and colleagues focuses on the sensory-independent activity of IHCs and SGNs in the mammalian cochlea that occurs during the so called 'critical period' before the onset of hearing. This intrinsically driven activity has been previously shown to be modulated/triggered by ATP waves at supporting cells and spread out to the sensory and neuronal cells of the inner ear. Previous work by this same group has proposed that IHC are able to fire Ca2+ action potentials spontaneously and independently of the support tissue. In the current paper, the authors took an interesting approach by over-expressing a potassium channel (Kir 2.1) only in IHC that reduce their excitability. Interestingly, the 'silent' IHC failed to develop normal hair

bundles and mechano-transduction currents, indicating how important is the sensory independent activity during development. In addition, RNAseq analysis provided information on the gene products that are altered by IHC silencing. Although these sole findings are already interesting and worth the study, some of the conclusions are arguable and poorly backed. The authors also claim that Kir over-expression prevent IHC only from eliciting spontaneous Ca2+ spikes but not those induced by ATP waves. Thus, they attribute the notorious phenotype on stereocilia bundle and mechano-transduction on this differential regulation of the spiking activity. However, some of the evidence put forward for this claim is not well constructed and some experiments do not seem well designed. For instance, whole-cell recordings producing the run-down of Ca2+ currents are not well suited for measuring spiking activity during development. Also, the preference for calcium concentration in calcium imaging experiments could lead to wrongful conclusions. In summary, the study by Carlton and colleagues presents very interesting results on a novel model that limit functional activity during development but incur in doubtful conclusions that could be easily mended by additional experiments and/or editing of some of the important claims.

Major comments:
1) Since much of the results evolves around differences in spiking activity between the first and second postnatal weeks, it would be interesting to show how K+ conductances and membrane potential differ in control vs OE in IHC at the first postnatal week.
2) Figure 2 shows in detail the spiking capacity of IHC at different developmental stages and with high and low Ca2+ concentrations. One important concern with this type of recordings is the run down of the Ca2+ current over a few minutes of whole-cell recording. An alternative would be to use the patch-perforated technique for these experiments. I fear that the wash out of the current may lead to wrongful conclusions.
The authors should provide proof that this issue did not affect their results, using for instance a long stretch of recording showing that spiking activity did not change over time. For both control and OE IHCs.
Despite this issue, a proper quantification of spiking activity should be provided, not only representative traces in each genotype.
3) On this same Figure 2, the authors clearly indicate that they used a low Ca2+ concentration for the imaging experiments on panels F and G, but the reasoning seems odd. Low Ca2+ would mimic the endolymphatic concentration and thus represent the physiological MET current; but on the other hand, ATP waves and Ca2+ signaling in IHC are presumably altered because of the low external Ca2+. The authors should demonstrate experimentally that the conclusion of this figure does not change with a mM concentration of Ca2+, as found in the perilymph.
4) On Figures 3 and 4. Should the authors use DHS, how much would be blocked of the standing current? This experiment is of importance particularly when comparing WT hair cells with the OEs that, as shown in Figure 2, present a significantly larger Kir at rest. Could this current be confounded with the resting MET current?
5) Throughout the study Kir2.1-OE hair cells are compared with those from OtofrtTA+/- mice, which lack Kir2.1 expression. Are these mice of the same litter? Are brothers/sisters compared? Since the presence of the Kir channels in the OE is dependent on the application of DOX to the mice, why not use as control animals those with the same genotype but lacking the use of DOX? Would not this be a better control? Unless there was a good reason for not using this experimental scheme, the authors should show, at least for key experiments, that OtofrtTA+/- mice show similar phenotype than Kir2.1-OE in the absence of DOX.
6) As indicated by comments 1) and 2), from my point of view it is arguable that only spontaneous spikes were responsible for the normal development of hair bundles. But if this was one of the main conclusions of the study, it would be expected that the authors presented a hypothesis for how this spiking activity would differ from that triggered by supporting tissue.

Minor comments:
Page 11, line287. It is presumed that the P= values indicated in this line represent resting open probability of the MET channels. It would be advisable to use an acronym different from P as this was used throughout the manuscript to represent statistical significance. Also, the mean +/- SE should be reported for this measure.

Referee #3:

In this manuscript, Carlton et al. sought to understand the role of spontaneous Ca2+ spikes in IHCs in shaping out their functional development before the onset of hearing (P12). They blocked these transiently emerged spontaneous Ca2+ spikes in IHCs by selectively overexpressing Kir2.1 in them, and they found significant loss and fusion of the stereocilia and almost complete abolishment of the MET current in IHCs with Kir2.1 overexpression. The experimental approach is cleverly designed, the evidence is of high quality and laid out in logic succession, and the manuscript is written in a way that is easy to follow.

My enthusiasm for this study is, however, not without reservation. First of all, the authors relied almost exclusively on Kir2.1 overexpression to manipulate the spontaneous Ca2+ spikes in IHCs. While this does seem to be effective, it comes with caveats, because hyperpolarizing Vm by 10 mV is likely to bear unintended consequences other than blocking spontaneous Ca2+ spikes. Secondly, the significance of this study can be greatly enhanced if the authors include functional analysis of ribbon synapses in IHCs with Kir2.1 overexpression. Given the severity of changes in the MET current and widespread changes in expression of cytoskeletal molecules, functional changes in ribbon synapses are fully expected. Even if no change is found, it adds quite a bit of value to the whole package. By leaving it out now, the authors seem to loss a perfect opportunity to broaden the impact of their study.

Some minor points:

1, Line 23, I would suggest to remove "reversibly" because the authors didn't actually turn on and then back off the Kir2.1 overexpression in the same animals.
2, Line 233 - 238, it is nice to see the inward current and the conductance at -84 mV were increased, confirming the Kir2.1 overexpression, but Vm hyperpolarization can be caused by an outward current only, which was not increased, at least not at strong depolarizing voltages. The authors might want to check the small outward current around the resting Vm to see if it was significantly increased.
3, Line 265 - 267, it is nice to see Ca2+ waves are in sync between IHCs and non-sensory cells, but it looks like it happened less frequently in IHCs. Therefore, the authors might want to examine the frequency and duration of Ca2+ waves in IHCs, given that hyperpolarization of Vm by 10 mV is likely to drive more Ca2+ into the cell.
4, Line 299 - 300, on the reduced Ca2+ sensitivity of the MET current, isn't it due to the Kir2.1 overexpression? The current wording sounds like it is part of normal development change.
5, Line 336 - 353, this is a critical section, and I suggest the authors to provide patch-clamp recordings to show the spontaneous Ca2+ spikes in IHCs come back after removing DOX at P5.
6, Line 822, "superfusion" is confusing here, because it implies the hair bundle was perfused separately with a solution different from the whole preparation. Or maybe this was actually how the experiment conducted?

EMBOJ-2022-112118
A critical period of spontaneous spiking is required for hair-bundle maintenance in inner hair cells

We thank the 3 Reviewers for their positive and constructive comments, which we have addressed in the revised version of the manuscript. Line numbers listed below refer to the Revised pdf manuscript file with “Changes Highlighted”. We have performed several additional experiments and analysis, which have resulted in the following additions:

1) Voltage and current responses from P8-P9 IHCs of control and *Kir2.1-OE* mice when DOX was removed from the drinking water at P5 (Figures 7C-E and Appendix Figure S2).
2) Voltage responses from P4 IHCs of control and *Kir2.1-OE* mice in the continuous presence of DOX (Figure EV1C-I).
3) Capacitance measurements (i.e. exocytosis) from P11 IHCs of control and *Kir2.1-OE* mice in the continuous presence of DOX (Figure EV2E,F).
4) Increased the number of Ca^{2+} wave recording from the non-sensory cells of control and *Kir2.1-OE* mice in the continuous presence of DOX (Figure EV3A,B) and determined the Pearson coefficient between the Ca^{2+} activity in individual IHCs and the Ca^{2+} waves in the non-sensory cells (Figure EV3C).

Reviewer #1
The manuscript by Carlton et al. investigates the role of calcium action potentials in inner hair cells, that have been known to only occur before hearing onset and are though to inject activity into the auditory system before a sound signal activates the pathway, with the role of refining the developing pathway. This is a highly significant question, and the approach chosen here is appreciated as it provides interesting new insights.

The authors overexpress the inward rectifier Kir2.1 in cochlear inner hair cells, and thereby hyperpolarize the IHC membrane potential by ~ 10 mV, to silence spontaneous firing activity in immature IHC before the inset of hearing. Care is taken in the experimental approaches, for example by using experimental conditions mimicking the endolymph around the IHC stereocilia bundles, and thus predicting that in the second postnatal week no APs in IHCs might be elicited in the Kir2.1 overexpressor.

Results show that membrane changes in IHC due to supporting cell activity still occur (although from a hyperpolarized level). As a result of the Kir2.1 overexpression, a reduced transduction current was found from P8-9 on, possibly due to a reduced calcium sensitivity. At P10-11, the transduction current was found to be very small or absent. Scanning EM shows that bundles appear normal before P9 and loss and fusion of sterecilia becomes obvious after. These data suggest that calcium APs during second postnatal week are needed for refinement and maintenance of stereocilia bundles and transduction current, an important result for the field. In in vivo experiments, Kir2.1 was removed selectively in postnatal week 1 or 2, and results show that the removal of calcium APs in postnatal week 2 results in effects on bundle structure, pointing to a role in maintenance, again.

Additionally, RNA-seq on P9 of WT and K2.1 overexpressor organs of Corti was used to identify candidate genes involved in the process, which will provide starting points for future analysis of mechanisms.

All in all the study is performed carefully, and provides important insights in the role of calcium action potentials during IHC postnatal development. Data are presented clearly and convincingly.

One major issue the reviewer has, is the way the authors separate between supporting cell driving IHC activity and IHC activity, the terms of 'spontaneous' and 'intrinsic' activity which seem

to be artificial. This is confusing for the reader, and also not the focus of the otherwise straightforward story (see below)

Thank you for highlighting this issue regarding the use of wording, which we have clarified in our replies to the Major points listed below.

Major:

Introduction: it is not clear how 'spontaneous' activity in IHCs is defined here. Is it meant to mean 'not sound-induced', or independent from calcium waves in supporting cells?

Spontaneous intrinsic action potential activity refers to “not-sound-induced”. We have made this very clear in the revised MS (ln. 40-44).

The reviewer is assuming that calcium APs in IHCs are likely to be induced by supporting cell activity, based on papers from the Bergles lab and others. Do the authors here assume that this is not the case? Do I understand correctly that the authors try to say that IHC APs are initiated separately from input of supporting cells? Is this based on recordings in excised tissue, where the artificially set conditions set the level of the IHC membrane potential? If so, please clarify, and cite the evidence here, as the general reader may not be tuned into these differences in interpretation between research groups.

In recent years there has been a large debate regarding the origin of the Ca^{2+}-induced action potential activity in IHCs. Although the Bergles lab has made several claims regarding the requirements of Ca^{2+} waves from non-sensory cells to elicit AP activity in the IHCs, there is an extensive literature from our and many other labs showing that IHCs are capable of driving intrinsic (spontaneous) AP activity independently from non-sensory cells. To cite a few recent examples, experimental data obtained using near-physiological recordings conditions, in terms of temperature and ionic composition of the extracellular and intracellular solutions, have shown that IHCs exhibit APs in the complete absence of Ca^{2+} waves from non-sensory cells (Johnson et al., 2017 JNeurosci 37:258) and when IHCs are functionally uncoupled from non-sensory cells (Eckrich et al., 2018 11:264). We have also data showing that IHCs exhibit normal voltage responses and spontaneous intrinsic APs in isolation, i.e. mechanically isolated from the rest of the tissue (see Figure below).

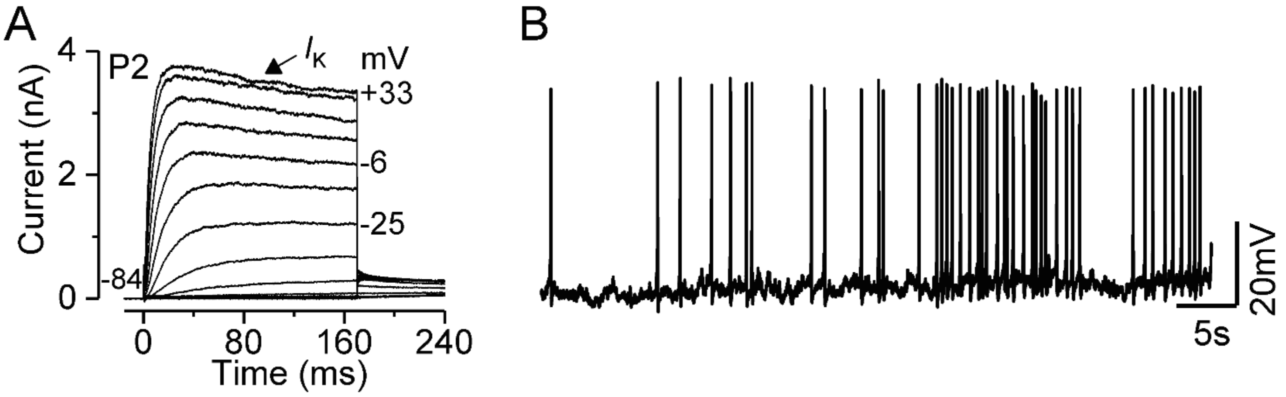

Whole cell recordings from mechanically isolated IHCs

(A) *Outward K^+ current recorded from a mechanically isolated P2 IHCs from a wild-type mouse. Outward currents were elicited using depolarizing voltage steps (10 mV increments) from the holding potential of –84 mV to the various test potentials shown by some of the traces.*

(B) *Example of intrinsic action potentials recorded from the isolated P2 IHC using whole-cell voltage clamp.*

However, the consensus among the different groups is that the intrinsic firing activity of neighbouring IHCs is normally synchronized by spontaneous intercellular Ca^{2+} signalling originating in the non-sensory cells via the release of ATP (Tritsch et al., 2007; Johnson et al., 2011; Wang et al., 2015; Eckrich et al., 2018; Johnson et al., 2017).

The presence of these two spontaneous Ca^{2+}-dependent mechanisms (intrinsic Ca^{2+} APs in IHCs and their extrinsic modulation by Ca^{2+}-waves originating in the non-sensory cells) was the reason driving the design of the Kir2.1 overexpressing mouse model to identify the role of the intrinsic activity in the IHCs. We have better highlighted the presence of two distinct mechanisms in the revised Introduction (ln. 40-68).

Results

169-170: intrinsic activity: its not clear here what the author's mean with 'intrinsic', IHC activated and not supporting cell activated? the reviewer assumes that by hyperpolarizing the IHC, now the supporting cell activity does not reach the AP threshold anymore, so cannot activate the AP as it normally may. Do the authors interpret this differently? Its not clear, due to the word 'intrinsic'.

As mentioned above, the IHCs elicit APs independently from the non-sensory cells, which we refer to as "intrinsic". We have now clarified this in the Introduction (ln. 40-44) and at the beginning of the Result section (ln. 78-81) of the revised MS. Our data show that IHC hyperpolarization induced by the overexpression of Kir2.1 prevented the "intrinsic" AP activity, but did not affect the ability of Ca^{2+} waves originating in the non-sensory cells, to elicit Ca^{2+} transients in IHCs (Figure 2).

452 "The mouse model used in this study (Kir2.1-OE) has allowed us to specifically silence the intrinsic Ca2+ action potentials in developing IHCs in vivo, while retaining the ability of non-sensory cells to depolarise the IHCs via the ATP-dependent Ca2+ signalling." Although the non-sensory input is kept intact, of course the non- sensory input is not eliciting the signal in IHCs it normally would, as the IHC is hyperpolarized, and thereby the ATP-driven calcium changes in the IHC happen at a more hyperpolarized potential, and are not the same as they would be normally. Therefore I find the distinction here made by the authors not useful, declaring the effects of the ATP driven input the same (which it isn't).

We fully appreciate the reviewer's concern, which is the reason why the above statement (now ln. 329-331) uses a cautious wording "…, <u>while retaining the ability</u> of non- sensory cells to depolarise the IHCs….", which does not assume "identical" responses. However, intrinsic action potentials in the IHCs are driven by depolarization-induced opening of Ca^{2+} channels, which activate at around -65 mV. Similarly, non-sensory cells elicit/synchronise Ca^{2+} action potentials in IHCs by increasing the K^{+} concentration in the extracellular space (ATP-mediated mechanism: see ln: 315-321), which again leads to Ca^{2+} channel activation at around -65 mV. This means that the activation of the Ca^{2+} channels is completely independent from the IHC resting membrane potential. Therefore, despite the more hyperpolarised membrane potential of the IHCs from Kir2.1-OE, the externally driven ATP-induced Ca^{2+} spikes in IHCs will be elicited as soon as the threshold for the activation of Ca^{2+} channels at about -65 mV is reached (not at "more hyperpolarised potentials"). Considering that the ATP-induced activity from non-sensory cells generally leads to up to 28 mV IHC depolarization (Tritsch et al., 2007), this extracellular signal is more than sufficient to reach the threshold for action potentials in IHCs. We have included some of the above reasoning in the revised results (ln: 89-95 and ln: 123-138).

In Summary, the distinction between supporting cell driven activity and 'intrinsic' IHC activity in the IHC seems artificial and besides the point. By hyperpolarizing the HC, whatever extrinsic or intrinsic input is at play, both will have changed impact on the IHC. Studies up until now really can only suggest mechanisms to be involved, no one can prove which input is how large, as conditions in vitro are always different than in vivo. Why then separate and not be inclusive?

We hope that the above explanation convinces this reviewer of the pre-existing evidence for two different types of activity, as supported by extensive literature on the subject. The separation between the two mechanisms was the driver for the generation of the Kir2.1 mouse model, which

leaves the activity of the non-sensory cells intact and also, as mentioned above, their ability to modulate the IHC firing activity.

For the story here, in the reviewer's opinion, its only important that Ca APs are eliminated and that leads to a loss in function. The separation between intrinsic and extrinsic is not really made experimentally, and therefore (in my opinion) just confuses the reader. I suggest to either provide more evidence in the manuscript for these separate mechanisms, by data or better citations, or to simplify the logic of the current manuscript. This would help the clarity with which the important results are presented.

As mentioned above, there is substantial experimental evidence in the literature indicating the presence of two separate mechanisms, which work together to regulate different aspects of the auditory system development.

Minor:

251: 'This membrane hyperpolarization is likely to be compensated...' is a little misleading as a statement, as you show at least in vitro, that its not (line 256). Maybe rather state: "could possibly be compensated.."?

This statement was based on a study we published in 2012 (Johnson et al., 2012 JNeurosci 32, 10479). In IHCs, the resting membrane potential is determined by the combined effect of the outward hyperpolarizing basolateral membrane K^+ currents and the inward depolarising MET current. When using *ex vivo* preparations, experiments are normally done using 1.3 mM Ca^{2+}, which is the concentration of the extracellular solution. However, Ca^{2+} is a permeant blocker of the MET channels, thus largely reducing its contribution on the setting of the resting membrane potential. The result is that the post-natal increase of the K^+ current progressively hyperpolarises the IHCs because of the smaller depolarising MET current under *ex vivo* conditions. In 2012, we published a study showing that when using the *in vivo* estimated Ca^{2+} concentration in the endolymph from the pre-hearing cochlea (0.3 mM), the increased resting open probability of the MET channel was sufficient to compensate for the progressive hyperpolarization of the IHC membrane potentials. We now include the reference in the statement to indicate that this has been proven experimentally.

Reviewer #2

This study by Carlton and colleagues focuses on the sensory-independent activity of IHCs and SGNs in the mammalian cochlea that occurs during the so called 'critical period' before the onset of hearing. This intrinsically driven activity has been previously shown to be modulated/triggered by ATP waves at supporting cells and spread out to the sensory and neuronal cells of the inner ear. Previous work by this same group has proposed that IHC are able to fire Ca2+ action potentials spontaneously and independently of the support tissue. In the current paper, the authors took an interesting approach by over-expressing a potassium channel (Kir 2.1) only in IHC that reduce their excitability. Interestingly, the 'silent' IHC failed to develop normal hair bundles and mechano-transduction currents, indicating how important is the sensory independent activity during development. In addition, RNAseq analysis provided information on the gene products that are altered by IHC silencing. Although these sole findings are already interesting and worth the study, some of the conclusions are arguable and poorly backed. The authors also claim that Kir over-expression prevent IHC only from eliciting spontaneous Ca2+ spikes but not those induced by ATP waves. Thus, they attribute the notorious phenotype on stereocilia bundle and mechano-transduction on this differential regulation of the spiking activity. However, some of the evidence put forward for this claim is not well constructed and some experiments do not seem well designed. For instance, whole-cell recordings producing the run-down of Ca2+ currents are not well suited for measuring spiking activity during development. Also, the preference for calcium concentration in calcium imaging experiments could lead to wrongful conclusions. In summary, the study by Carlton and colleagues presents very interesting results on a novel model that limit functional activity

during development but incur in doubtful conclusions that could be easily mended by additional experiments and/or editing of some of the important claims.

Although most of the above concerns are highlighted by the Major points below, here we would like to clarify the point related to the run-down of the Ca^{2+} current under whole cell recordings. There are several aspects that need to be considered:

1) The aim of this study is not to study the biophysical characteristics of the action potential activity in the IHCs, but instead to understand their role *in vivo*. The *ex vivo* recordings (Figure 2) were only used to test the efficacy of the *Kir2.1 OE* mouse model in preventing spontaneous firing in the IHCs. Whole-cell experiments are extremely reliable when performing short-time window recordings (see below).

2) The biophysical characteristics of the Ca^{2+}-dependent action potentials in developing IHCs have already been investigated in great details by several groups using whole cell (e.g. Johnson et al., [Marcotti] 2011 Nature Neurosci 14:711; Johnson et al., [Marcotti] 2012 JNeurosci 32: 10479), perforated patch (e.g. Harrus et al., 2018 Front Cell Neurosci, 12:407) and cell-attached (Johnson et al., 2011) recordings. Our group has used both whole-cell and cell-attached recordings. Our previous data quoted above that the whole-cell approach is extremely reliable when recording Ca^{2+} action potentials from IHCs using short-time windows, like those used in the present work.

3) Finally, the Ca^{2+} current in pre-hearing IHCs shows very little run-down during repetitive stimulations, at least under our whole cell recording conditions (e.g. Johnson and Marcotti, 2009 JPhysiol 586:1029).

4) The low Ca^{2+} concentration used for electrophysiology and imaging experiments [0.3 mM Ca^{2+}] was used to mimic as close as possible the endolymph present in the *in vivo* cochlea (Johnson et al., 2012). The endolymph is bathing not only the IHC hair bundle but also the apical portion of the non-sensory cells, which by releasing ATP via connexin hemichannels regulate the IHC firing activity.

Major comments:

1) Since much of the results evolves around differences in spiking activity between the first and second postnatal weeks, it would be interesting to show how K+ conductances and membrane potential differ in control vs OE in IHC at the first postnatal week.

The voltage responses in P4 IHCs (first postnatal week) were already included in the submitted Figure 2A,B. We have now performed additional experiment to include the current responses, which are now included in the new Figure AV1 and briefly described in the revised MS (ln. 82-95).

2) Figure 2 shows in detail the spiking capacity of IHC at different developmental stages and with high and low Ca2+ concentrations. One important concern with this type of recordings is the run down of the Ca2+ current over a few minutes of whole-cell recording. An alternative would be to use the patch-perforated technique for these experiments. I fear that the wash out of the current may lead to wrongful conclusions. The authors should provide proof that this issue did not affect their results, using for instance a long stretch of recording showing that spiking activity did not change over time. For both control and OE IHCs. Despite this issue, a proper quantification of spiking activity should be provided, not only representative traces in each genotype.

As explained above, the aim of this study is to investigate the role of the spontaneous action potentials and not their biophysical characteristics, which are already well know. Also, IHCs from *Kir2.1 OE* mice do not show any spontaneous intrinsic action potentials, so we cannot quantify any of the requested properties. Figure 2A,B was included to prove that the *Kir2.1 OE* mouse model was effective in removing the spontaneous action potential activity in the IHCs, which it does.

3) On this same Figure 2, the authors clearly indicate that they used a low Ca2+ concentration for the imaging experiments on panels F and G, but the reasoning seems odd. Low Ca2+ would mimic

the endolymphatic concentration and thus represent the physiological MET current; but on the other hand, ATP waves and Ca2+ signaling in IHC are presumably altered because of the low external Ca2 +. The authors should demonstrate experimentally that the conclusion of this figure does not change with a mM concentration of Ca2+, as found in the perilymph.

In vivo, the endolymph is bathing not only the hair bundles of the IHCs but also the apical membrane of the non-sensory cells. During the second postnatal week, the low extracellular calcium concentration (300 µM, compared to the perilymphatic concentration of 1.3 mM) was chosen to approximate the i*n vivo* condition (Johnson et al., 2012). Since we use the same experimental conditions in both control and *Kir2.1 OE* mice, any difference in the IHC Ca^{2+} signals is due to the Kir2.1 expression.

Additional key information:

1) The low endolymphatic Ca^{2+} concentration is also known to increase both the propagation range of intercellular Ca^{2+} waves in the lesser epithelial ridge and the frequency of spontaneous Ca^{2+} transient in the greater epithelial ridge (Anselmi et al., 2008 PNAS 105:18770, Ceriani and Mammano 2013, Cell Comm Sig 10:20). This is because the low endolymphatic Ca^{2+} concentration is thought to increase the open probability of the connexin hemichannels present in the apical membrane of the non-sensory cells (Saez et al., 2005 Biochim Biophys Acta 1711:215), which promotes the release of ATP (Anselmi et al., 2008 PNAS 105:18770). As connexin hemichannels face the endolymphatic surface of the epithelium (Ceriani and Mammano 2013), and therefore experience micromolar levels of Ca^{2+} *in vivo* (about 300 µM), our experimental setting will produce spontaneous Ca^{2+} wave dynamics that are closer to those present *in vivo* during the second postnatal week. See also see Figure 3 from Mammano F (2013). ATP-dependent intercellular Ca2+ signaling in the developing cochlea: facts, fantasies and perspectives. Semin Cell Dev Biol 24:31-39. Mammano.

2) ATP-induced Ca^{2+} transients and spontaneous Ca^{2+} waves in cochlear non-sensory cells are mainly dependent on metabotropic purinergic receptors (Anselmi et al., 2008, Piazza et al., 2007 Cell Calcium 41:77; Babola et al., 2021 JNeurosci 41:594), and thus on Ca^{2+} release from intracellular stores. Therefore, lowering extracellular Ca^{2+} would not affect Ca^{2+} release in non-sensory cells, but only promote the increased open probability of the connexin hemichannels in the non-sensory cells as expected *in vivo*.

4) On Figures 3 and 4. Should the authors use DHS, how much would be blocked of the standing current? This experiment is of importance particularly when comparing WT hair cells with the OEs that, as shown in Figure 2, present a significantly larger Kir at rest. Could this current be confounded with the resting MET current?

The standing MET current is only generated by the resting open probability of the MET channels, which has been extensively demonstrated by several studies (e.g. Corns et al., [Marcotti] 2014 PNAS 111:14918). The presence or absence of Kir2.1 in the basolateral membrane of the

IHCs does not influence the open probability of the MET channel. As shown in the Figure below, the MET current is elicited on top of the steady-state “holding” current, which is determined by the outward or inward K^+ currents. The resting open probability of the MET current is calculated by dividing the resting MET current (the difference between the current level before the stimulus, indicated by the dashed line, and the current level at the negative phase of the stimulus when all channels were closed) by the maximum peak-to-peak MET current. We have now included this description in the revised legend of Figure 3 (ln. 912-916).

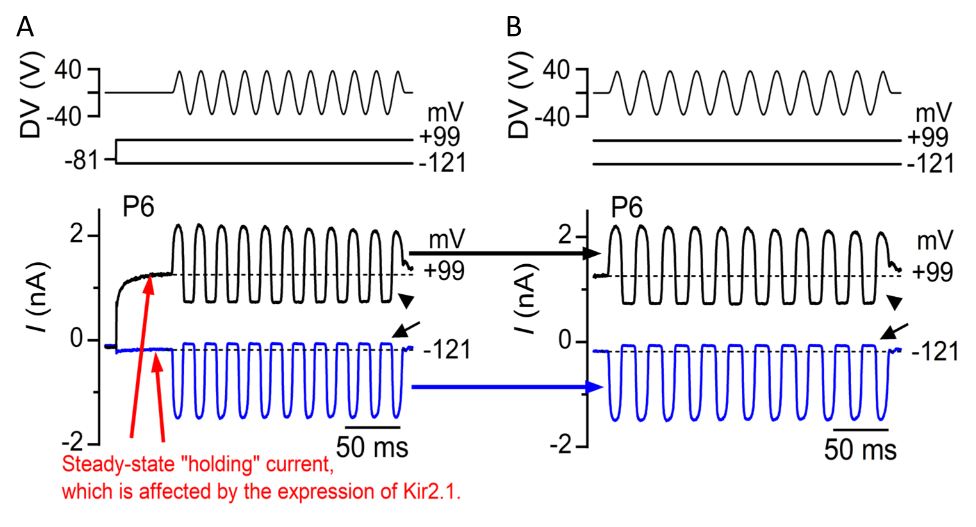

A, MET current recordings at two membrane potentials (-121 mV and +99 mV) elicited using a sinewave stimulus (top). The steady-state “holding” current is generated by the inward and outward K^+ currents. **B,** MET current shown in isolation from the same recording in panel **A**.

Nevertheless, most of the MET current recordings were performed using 5 mM CsCl in the extracellular solution, which fully block the inward rectifier K^+ current (Marcotti et al., 1999 Pflugers Arch 439:113). We now include this information in the revised Method section (ln. 450-453; ln. 459-462).

5) Throughout the study Kir2.1-OE hair cells are compared with those from OtofrtTA+/- mice, which lack Kir2.1 expression. Are these mice of the same litter? Are brothers/sisters compared? Since the presence of the Kir channels in the OE is dependent on the application of DOX to the mice, why not use as control animals those with the same genotype but lacking the use of DOX? Would not this be a better control? Unless there was a good reason for not using this experimental scheme, the authors should show, at least for key experiments, that OtofrtTA+/- mice show similar phenotype than Kir2.1-OE in the absence of DOX.

Correct, we always use littermate controls for our experiments. The correct control animals are always those that are subjected to the same external treatments and only different for the specific mutation under investigation. Using separate WT mice as controls would add unknown variabilities related to possible side effects produced by the application of DOX. In addition to test the rtTA-Otof and the Kir2.1-rtTA-Otof (Kir2.1-OE) mice, we also have performed experiments in the Kir2.1 mice (submitted Supplementary Figure 6), to make sure DOX was not causing any unwanted effect on this mouse line. Even in the presence of DOX, the IHCs from both the rtTA-Otof and the Kir2.1 develop and function as previously shown in wild-type mice, which means that DOX does not affect them. This is also consistent with our previous work on IHCs using DOX in mice during development (Johnson et al., 2013 PNAS 110:8720).

6) As indicated by comments 1) and 2), from my point of view it is arguable that only spontaneous spikes were responsible for the normal development of hair bundles. But if this was one of the main conclusions of the study, it would be expected that the authors presented a hypothesis for how this spiking activity would differ from that triggered by supporting tissue.

There is an extensive literature from our and many other labs showing that IHCs are capable of driving spontaneous activity independently from non-sensory cells (see also the Figure used in the

reply to Reviewer 1). To cite a few recent examples, experimental data obtained using near-physiological recordings conditions, in terms of temperature and ionic composition of the extracellular and intracellular solutions, have shown that IHCs exhibit APs in the complete absence of Ca^{2+} waves from non-sensory cells (Johnson et al., [Marcotti] 2017 JNeurosci 37:258), when IHCs are functionally uncoupled from non-sensory cells (Eckrich et al., [Engel] 2018 11:264) or even when IHCs are mechanically isolated from the rest of the tissue (Ceriani et al. [Mammano] https://doi.org/10.1101/731851). Calcium waves from non-sensory cells modulate the frequency and pattern of the "intrinsic" firing activity present in the developing IHCs.

Minor comments:
Page 11, line287. It is presumed that the P= values indicated in this line represent resting open probability of the MET channels. It would be advisable to use an acronym different from P as this was used throughout the manuscript to represent statistical significance. Also, the mean +/- SE should be reported for this measure.

The "P" value indicated in ln 287 of the submitted MS refers to statistical significance, so it is correct. For MET channel open probability, we used "P_{open}".

Reviewer #3
In this manuscript, Carlton et al. sought to understand the role of spontaneous Ca2+ spikes in IHCs in shaping out their functional development before the onset of hearing (P12). They blocked these transiently emerged spontaneous Ca2+ spikes in IHCs by selectively overexpressing Kir2.1 in them, and they found significant loss and fusion of the stereocilia and almost complete abolishment of the MET current in IHCs with Kir2.1 overexpression. The experimental approach is cleverly designed, the evidence is of high quality and laid out in logic succession, and the manuscript is written in a way that is easy to follow.
Thank you.

My enthusiasm for this study is, however, not without reservation. First of all, the authors relied almost exclusively on Kir2.1 overexpression to manipulate the spontaneous Ca2+ spikes in IHCs. While this does seem to be effective, it comes with caveats, because hyperpolarizing Vm by 10 mV is likely to bear unintended consequences other than blocking spontaneous Ca2+ spikes.

First of all, we would like to emphasize that a similar mouse model, which leads to the DOX-inducible expression of Kir2.1 channels and membrane hyperpolarization, has previously been used in the olfactory system (Yu et al., 2004 Neuron 42:553).

Although action potentials in IHCs can be modulated or prevented by interfering with one of the several channels contributing to their waveform (e.g. $Ca_V1.3$ Ca^{2+} channels; inward and outward rectifying K^+ channels; Na^+ channels), none of them will allow the IHCs to fire normal action potentials following Ca^{2+} waves in the non-sensory cells. This experimental model has allowed us to investigate specifically the role of the "intrinsic" firing activity in the IHCs.

Despite the above explanations, we fully understand the point made by this reviewer, which is the reason why we performed several control experiments:

1) IHCs develop normally in the individual mouse strains treated with DOX (Kir2.1 and rtTA-Otof).

2) In the Kir2.1-OE mice, the application of DOX did not affect the OHCs, which is consistent with the use of the specific promoter Otoferlin.

3) Regarding the IHCs in the Kir2.1-OE mice, we have investigated all the key biophysical and morphological properties during cochlear development, with the altered MET apparatus being the only phenotype observed.

Secondly, the significance of this study can be greatly enhanced if the authors include functional analysis of ribbon synapses in IHCs with Kir2.1 overexpression. Given the severity of changes in the MET current and widespread changes in expression of cytoskeletal molecules, functional changes in ribbon synapses are fully expected. Even if no change is found, it adds quite a bit of value to the whole package. By leaving it out now, the authors seem to loss a perfect opportunity to broaden the impact of their study.

As requested, we have performed a functional investigation of the ribbon synapses. Our findings are now included in Figure EV2 and described in the Result section (ln. 95-101). Both the morphology of ribbon synapse and exocytosis were comparable between the two genotypes.

Some minor points:

1, Line 23, I would suggest to remove "reversibly" because the authors didn't actually turn on and then back off the Kir2.1 overexpression in the same animals.

We are a bit puzzled by this statement because we did turn on and then off the Kir2.1 expression in some of the mice investigated: see the experiments describing the removal of DOX in Figure 7 and Supplementary Figure 7. Although this was not done for all animals, we proved that the effects associated with the overexpression of the Kir2.1 channels are "reversible". Therefore, we would like to retain this word in the Abstract.

2, Line 233 - 238, it is nice to see the inward current and the conductance at -84 mV were increased, confirming the Kir2.1 overexpression, but Vm hyperpolarization can be caused by an outward current only, which was not increased, at least not at strong depolarizing voltages. The authors might want to check the small outward current around the resting Vm to see if it was significantly increased.

The membrane potential of the immature IHCs is set by the inward rectifier K^+ current. A characteristic property of this group of inward rectifying K^+ current is that they are outward at around the resting membrane potentials (e.g. Hille 2002, pp 150-154; Marcotti et al., 1999). We have now recalculated the conductance around the respective membrane potentials, which is still highly significantly increased (ln. 91-93 and ln. 863-864).

3, Line 265 - 267, it is nice to see Ca2+ waves are in sync between IHCs and non-sensory cells, but it looks like it happened less frequently in IHCs. Therefore, the authors might want to examine the frequency and duration of Ca2+ waves in IHCs, given that hyperpolarization of Vm by 10 mV is likely to drive more Ca2+ into the cell.

We have performed additional experiments and analysis to investigate the level of correlation between the Ca^{2+} waves in non-sensory cells and Ca^{2+} signals in the IHCs. As shown in the revised Fig EV3, the above correlation was not found to be significantly different between the two genotypes (ln. 134-135; see also the legend of Fig EV3). Regarding the second part of the question, repetitive action potential activity is triggered when the membrane potential of IHCs reaches the threshold value (around -60 mV), which is independent from the starting Vm. If Ca^{2+} waves from non-sensory cells are able to depolarise the IHCs to the threshold value, then the IHCs from Kir2.1-OE mice will fire action potentials even if they are 10 mV more hyperpolarised at rest than control cells.

4, Line 299 - 300, on the reduced Ca2+ sensitivity of the MET current, isn't it due to the Kir2.1 overexpression? The current sounds like it is part of normal development change.

We have revised the text to address this issue (ln. 168-170).

5, Line 336 - 353, this is a critical section, and I suggest the authors to provide patch-clamp recordings to show the spontaneous Ca2+ spikes in IHCs come back after removing DOX at P5.

As suggested, we have performed new electrophysiological recordings from the IHCs of mice treated with DOX, which was then removed from the drinking water when the pups were P5. As expected from the normal development of the hair bundles, the IHCs from Kir2.1-OE mice exhibit voltage responses comparable to cells from control mice. These new data are shown in Fig 7C-F and Appendix S2 and described in the Results (ln. 213-224).

6, Line 822, "superfusion" is confusing here, because it implies the hair bundle was perfused separately with a solution different from the whole preparation. Or maybe this was actually how the experiment conducted?

We have changed “superfusion” with “application” (ln. 875-877).

1st Revision - Editorial Decision 12th Nov 2022

Dear Walter,

Thank you for submitting your revised manuscript to The EMBO Journal. Your study has now been seen by the original referees and their comments are provided below.

The referees appreciate the added data. However, they also have some remaining issues that I would like to ask you to address in a final revision. Referee #2 would like to see some quantification of the data. I anticipate that this should be fairly straight forward to respond to. Let me know if that is not the case.

Both referees #1 and 2 also have some remaining issues with the interpretation of the data. I am OK with that you have a different interpretation but do take their concerns into consideration. Please also make sure that you have a balanced discussion on this point.

Let me know if we need to discuss any of the raised issues further. Happy to do so via email or video if needed.

When you submit the revised manuscript will you also take of the following editorial points:

- You can only have 5 keywords

- Add the accession number for the deposited RNA seq data

- COI needs to be re-labelled as Disclosure and competing interests statement.

- Please remove the Authors Contributions from the manuscript. The 'Author Contributions' section is replaced by the CRediT contributor roles taxonomy to specify the contributions of each author in the journal submission system. Please use the free text box in the 'author information' section of the manuscript submisssion system to provide more detailed descriptions (e.g., 'X provided intracellular Ca++ measurements in fig Y')

- Check figure callouts for Fig 7F and 7G. Supplementary Figure 6 is called out, but no such figure.

- The appendix need a ToC

- Please check the size of the synopsis image - should be 550 wide by [200-400] high

- I have turned Table AV1-AV2 into datasets and labelled the files Dataset EV1-EV2. Could you please correct callout in text to these files and add the legend to a separate sheet of the Excel file. Table legends should be removed from MS file.

- Our publisher has also done their pre-publication check on your manuscript. When you log into the manuscript submission system you will see the file "Data Edited Manuscript file". Please take a look at the word file and the comments regarding the figure legends and respond to the issues.

- Please include a point-by-point response also to the editorial comments.

- when you resubmit the revised manuscript please submit the MS text file without any marked changes.

Let me know if you have any further questions

With best wishes

Karin

Karin Dumstrei, PhD
Senior Editor
The EMBO Journal

Instructions for preparing your revised manuscript:

Guide For Authors: https://www.embopress.org/page/journal/14602075/authorguide

Use the link below to submit your revision:

Referee #1:

The manuscript by Carlton et al. investigates the role of calcium action potentials in inner hair cells, that have been known to only occur before hearing onset and are thought to inject activity into the auditory system before a sound signal activates the pathway, with the role of refining the developing pathway. This is a highly significant question, and the approach chosen here is appreciated as it provides interesting new insights, showing that the loss of this activity results in defects in mechano-transduction.

The revised manuscript by Carlton et al. adds new data for better controls, and provides good explanations in response to some technical issues reviewers had pointed out. Also, better citing of the literature puts the study into a better context. I consider the experimental approaches and results as solid.
This reviewer had questioned the distinction between 'intrinsic' IHC activation and supporting cell induced AP activation in IHCs. Even if IHC APs can occur in settings without supporting cell activity (as now cited), it still doesn't tell us what happens exactly in vivo. The reviewer finds the distinction artificial and feels that the study here doesn't really speak to these different sources of activation, as manipulations may affect both, the IHCs and the supporting cells ability to induce APs.

The authors have not taken any of the comments of the reviewer's (1 and 2) on this topic to heart, and continue their approach of interpreting intrinsic and supporting cell activation as separate mechanisms, to be investigated here.

At this point, the reviewer's opinion is that one can agree to disagree; input has been given by the reviewer and was not taken by the authors, leading to different interpretations of the data. However, the study shows solid effects, and provides significant insights into the role of calcium action potentials in the developing cochlea.

The reviewer therefore has no further concerns.

Referee #2:

In this new version of their manuscript, Carlton and colleagues made an effort to respond my critics with additional experiments and providing explanations for some methods and results.
- With respect to my critics on Figure 2 of the initial version of the ms, some disagreement persists with the authors' justification. Figures 2A and 2B include only a very short stretch of recording showing differences in spiking activity between WT and OE cells. Given the bursting characteristic of IHCs firing with long stretches of silence and periods with strong activity, a good quantification requires more than a few ms of recording. But there is no quantification whatsoever of the firing rate in these two genotypes. One can agree that the phenotype is very likely a descent in spiking rate, but a proper quantification is necessary. Even if the rate is equal to zero as indicated for the OE in the first postnatal week.
The authors made some important claims on how Kir2.1-OE affected development due to the cancellation of 'spontaneous' action potentials but not ATP-evoked ('Without intrinsic Ca2+ APs, mechanoelectrical transduction in IHCSs disappears prior to hearing onset due to progressive loss or fusion of stereocilia'). These claims should be backed with a quantification of differences / similarities in firing at these two stages between WT and OE. By the way, do OE still fire in bursts? How much reduced is the firing rate? This quantification is still lacking, and it would be interesting to know if the bursting behavior changes or remains intact.
In my opinion, the authors made a strong statement in relation to differences in the first vs second postnatal weeks that is not indispensable for supporting the main conclusion of the paper. The finding of a strong phenotype in stereocilia and MET of OE mice does not require to be paralleled with a phenotype in spiking only in the second week (although a proper quantification would always be required). I feel that the authors self-inflected the problem of demonstrating these differences.
- On the Ca2+ concentration chosen for imaging experiments. The authors made a compelling justification for why endolymphatic concentration is more physiological than the perilymphatic from the standpoint of the generation and propagation from supporting cells. But action potential generation will clearly differ in IHCs at low external Ca2+. And this is a greatly important point of the study. It is true that in the end a conclusion would be made on the differences between WT and OE, whichever Ca2+ concentration is used. But this brings me back to point 1) and the importance of showing true difference in spiking activity between these two genotypes.
- The authors added interesting data in their new figure 7, which points at answering the question of whether these developmental alterations that are due to the lack of IHCs spiking activity can revert by interrupting the over-expression of the Kir2.1. Data seems nice, but similarly to what was indicated before, a real quantification of spike rates averages +/- error and statistics (not only Vm) in different genotypes and treatments is lacking.

Referee #3:

The authors have resolved all the issues I raised, in a responsive and satisfactory manner, and I have no further comments.

EMBOJ-2022-112118R
A critical period of spontaneous spiking is required for hair-bundle maintenance in inner hair cells

Editorial points:

- You can only have 5 keywords

Done

- Add the accession number for the deposited RNA seq data

Done

- COI needs to be re-labelled as Disclosure and competing interests statement.

Done

- Please remove the Authors Contributions from the manuscript. The 'Author Contributions' section is replaced by the CRediT contributor roles taxonomy to specify the contributions of each author in the journal submission system. Please use the free text box in the 'author information' section of the manuscript submisssion system to provide more detailed descriptions (e.g., 'X provided intracellular Ca++ measurements in fig Y')

Done

- Check figure callouts for Fig 7F and 7G. Supplementary Figure 6 is called out, but no such figure.

Both issues have been addressed in the revised MS.

- The appendix need a ToC

We have included the list of Figures at the beginning of the Appendix document.

- Please check the size of the synopsis image - should be 550 wide by [200-400] high

The Synopsis image is now 500 X 400 dpi

- I have turned Table AV1-AV2 into datasets and labelled the files Dataset EV1-EV2. Could you please correct callout in text to these files and add the legend to a separate sheet of the Excel file. Table legends should be removed from MS file.

We have changes Table AV1-AV2 to Dataset AV1-AV2 in the main document.
We have also moved the legend into the Exel files.

- Our publisher has also done their pre-publication check on your manuscript. When you log into the manuscript submission system you will see the file "Data Edited Manuscript file". Please take a look at the word file and the comments regarding the figure legends and respond to the issues.

Main Figure legends

We are a bit confused about some of the comments about the "error bars" and "biological/technical replica" since they are stated at the end of each Figure legend under "Data information".
Figure 4 legend: Statistical text and the meaning of "*" are now specified under "Data information".
Figure 5 legend: the meaning of the asterisks is not defined.
Figure 7 legend: correct, thank you.

Expanded View Figure legends

Figure EV1 legend: the "error bars" is specified in the "Data information" at the end of all panels. Thank you for moving the statistical statement below.
Figure EV2 legend: the "error bars" in panels E,F is now specified.
Figure EV4 legend: Correct, thank you.

Reviewer #1

The manuscript by Carlton et al. investigates the role of calcium action potentials in inner hair cells, that have been known to only occur before hearing onset and are thought to inject activity into the auditory system before a sound signal activates the pathway, with the role of refining the developing pathway. This is a highly significant question, and the approach chosen here is

appreciated as it provides interesting new insights, showing that the loss of this activity results in defects in mechano-transduction.

The revised manuscript by Carlton et al. adds new data for better controls, and provides good explanations in response to some technical issues reviewers had pointed out. Also, better citing of the literature puts the study into a better context. I consider the experimental approaches and results as solid.

This reviewer had questioned the distinction between 'intrinsic' IHC activation and supporting cell induced AP activation in IHCs. Even if IHC APs can occur in settings without supporting cell activity (as now cited), it still doesn't tell us what happens exactly in vivo.

In vivo recording of AP activity from the mammalian cochlea is currently considered to be technically unfeasible. However, we are one the very few laboratories in the world performing experiments *ex vivo* under near-physiological conditions that are approximating as closer as possible those *in vivo*.

The reviewer finds the distinction artificial and feels that the study here doesn't really speak to these different sources of activation, as manipulations may affect both, the IHCs and the supporting cells ability to induce APs. The authors have not taken any of the comments of the reviewer's (1 and 2) on this topic to heart, and continue their approach of interpreting intrinsic and supporting cell activation as separate mechanisms, to be investigated here.

In the revised version of the manuscript, we have re-written several sections to explain the scientific reasons for the presence of two Ca^{2+}-dependent mechanisms regulating the developing cochlea. These reasons are based on several years of published work from several laboratories, including ours.

The presence of these two mechanisms was the key driver for the development of this study. The experimental design was aimed at affecting only one (intrinsic to the IHCs) and not the other (modulation of IHC APs by the non-sensory cells).

We have provided several lines evidence in the submitted version, and more in the revised version, that the overexpression of Kir2.1, which is controlled by the IHC specific promoter otoferlin, targets the activity in the IHCs, but not that of the supporting cells. This is based on experimental evidence and quantification.

In order to provide a fair representation of the published literature, which is key to justify the rationale behind our study, we have no choice but to introduce and discuss both mechanisms.

Regarding the comments related to Reviewer 2, we have addressed all his/her previous comments apart the quantifications of the AP activity, which we have now included.

At this point, the reviewer's opinion is that one can agree to disagree; input has been given by the reviewer and was not taken by the authors, leading to different interpretations of the data. However, the study shows solid effects, and provides significant insights into the role of calcium action potentials in the developing cochlea.

Thank you for the appreciation of the study.

Reviewer #2

In this new version of their manuscript, Carlton and colleagues made an effort to respond my critics with additional experiments and providing explanations for some methods and results.

Thank you.

- With respect to my critics on Figure 2 of the initial version of the ms, some disagreement persists with the authors' justification. Figures 2A and 2B include only a very short stretch of recording showing differences in spiking activity between WT and OE cells. Given the bursting characteristic of IHCs firing with long stretches of silence and periods with strong activity, a good quantification

requires more than a few ms of recording. But there is no quantification whatsoever of the firing rate in these two genotypes. One can agree that the phenotype is very likely a descent in spiking rate, but a proper quantification is necessary. Even if the rate is equal to zero as indicated for the OE in the first postnatal week.
The authors made some important claims on how Kir2.1-OE affected development due to the cancellation of 'spontaneous' action potentials but not ATP-evoked ('Without intrinsic Ca2+ APs, mechanoelectrical transduction in IHCSs disappears prior to hearing onset due to progressive loss or fusion of stereocilia'). These claims should be backed with a quantification of differences / similarities in firing at these two stages between WT and OE. By the way, do OE still fire in bursts? How much reduced is the firing rate? This quantification is still lacking, and it would be interesting to know if the bursting behavior changes or remains intact.

In my opinion, the authors made a strong statement in relation to differences in the first vs second postnatal weeks that is not indispensable for supporting the main conclusion of the paper. The finding of a strong phenotype in stereocilia and MET of OE mice does not require to be paralleled with a phenotype in spiking only in the second week (although a proper quantification would always be required). I feel that the authors self-inflected the problem of demonstrating these differences.

Considering that the recordings from the IHCs are done using pre-hearing mice, the only functionally relevant AP activity is that occurring at the estimated resting membrane potential (ln. 103-122). AP activity elicited following membrane depolarization, which will not occur *in vivo*, was only used to demonstrate that IHCs from *Kir2.1-OE* mice don't lose the ability to fire APs.

We have now included the quantification of the firing activity of the IHCs at P4 in control mice (Fig 2A) using longer current clamp recordings. P4 IHCs from *Kir2.1-OE* mice do not fire APs at rest (Fig 2B). The new data is described in ln. 110-113.

To avoid unnecessary repetition, the quantification for the AP activity from control IHCs during the second postnatal week (Fig. 2C) was done using the data in Fig 7E since we performed longer current clamp recordings. This has allowed us to also compare the activity of the control IHCs (Fig. 7E) to those of *Kir2.1-OE* mice (Fig. 7F), which had recovered from DOX administration (see below). These data are now reported in ln. 232-236.

- On the Ca2+ concentration chosen for imaging experiments. The authors made a compelling justification for why endolymphatic concentration is more physiological than the perilymphatic from the standpoint of the generation and propagation from supporting cells. But action potential generation will clearly differ in IHCs at low external Ca2+. And this is a greatly important point of the study. It is true that in the end a conclusion would be made on the differences between WT and OE, whichever Ca2+ concentration is used. But this brings me back to point 1) and the importance of showing true difference in spiking activity between these two genotypes.
See the above explanation.

- The authors added interesting data in their new figure 7, which points at answering the question of whether these developmental alterations that are due to the lack of IHCs spiking activity can revert by interrupting the over-expression of the Kir2.1. Data seems nice, but similarly to what was indicated before, a real quantification of spike rates averages +/- error and statistics (not only Vm) in different genotypes and treatments is lacking.
We have now quantified the AP activity and the results are reported in ln. 232-236.

Reviewer #3
The authors have resolved all the issues I raised, in a responsive and satisfactory manner, and I have no further comments.
Thank you.

2nd Revision - Editorial Decision 28th Nov 2022

Dear Walter,

Thank you for submitting your revised manuscript to The EMBO Journal.

I have now had a chance to take a careful look at everything and I appreciate the introduced changes.

I am therefore very pleased to accept the MS for publication here.

Congratulations on a nice study!

With best wishes

Karin

Karin Dumstrei, PhD
Senior Editor
The EMBO Journal

Please note that it is EMBO Journal policy for the transcript of the editorial process (containing referee reports and your response letter) to be published as an online supplement to each paper. If you do NOT want this, you will need to inform the Editorial Office via email immediately. More information is available here:
https://www.embopress.org/page/journal/14602075/authorguide#transparentprocess

Your manuscript will be processed for publication in the journal by EMBO Press. Manuscripts in the PDF and electronic editions of The EMBO Journal will be copy edited, and you will be provided with page proofs prior to publication. Please note that supplementary information is not included in the proofs.

You will be contacted by Wiley Author Services to complete licensing and payment information. The required 'Page Charges Authorization Form' is available here: https://www.embopress.org/pb-assets/embo-site/tej_apc.pdf - please download and complete the form and return to embopressproduction@wiley.com

EMBO Press participates in many Publish and Read agreements that allow authors to publish Open Access with reduced/no publication charges. Check your eligibility: https://authorservices.wiley.com/author-resources/Journal-Authors/open-access/affiliation-policies-payments/index.html

Should you be planning a Press Release on your article, please get in contact with embojournal@wiley.com as early as possible, in order to coordinate publication and release dates.

If you have any questions, please do not hesitate to call or email the Editorial Office. Thank you for your contribution to The EMBO Journal.

** Click here to be directed to your login page: https://emboj.msubmit.net

EMBO Press Author Checklist

Corresponding Author Name: Walter Marcotti	
Journal Submitted to: EMBO Journal	
Manuscript Number: EMBOJ-2022-112118	

USEFUL LINKS FOR COMPLETING THIS FORM
The EMBO Journal - Author Guidelines
EMBO Reports - Author Guidelines
Molecular Systems Biology - Author Guidelines
EMBO Molecular Medicine - Author Guidelines

Reporting Checklist for Life Science Articles (updated January
This checklist is adapted from Materials Design Analysis Reporting (MDAR) Checklist for Authors. MDAR establishes a minimum set of requirements in transparent reporting in the life sciences (see Statement of Task: 10.31222/osf.io/9sm4x). Please follow the journal's guidelines in preparing your
Please note that a copy of this checklist will be published alongside your article.

Abridged guidelines for figures
1. Data
The data shown in figures should satisfy the following conditions:
- the data were obtained and processed according to the field's best practice and are presented to reflect the results of the experiments in an accurate and unbiased manner.
- ideally, figure panels should include only measurements that are directly comparable to each other and obtained with the same assay.
- plots include clearly labeled error bars for independent experiments and sample sizes. Unless justified, error bars should not be shown for technical
- if n<5, the individual data points from each experiment should be plotted. Any statistical test employed should be justified.
- Source Data should be included to report the data underlying figures according to the guidelines set out in the authorship guidelines on Data

2. Captions
Each figure caption should contain the following information, for each panel where they are relevant:
- a specification of the experimental system investigated (eg cell line, species name).
- the assay(s) and method(s) used to carry out the reported observations and measurements.
- an explicit mention of the biological and chemical entity(ies) that are being measured.
- an explicit mention of the biological and chemical entity(ies) that are altered/varied/perturbed in a controlled manner.
- the exact sample size (n) for each experimental group/condition, given as a number, not a range;
- a description of the sample collection allowing the reader to understand whether the samples represent technical or biological replicates (including how many animals, litters, cultures, etc.).
- a statement of how many times the experiment shown was independently replicated in the laboratory.
- definitions of statistical methods and measures:
  - common tests, such as t-test (please specify whether paired vs. unpaired), simple χ2 tests, Wilcoxon and Mann-Whitney tests, can be unambiguously identified by name only, but more complex techniques should be described in the methods section;
  - are tests one-sided or two-sided?
  - are there adjustments for multiple comparisons?
  - exact statistical test results, e.g., P values = x but not P values < x;
  - definition of 'center values' as median or average;
  - definition of error bars as s.d. or s.e.m.

Please complete ALL of the questions below.
Select "Not Applicable" only when the requested information is not relevant for your study.

Materials

Newly Created Materials	Information included in the manuscript?	In which section is the information available? (Reagents and Tools Table, Materials and Methods, Figures, Data Availability Section)
New materials and reagents need to be available; do any restrictions apply?	Yes	Material an Methods

Antibodies	Information included in the manuscript?	In which section is the information available? (Reagents and Tools Table, Materials and Methods, Figures, Data Availability Section)
For **antibodies** provide the following information:<br>- Commercial antibodies: RRID (if possible) or supplier name, catalogue number and or/clone number<br>- Non-commercial: RRID or citation	Yes	Material an Methods

DNA and RNA sequences	Information included in the manuscript?	In which section is the information available? (Reagents and Tools Table, Materials and Methods, Figures, Data Availability Section)
**Short novel DNA or RNA including primers, probes**: provide the sequences.	Not Applicable	

Cell materials	Information included in the manuscript?	In which section is the information available? (Reagents and Tools Table, Materials and Methods, Figures, Data Availability Section)
**Cell lines:** Provide species information, strain. Provide accession number in repository **OR** supplier name, catalog number, clone number, and/**OR** RRID.	Not Applicable	
**Primary cultures:** Provide species, strain, sex of origin, genetic modification status.	Yes	Material an Methods
Report if the cell lines were recently **authenticated** (e.g., by STR profiling) and tested for mycoplasma contamination.	Not Applicable	

Experimental animals	Information included in the manuscript?	In which section is the information available? (Reagents and Tools Table, Materials and Methods, Figures, Data Availability Section)
**Laboratory animals or Model organisms:** Provide species, strain, sex, age, genetic modification status. Provide accession number in repository **OR** supplier name, catalog number, clone number, **OR** RRID.	Yes	Material an Methods
**Animal observed in or captured from the field:** Provide species, sex, and age where possible**.**	Not Applicable	
Please detail **housing and husbandry conditions**.	Yes	Material an Methods

Plants and microbes	Information included in the manuscript?	In which section is the information available? (Reagents and Tools Table, Materials and Methods, Figures, Data Availability Section)
**Plants:** provide species and strain, ecotype and cultivar where relevant, unique accession number if available, and source (including location for collected wild specimens).	Not Applicable	
**Microbes:** provide species and strain, unique accession number if available, and source.	Not Applicable	

Human research participants	Information included in the manuscript?	In which section is the information available? (Reagents and Tools Table, Materials and Methods, Figures, Data Availability Section)
If collected and within the bounds of privacy constraints report on age, sex and gender or ethnicity for all study participants.	Not Applicable	

Core facilities	Information included in the manuscript?	In which section is the information available? (Reagents and Tools Table, Materials and Methods, Figures, Data Availability Section)
If your work benefited from core facilities, was their service mentioned in the acknowledgments section?	Yes	Material an Methods

Design

Study protocol	Information included in the manuscript?	In which section is the information available? (Reagents and Tools Table, Materials and Methods, Figures, Data Availability Section)
If study protocol has been **pre-registered, provide DOI in the manuscript**. For clinical trials, provide the trial registration number **OR** cite DOI.	Not Applicable	
Report the **clinical trial registration number** (at ClinicalTrials.gov or equivalent), where applicable.	Not Applicable	

Laboratory protocol	Information included in the manuscript?	In which section is the information available? (Reagents and Tools Table, Materials and Methods, Figures, Data Availability Section)
Provide DOI OR other citation details if **external detailed step-by-step protocols** are available.	Not Applicable	

Experimental study design and statistics	Information included in the manuscript?	In which section is the information available? (Reagents and Tools Table, Materials and Methods, Figures, Data Availability Section)
Include a statement about **sample size** estimate even if no statistical methods were used.	Yes	Material an Methods
Were any steps taken to minimize the effects of subjective bias when allocating animals/samples to treatment (e.g. **randomization procedure**)? If yes, have they been described?	Yes	Material an Methods
Include a statement about **blinding** even if no blinding was done.	Yes	Material an Methods
Describe **inclusion/exclusion criteria** if samples or animals were excluded from the analysis. Were the criteria pre-established?<br><br>If sample or data points were omitted from analysis, report if this was due to attrition or intentional exclusion and provide justification.	Not Applicable	
For every figure, are **statistical tests** justified as appropriate? Do the data meet the assumptions of the tests (e.g., normal distribution)? Describe any methods used to assess it. Is there an estimate of variation within each group of data? Is the variance similar between the groups that are being statistically compared?	Yes	Material an Methods

Sample definition and in-laboratory replication	Information included in the manuscript?	In which section is the information available? (Reagents and Tools Table, Materials and Methods, Figures, Data Availability Section)
In the figure legends: state number of times the experiment was **replicated** in laboratory.	Yes	Figure legends & also Material an Methods
In the figure legends: define whether data describe **technical or biological replicates**.	Yes	Figure legends & also Material an Methods

Ethics

Ethics	Information included in the manuscript?	In which section is the information available? (Reagents and Tools Table, Materials and Methods, Figures, Data Availability Section)
Studies involving **human participants**: State details of **authority granting ethics approval** (IRB or equivalent committee(s), provide reference number for approval.	Not Applicable	
Studies involving **human participants**: Include a statement confirming that **informed consent** was obtained from all subjects and that the experiments conformed to the principles set out in the WMA Declaration of Helsinki and the Department of Health and Human Services Belmont Report.	Not Applicable	
Studies involving **human participants:** For publication of **patient photos**, include a statement confirming that consent to publish was obtained.	Not Applicable	
Studies involving experimental **animals**: State details of **authority granting ethics approva**l (IRB or equivalent committee(s), provide reference number for approval. Include a statement of compliance with ethical regulations.	Yes	Material an Methods
Studies involving **specimen and field samples:** State if relevant **permits** obtained, provide details of authority approving study; if none were required, explain why.	Not Applicable	

Dual Use Research of Concern (DURC)	Information included in the manuscript?	In which section is the information available? (Reagents and Tools Table, Materials and Methods, Figures, Data Availability Section)
Could your study fall under dual use research restrictions? Please check biosecurity documents and list of **select agents and toxins** (CDC): https://www.selectagents.gov/sat/list.htm	Not Applicable	
If you used a select agent, is the security level of the lab appropriate and reported in the manuscript?	Not Applicable	
If a study is subject to dual use research of concern regulations, is the name of the **authority granting approval and reference number** for the regulatory approval provided in the manuscript?	Not Applicable	

Reporting

The MDAR framework recommends adoption of discipline-specific guidelines, established and endorsed through community initiatives. Journals have their own policy about requiring specific guidelines and recommendations to complement MDAR.

Adherence to community standards	Information included in the manuscript?	In which section is the information available? (Reagents and Tools Table, Materials and Methods, Figures, Data Availability Section)
State if relevant guidelines or checklists (e.g., **ICMJE, MIBBI, ARRIVE, PRISMA**) have been followed or provided.	Not Applicable	
For **tumor marker prognostic studies,** we recommend that you follow the **REMARK** reporting guidelines (see link list at top right). See author guidelines, under 'Reporting Guidelines'. Please confirm you have followed these guidelines.	Not Applicable	
For **phase II and III randomized controlled trials**, please refer to the **CONSORT** flow diagram (see link list at top right) and submit the CONSORT checklist (see link list at top right) with your submission. See author guidelines, under 'Reporting Guidelines'. Please confirm you have submitted this list.	Not Applicable	

Data Availability

Data availability	Information included in the manuscript?	In which section is the information available? (Reagents and Tools Table, Materials and Methods, Figures, Data Availability Section)
Have **primary datasets** been deposited according to the journal's guidelines (see 'Data Deposition' section) and the respective accession numbers provided in the Data Availability Section?	Yes	Material an Methods
Were **human clinical and genomic datasets** deposited in a public access-controlled repository in accordance to ethical obligations to the patients and to the applicable consent agreement?	Not Applicable	
Are **computational models** that are central and integral to a study available without restrictions in a machine-readable form? Were the relevant accession numbers or links provided?	Not Applicable	
If publicly available data were reused, provide the respective **data citations in the reference list**.	Not Applicable	
